# Active learning-guided mechanistic modeling reveals context-specific regulators of CXCL9 expression in pancreatic cancer cells

**DOI:** 10.1038/s44320-026-00221-w

**Published:** 2026-06-04

**Authors:** Bi-rong Wang, Maaruthy Yelleswarapu, Lucie Descamps, Federica Eduati

**Affiliations:** 1 Department of Biomedical Engineering, Eindhoven University of Technology, PO Box 513, Eindhoven, 5600MB the Netherlands; 2 Institute for Complex Molecular Systems, Eindhoven University of Technology, 5600 MB Eindhoven, The Netherlands; 3 Eindhoven Artificial Intelligence Systems Institute, Eindhoven University of Technology, 5600 MB Eindhoven, Netherlands

**Keywords:** Computational Biology

## Abstract

Cold tumors like pancreatic cancer suffer from poor immune infiltration, limiting effective anti-tumor responses. The chemokine CXCL9 promotes immune cell recruitment, but the signaling mechanisms regulating its expression in tumor cells remain poorly understood and underexplored as targets for modulation. We present a framework that integrates active learning with mechanistic logic-ODE models to guide perturbation screenings and uncover regulators of CXCL9 in pancreatic cancer cells. Using perturbation-response data and curated prior knowledge, we trained interpretable models to identify signaling mechanisms that enhance CXCL9 expression and prioritize drug combinations. Active learning enabled data-efficient model refinement and guided informative experiments under resource constraints. Benchmarking on synthetic data and experimental validation confirmed the performance of different acquisition strategies and its applicability to feasible iterative wet lab experiments. Our results demonstrate how combining active learning with mechanistic modeling supports rational, targeted experimental design.

## Synopsis

This study integrates active learning with mechanistic logic-ODE modeling to guide perturbation screening and uncover regulators of CXCL9 in pancreatic cancer cells.

- Interpretable logic-ODE models of signaling networks are trained to identify mechanisms regulating CXCL9 expression.
- Systematic benchmarking on synthetic data characterizes the active learning framework and key design choices.
- Greedy sampling prioritizes CXCL9-inducing conditions (hits), whereas uncertainty sampling enhances model learning; a combination of both provides a good compromise.
- In vitro validation demonstrates the potential of combining active learning with mechanistic modeling for targeted experimental design.

## Abstract (web summary)

This study integrates active learning with mechanistic logic-ODE modeling to guide perturbation screening and uncover regulators of CXCL9 in pancreatic cancer cells.

## Graphical abstract

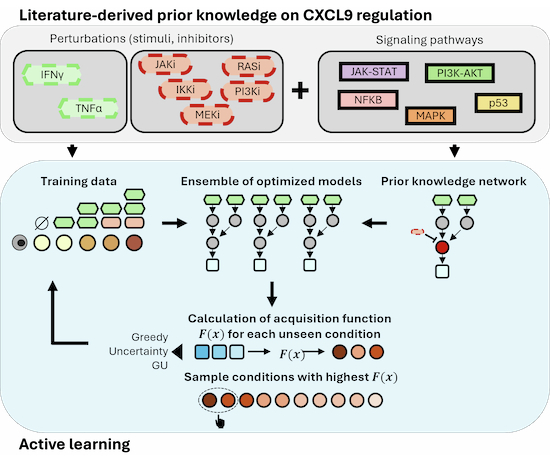

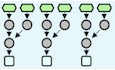

## Introduction

Pancreatic ductal adenocarcinoma (PDAC) is one of the most aggressive and lethal malignancies, characterized by late diagnosis, rapid progression, and limited treatment options (Siegel et al, 2020; Aung et al, 2018). Despite the success of immune checkpoint blockade (ICB) therapies in several other cancer types, their efficacy in cold tumors like PDAC has been disappointingly low (Mukherji et al, 2022). This is largely attributed to PDAC’s profoundly immunosuppressive tumor microenvironment (TME), which is marked by low infiltration of cytotoxic T cells and a dense stroma that supports immune evasion (Bear et al, 2020). Interestingly, rare PDAC cases exhibiting high CD8⁺ T-cell infiltration are associated with improved prognosis, highlighting the therapeutic potential of strategies that convert “cold” tumors into T-cell–inflamed phenotypes (Ino et al, 2013; Balachandran et al, 2017). In this context, the chemokine CXCL9 has drawn increasing attention as a key modulator of immune infiltration. CXCL9 recruits effector CD8⁺ T cells (Romero et al, 2020) and is associated with improved immunotherapy responses across multiple cancer types (Romero et al, 2023). Deciphering how CXCL9 expression is regulated in pancreatic cancer cells could therefore uncover actionable mechanisms to modulate the TME and improve therapeutic outcomes.

To elucidate CXCL9 regulatory mechanisms in PDAC cells, mechanistic modeling offers a valuable framework. Computational models created by combining prior knowledge of intracellular signaling pathways with experimental perturbation data can reveal causal regulatory interactions and predict the effects of targeted interventions (Saez**-**Rodriguez and Blüthgen, 2020). This approach better captures mechanistic insights compared to purely data-driven models, which focus primarily on relationships between perturbed and observed variables (Terfve et al, 2012). Among available formalisms, logic-based ordinary differential equations (logic-ODEs) strike a balance between biological interpretability and computational tractability (Wittmann et al, 2009). Logic-ODEs extend qualitative logic rules into a continuous dynamic system, allowing for the simulation of graded responses, including feedback loops, while preserving the modularity and transparency of logic models (Terfve et al, 2012). Crucially, they do not require detailed kinetic parameters, which are often unknown in cancer signaling networks, and can incorporate both literature-derived topology and context-specific experimental data (Eduati et al, 2020, 2017). This makes them particularly suitable for modeling the regulation of specific outputs, such as CXCL9 expression, in response to diverse perturbations of signaling components.

Training mechanistic models, such as logic-ODEs, requires perturbation-response data that inform how the system reacts to targeted interventions. In practice, this involves exposing cells to various stimuli or inhibitors and measuring downstream effects like CXCL9 expression. However, as signaling networks grow in size and complexity, the number of possible single and combinatorial perturbations increases exponentially, making exhaustive experimental testing unfeasible. This challenge is particularly significant when sample material is limited or when readouts are costly. Active learning provides a principled framework to address this issue by iteratively selecting the most informative experiments. In each round, new perturbations are chosen to maximize expected model improvement, allowing more efficient exploration of the experimental design space and better model generalization (Wang et al, 2024; Vasanthakumari et al, 2024). This hypothesis-driven, iterative approach has been used increasingly for biomedical applications such as compound or antibody screenings to streamline effective drug discovery (Wang et al, 2024; Balashova et al, 2025; Eisenstein, 2020). An important reason for this trend is that previous studies have demonstrated that combining active learning with machine learning models can improve performance in biological applications. In some cases, models trained with active learning outperform those trained on larger but randomly selected datasets (Vasanthakumari et al, 2024; Bertin et al, 2023). Despite this progress, active learning has not been applied in combination with mechanistic biological models. This is partly due to computational challenges and the lack of flexible software frameworks. Instead, recent efforts have focused on building surrogate models that approximate the behavior of mechanistic simulations (Gherman et al, 2023). While surrogates enable active learning in principle, they disconnect the experimental design process from the original biological model and may introduce approximation errors. By contrast, directly coupling active learning with mechanistic models allows experimental design to remain grounded in prior biological knowledge, potentially leading to more interpretable and robust discoveries.

In this study, we present an integrated experimental and computational pipeline to investigate the regulation of CXCL9 expression in pancreatic cancer cells. Starting from a prior knowledge signaling network, we trained context-specific logic-ODE models using perturbation-response data collected from two PDAC cell lines. These models enabled us to analyze the signaling mechanisms underlying CXCL9 regulation through parameter sensitivity analysis, in silico knockdowns, and bootstrapping, providing a framework for exploring context-specific regulatory interactions controlling CXCL9 expression. To extend the predictive power of these models and guide new experimental investigations, we implemented an active learning framework that directly interacts with the mechanistic model to prioritize informative perturbations. We first benchmarked multiple sampling strategies in silico, resulting in a detailed characterization of our active learning pipeline. Based on the results on synthetic data, we applied the pipeline to study CXCL9 and select promising drug combinations expected to enhance CXCL9 expression and/or improve model uncertainty. Selected perturbations were then evaluated through follow-up wet lab screenings, demonstrating the potential of the framework to use different acquisition strategies for balancing hit discovery and model generalization. Overall, this work establishes an active learning framework that can be broadly applied to different mechanistic models of intracellular signaling to support iterative and data-efficient experimental design.

## Results

### Prior knowledge network curation and experimental design

We selected AsPC1 and BxPC3, two pancreatic cancer cell lines with distinct drug response profiles based on our reanalysis of the Genomics of Drug Sensitivity in Cancer (GDSC) data set (Yang et al, 2013). AsPC1 showed lower overall sensitivity and poor correlation with BxPC3 responses (median Z-scores: 2.53 vs. 0.2; Spearman *r* = 0.19, *p* = 0.0018), suggesting the need for cell line-specific models (Appendix Fig. S1).

**Figure 1:**
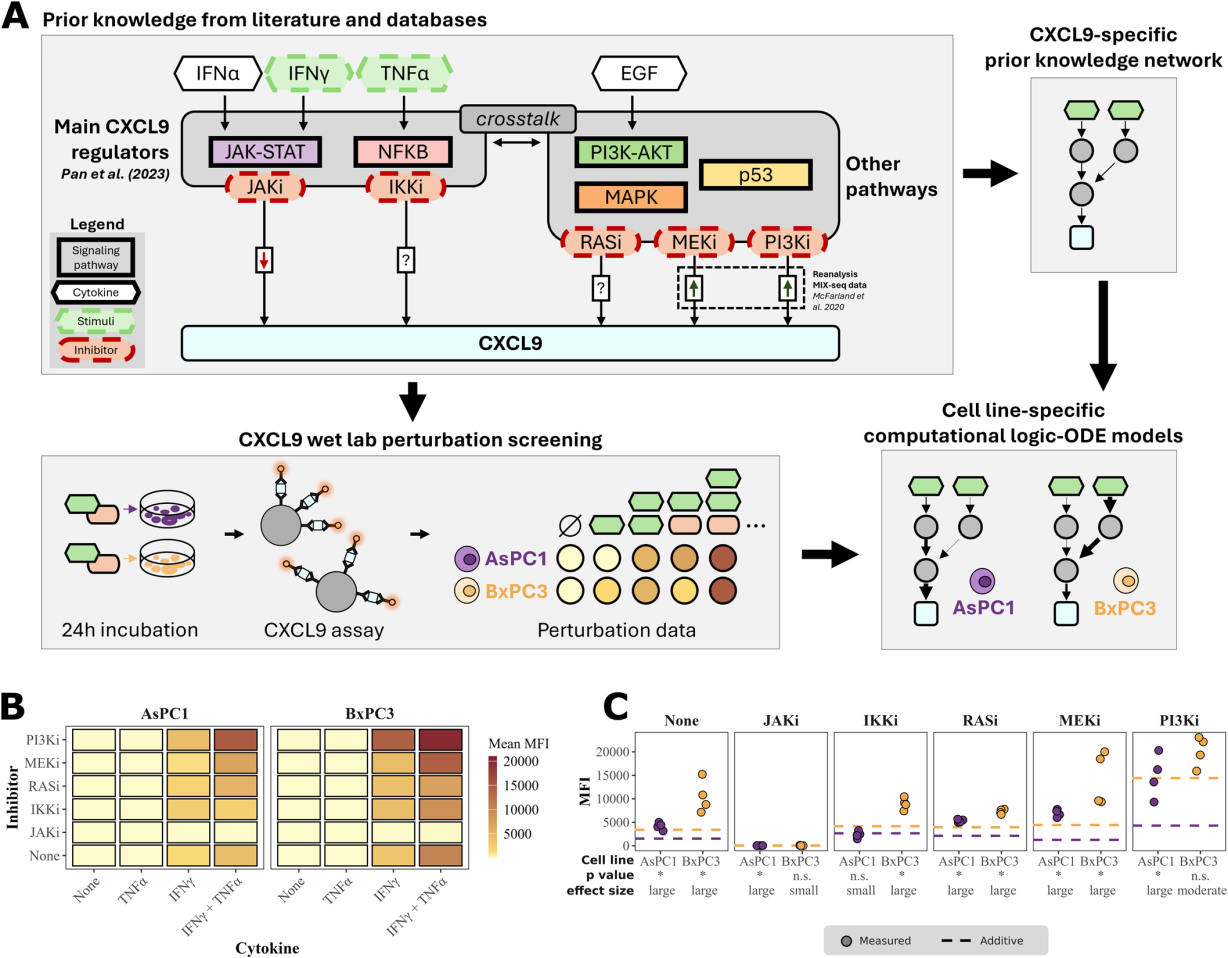
Prior knowledge network (PKN) and perturbation screenings. (**A**) Overview of input preparation for CXCL9 logic-ODE model. Prior knowledge was used to set up the perturbation screening and prior knowledge network, also giving an idea of what possible effects could be of inhibitors (red arrow = inhibitory, green arrow = stimulatory, question mark = unknown). (**B**) Measured CXCL9 levels from wet lab screenings for all screened conditions, reported as Mean Fluorescent Intensity (MFI), averaged across two independent biological replicates with technical duplicates. (**C**) Statistical analysis of IFNγ and TNFα synergy. A Wilcoxon rank-sum test compared measured values of the IFNγ + TNFα condition from the wet lab with additive values, calculated as the sum of CXCL9 responses from IFNγ and TNFα only conditions (result either not significant (*p* > 0.05) or significant (*p* < 0.05 and *p* > 0.01)). Each data point for the measured data represents a replicate (two technical replicates for both biological replicates), while the line representing the additive result is the average from summing IFNγ or TNFα only conditions. Source data are available online for this figure.

To investigate upstream regulation of CXCL9, we reviewed literature studies and reanalyzed public databases to identify relevant proteins and signaling pathways to constrain the mechanistic model. Two cytokines are well-established CXCL9 inducers: IFNγ and TNFα (Pan et al, 2023). These cytokines activate the signaling pathways JAK-STAT and NFKB, which are central to immune and inflammatory signaling (Fig. 1A; (Pan et al, 2023)). Both signaling pathways are also involved in complex crosstalk with other pathways such as PI3K-AKT, MAPK and p53, all critical pathways in cancer mechanisms. Therefore, in addition to IFNγ and TNFα stimulation, we included inhibitors targeting key components of these pathways: momelotinib (JAKi), BMS-345541 (IKKi), taselisib (PI3Ki), trametinib (MEKi), and salirasib (RASi). While JAKi and IKKi directly modulate the major CXCL9 regulators (JAK-STAT, NFKB), we observed from published transcriptomic data that PI3Ki and MEKi potentially affect CXCL9 expression (Appendix Fig. S1C,D) (McFarland et al, 2020). Additionally, RASi, though of clinical interest in pancreatic cancer, has an uncertain role in CXCL9 regulation (Sun et al, 2020). Altogether, pairwise combinations of the two cytokines and five inhibitors were used to generate perturbation-response data for model training (Fig. 1A).

We used the gathered knowledge on CXCL9 regulation to construct a prior knowledge network (PKN), which represents protein interactions relevant to CXCL9 (Fig. 1A). We included transcription factors for CXCL9 regulation identified using literature and DoRothEA (Garcia-Alonso et al, 2019). Transcription factors were linked to their upstream cytokine inducers (EGF, IFNα, IFNγ, and TNFα) via the signaling pathways described above, to create a CXCL9-specific PKN (Appendix Note—Prior knowledge network). In this network, nodes represent proteins and edges are directed interactions which can be either stimulatory or inhibitory. AND gates are a special type of node, which only affect nodes downstream if all upstream regulators are active. This PKN provides the basis for the mechanistic model, and can be further contextualized to cell line-specific dynamics using CXCL9 perturbation data.

### Shared and distinct CXCL9 responses in AsPC1 and BxPC3 cell lines

We quantified CXCL9 protein levels using a bead-based sandwich immunoassay with flow cytometry readout, across two independent wet lab screenings with duplicates per cell line (see Methods). Replicates showed on average high correlations (*R* > 0.80; Appendix Fig. S2C,D). AsPC1 and BxPC3 displayed similar patterns of CXCL9 regulation in response to perturbations, with some notable cell line-specific differences (Fig. 1B; Appendix Table S1; Appendix Fig. S2A). BxPC3 consistently showed higher CXCL9 expression than AsPC1. As expected, in both cell lines, IFNγ alone or combined with TNFα significantly upregulated CXCL9, while JAKi strongly suppressed CXCL9, confirming the key role of JAK-STAT signaling (Pan et al, 2023; Tokunaga et al, 2018; House et al, 2020). Interestingly, PI3Ki and MEKi increased CXCL9 expression, especially when combined with the stimulation of both cytokines. In contrast, IKKi suppressed CXCL9 expression in AsPC1, but only under dual cytokine stimulation, and had minimal effect in BxPC3. RASi had negligible effects on CXCL9 in either cell line, consistent with limited known involvement in CXCL9 regulation (Sun et al, 2020).

From the wet lab screenings, we observed minimal induction of CXCL9 in response to TNFα alone. However, when combined with IFNγ, the effect exceeded that of IFNγ alone, suggesting potential synergy between the two cytokines. To test this, we used the perturbation screening measurements to perform a Wilcoxon rank-sum test to assess whether the combined cytokine effect significantly exceeded the sum of the individual effects (Garcet et al, 2018; Noack et al, 2019) (Fig. 1C). Due to the limited number of biological replicates (*n* = 2), statistical analyses incorporated both biological and technical replicates to a sample size of *n* = 4. Statistical results should therefore be interpreted primarily as supportive of qualitative trends rather than statistical conclusions. Synergy was confirmed for both cell lines in the absence of inhibitors or with RASi or MEKi (*p* = 0.03, Cohen’s *d* = 0.82, for both AsPC1 and BxPC3). Interestingly, PI3Ki disrupted synergy in BxPC3 (*p* = 0.47, *d* = 0.31) but not in AsPC1 (*p* = 0.03, *d* = 0.82), while IKKi disrupted synergy in AsPC1 (*p* = 0.89, *d* = 0.10) but not in BxPC3 (*p* = 0.03, *d* = 0.82). These findings support distinct cell line-specific responses, especially to IKKi, despite overall similarities in CXCL9 regulation.

### In silico knockouts and sensitivity analysis identify key regulators and cell line-specific control of CXCL9

Next, using CellNOptR (Terfve et al, 2012) we converted our PKN into a set of logic-based ordinary differential equations (logic-ODEs) and optimized cell line-specific models for AsPC1 and BxPC3 by minimizing the mean squared difference between the model prediction and the measured CXCL9 responses from the wet lab screenings. Each optimization could, however, converge to slightly different parameter values. Therefore, for more robust model simulations, we repeated the optimization for each cell line multiple times, resulting in an ensemble of ten models, which we used for follow-up analyses. These models showed strong agreement with the experimental data (Pearson correlation, *R*² = 0.998 for AsPC1, 0.995 for BxPC3, *p* < 0.01; Appendix Fig. S2B, Appendix Table S3), showing that the models reproduce the observed perturbation responses in the training data.

**Figure 2:**
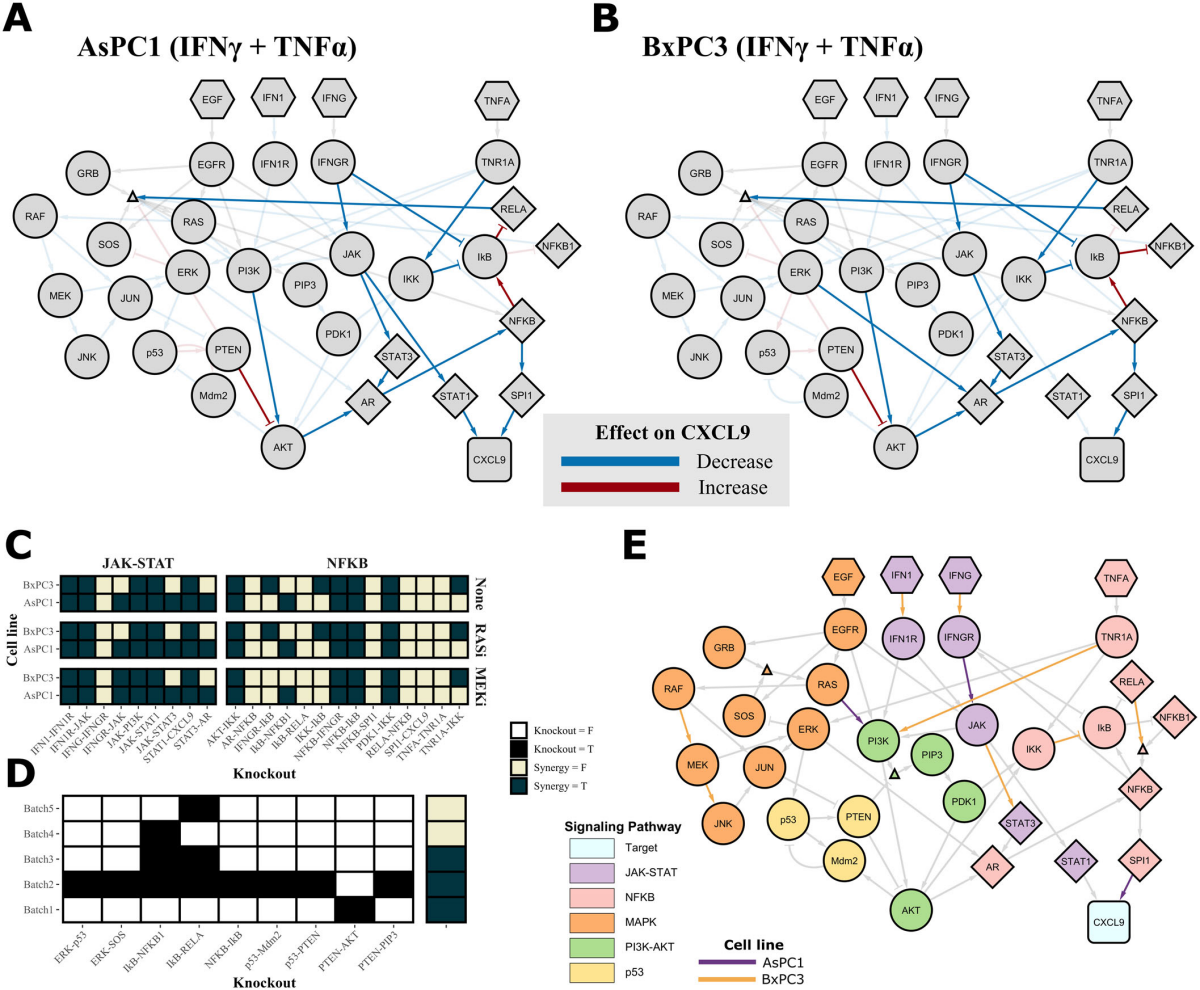
Parameter analysis of AsPC1- and BxPC3-specific models. (**A**,** B**) Effects of in silico single-edge knockouts on CXCL9 responses under IFNγ + TNFα stimulation. Significant effects (Wilcoxon rank-sum test across ten models) are highlighted separately per cell line, indicating whether CXCL9 increased or decreased. (**C**) Effect of in silico single-edge knockouts on IFNγ and TNFα synergy. Following each knockout, a significance test (Wilcoxon rank-sum test across ten models) was performed to determine whether synergy was retained (T) or lost (F). In the original (non-knockout) models, synergy was present across all visualized conditions (no inhibitor, RASi, or MEKi). (**D**) Effect of in silico knockouts of different sets of edges (batches) on IFNγ and TNFα synergy. Synergy was assessed after the removal of each batch. (**E**) Parameter comparison between AsPC1 and BxPC3 models using bootstrapped parameter values. Significant differences are highlighted in the network. Source data are available online for this figure.

Importantly, model parameters reflect the strength of regulatory interactions, making them biologically interpretable. To investigate the regulatory mechanisms underlying CXCL9 expression, we performed in silico single-edge knockouts using our trained logic-ODE models and tested for synergy using averaged model simulations. Most knockouts had limited effect, but several interactions within the NFKB and JAK-STAT pathways had a notable impact in one or both cell lines (Fig. 2A,B). Specifically, ERK-AR knockout reduced CXCL9 expression in BxPC3 (*p* = 0.004), while JAK-STAT1 and STAT1-CXCL9 knockouts affected only AsPC1 (*p* = 0.016 and *p* = 0.017, respectively). We also tested how individual knockouts influenced the synergy between IFNγ and TNFα. Interactions in the NFKB pathway were the most disruptive in both cell lines, while JAK-STAT interactions—specifically IFNGR-JAK, JAK-STAT3, and STAT3-AR—disrupted synergy only in BxPC3 (Fig. 2C), suggesting a more prominent role of JAK-STAT signaling in mediating synergy in this cell line.

Building on these findings, we simulated combinatorial knockouts to identify potential interventions that could restore synergy when it was lost. In BxPC3, synergy was disrupted under PI3Ki, likely due to elevated CXCL9 levels in the IFNγ + PI3Ki condition. Multiple single-edge knockouts, particularly in the JAK-STAT and NFKB pathways, were sufficient to restore synergy by reducing CXCL9 levels (Appendix Fig. S3). In contrast, upregulation of CXCL9 was more difficult to achieve. In AsPC1, synergy was lost upon IKKi, and only the PTEN-AKT knockout restored it at the single knockout level. We therefore tested combinations of edges that increased CXCL9 (excluding PTEN-AKT) and found that simultaneous knockouts of ERK-p53, ERK-SOS, IkB-NFKB1, IkB-RELA, NFKB-IkB, p53-Mdm2, p53-PTEN, and PTEN-PIP3 reestablished synergy (*p* = 0.014). Next, we decreased the number of knockouts and found that dual knockouts of IkB-NFKB1 and IkB-RELA restored synergy (*p* = 0.11), while individual knockouts had no significant effect (*p* = 0.97 and *p* = 0.47, respectively; Fig. 2D). These results show how mechanistic models can be used to simulate regulatory interventions that rescue context-dependent loss of cytokine synergy.

To substantiate our knockout findings, we conducted a multi-parametric sensitivity analysis (MPSA) and bootstrap resampling across 100 model fits per cell line. Key parameters within the JAK-STAT and NFKB pathways were consistently identified as major regulators of CXCL9 (Appendix Fig. S4A). Most parameters were stable across bootstrap runs, except those in the NFKB pathway, which showed greater variability, likely due to its complex feedback loops involving IKK, IkB, and NFKB. Low correlations among NFKB-related parameters (Spearman *r* between −0.25 and 0.25; Appendix Fig. S4B,C) suggest potential identifiability issues in this pathway. Finally, comparison of parameter distributions between cell lines showed general agreement, but also quantitative differences in pathway strength. Eight parameters (IFN1-IFN1R, RELA-NFKB, IKK-IkB, RAF-MEK, MEK-JNK, TNR1A-PI3K, IFNG-IFNGR, and JAK-STAT3) were significantly higher in BxPC3 (*p* < 0.05), while two (IFNGR-JAK and SPI1-CXCL9) were significantly higher in AsPC1 (*p* < 1.83E-4; Fig. 2E). These results support both the interpretability and robustness of the models and provide mechanistic insights into cell line-specific regulation of CXCL9.

### An active learning pipeline to enable iterative model refinement and efficient experimental design

**Figure 3:**
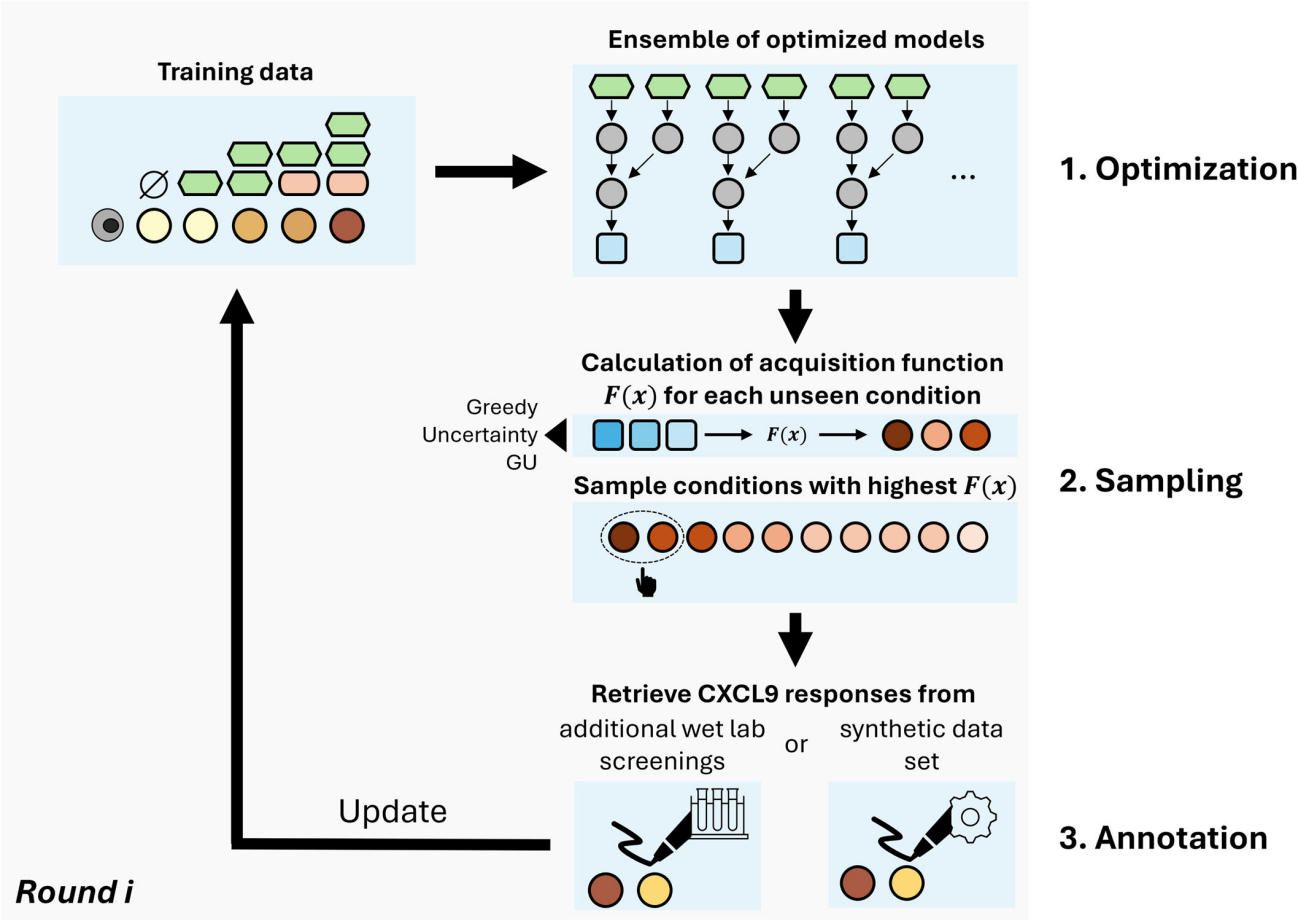
Active learning workflow. A schematic of the combination of active learning and logic-ODE models, including integration with data annotation from e.g., wet lab screenings.

Next, we implemented an active learning pipeline directly coupled with our logic-ODE models, to efficiently explore the large space of possible perturbations and prioritize experimental testing of perturbations that influence CXCL9 expression and enable model refinement. The pipeline proceeds in iterative rounds: models trained on an initial set of perturbation-response data predict CXCL9 levels for all remaining candidate conditions. Based on these predictions, a subset of new conditions is selected using an acquisition function, experimentally (or in silico) tested, and added to the training set for re-optimization of the ensemble of models. This cycle is repeated until no candidates remain or performance stabilizes (Fig. 3, see Methods for a more detailed description).

Firstly, we evaluated the impact of key design choices in this workflow, as a balance would be required between experimentally and computationally feasible design choices. For this benchmarking, we used a biologically plausible synthetic data set generated from the trained AsPC1 model, with added Gaussian noise to mimic experimental uncertainty. We systematically tested the effect of different acquisition strategies, including greedy (which favors high predicted CXCL9), uncertainty (which prioritizes conditions with high prediction variance), greedy + uncertainty (GU) (which balances both criteria), and random sampling (see Methods for formal definition). We also evaluated different numbers of conditions added per round, ranging from 2 to 10 added conditions per round, and compared several initial training sets, including manual, random, and full selections. Model performance was assessed based on hit discovery and predictive power across unseen conditions. This benchmarking step enabled us to identify acquisition strategies and configurations with favorable trade-offs for downstream application to real perturbation data.

The subsequent sections first present the in silico benchmarking results and then describe the application of the pipeline to in vitro perturbation data with experimental validation. While the in silico benchmarking provided a controlled setting to characterize acquisition functions across many active learning rounds, the in vitro experiments were designed to test whether the characteristic differences between acquisition strategies observed in silico were already reproducible under realistic laboratory conditions within the early rounds feasible in iterative wet-lab experimentation.

### Greedy sampling increases hit discovery, while uncertainty improves generalization

**Figure 4:**
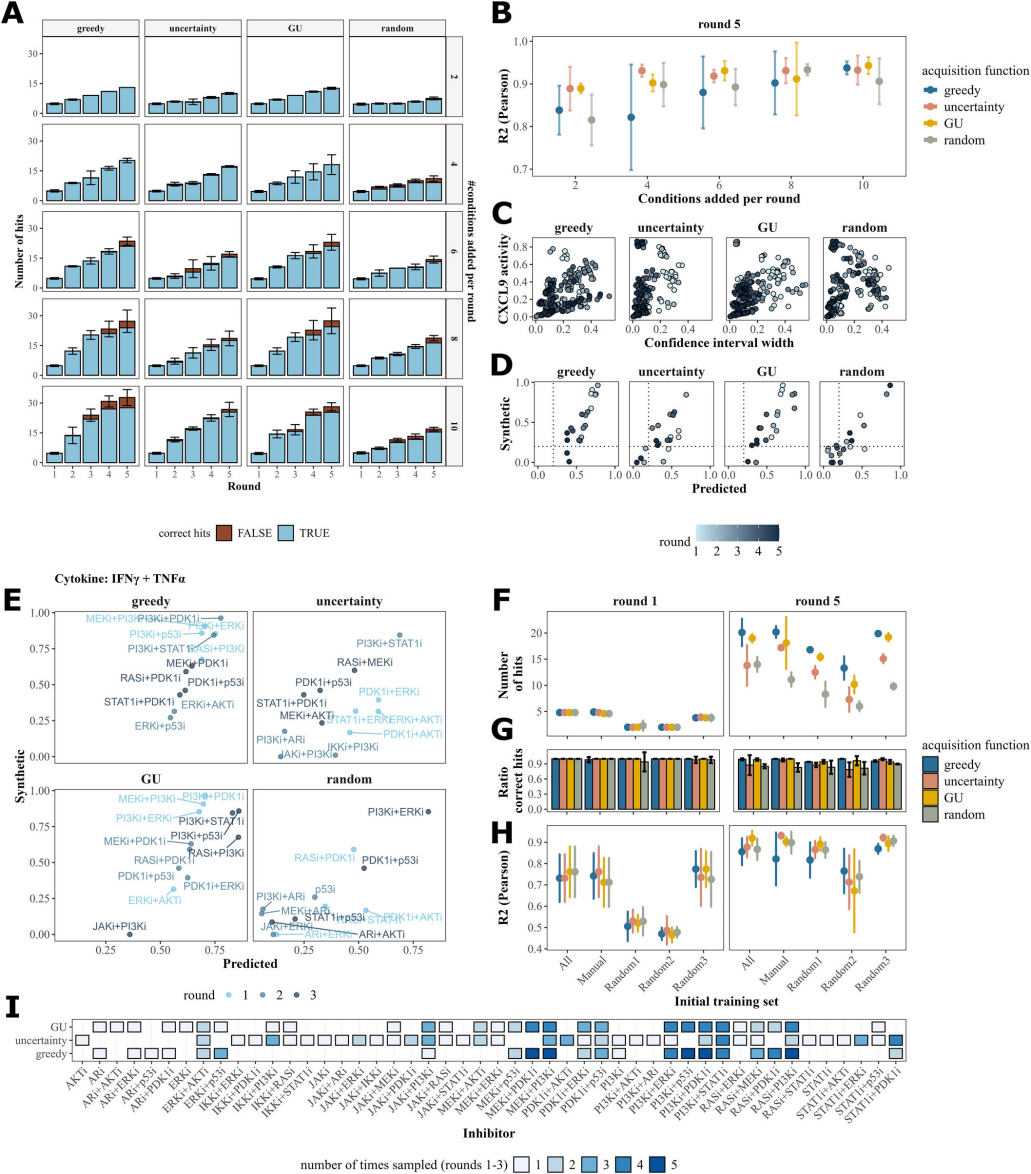
Active learning workflow with synthetic data. (**A**,** B**) Effect of the number of conditions added per round using different acquisition functions. Results are averaged across ten models per round; error bars represent standard deviation. (**A**) Hit discovery per round. From the total number of hits, colors indicate how many were correct versus incorrect. (**B**) Model generalization, represented by the Pearson correlation coefficient *R*^2^, after the final round (round 5). Mean R2 values are given, including error bars representing standard deviation across ten models. (**C**–**E**) In-depth characterization of models trained with four conditions added per round. (**C**) Comparison of mean CXCL9 activity and confidence interval width across acquisition functions. Each data point represents a condition from the selection pool in the corresponding round. (**D**) Comparison of model simulations with true CXCL9 values from the synthetic data set. Each data point represents a condition added to the training set in that round. Dashed lines indicate hit boundaries, dividing the space into regions for true/false hits and non-hits. (**E**) Inhibitor combinations of added conditions during the first three rounds. (**F**–**H**) Effect of the initial training set on (**F**) hit discovery (mean and SD across *n* = 10 models), (**G**) correct hit discovery (mean and SD), and (**H**) model generalization (mean and SD, *n* = 10 models). Round 1 is compared with round 5 to illustrate how the starting set influences outcomes. (**I**) Frequency of selection for each condition characterized by one or two inhibitors across five different initial training sets (Manual, All, Random1, Random2, Random3). Source data are available online for this figure.

Using the synthetic data set, we ran five rounds of active learning with ensembles of ten models to benchmark acquisition strategies. Greedy and GU acquisition functions identified more CXCL9-inducing conditions (“hits”) than uncertainty or random sampling (Fig. 4A; Appendix Fig. S6B). For example, after five rounds, greedy and GU achieved 1.4 to 1.9 times more hits than random sampling, while uncertainty showed a more modest and less consistent advantage (1.0 to 1.6 more hits). Although all acquisition strategies consistently increased the number of hits in the training set across rounds, the cumulative CXCL9 activity of the identified hits progressively stabilized (Appendix Fig. S6B), as the strongest conditions were preferentially identified early.

Despite identifying more hits overall, greedy and GU also generated more false positives, especially at higher numbers of conditions added per round (*N* = 6–10), as indicated by declining proportions of correctly predicted hits. In contrast, uncertainty maintained a more stable precision across different numbers of conditions added per round. These results suggest that adding a smaller number of conditions (*N* = 2 or 4) is preferable for greedy and GU to reduce false positives, while uncertainty yields more reliable predictions even at a higher number of conditions added per round. A similar trend was observed when evaluating model predictions across the full perturbation space, where uncertainty yielded the most accurate hit predictions, while greedy identified a larger number of candidate hits (Appendix Fig. S7A).

Model generalization, measured as Pearson correlation (*R*^2^) between predicted and true CXCL9 levels from the synthetic data set across all conditions, including unseen ones, at the end of the learning pipeline (i.e., round 5), was consistently highest for uncertainty-based sampling. When adding four new conditions per round, uncertainty achieved the best overall *R*² (mean = 0.93), significantly outperforming all other acquisition strategies (*p* < 0.026, Fig. 4B showing results for round 5). Notably, uncertainty already achieves high generalization performance at early stages of the active learning process (*R*² = 0.92 already at round 3; Appendix Fig. S7B). On the contrary, greedy showed lower generalization performances (mean = 0.88 at round 5) and greater variability in *R*². Notably, *R*² values on the training set remained stable across all acquisition strategies and numbers of conditions added per round, indicating consistent fit to observed data. These results emphasize that acquisition strategies strongly influence the performance of active learning with mechanistic models: greedy boosts hit discovery, uncertainty enhances generalization, and GU offers a valuable trade-off.

To assess the impact of network size, we also applied the pipeline to a reduced PKN containing only the JAK-STAT and NFKB pathways, which are the main regulatory axes of CXCL9. While this smaller model showed comparable performance on perturbations within its scope, it restricted the range of identifiable hits to this subset of signaling interactions, whereas the full PKN enabled exploration of a broader perturbation space (Appendix Fig. S9).

### Different acquisition strategies prioritize perturbations with distinct characteristics

To understand the mechanistic implications of acquisition function behavior, we examined which perturbation conditions were selected during active learning when four new conditions were added per round. We observed distinct sampling behaviors for the different acquisition functions. For instance, conditions that were selected only at later iteration rounds (Fig. 4C, darker color), had relatively low mean CXCL9 activities for greedy and low confidence interval width (CI) for uncertainty. GU maintained a balance, with values in later rounds tending to be low in both mean CXCL9 activity and CI. In contrast, random sampling did not show such trends, highlighting the advantage of acquisition functions in targeted sampling. Notably, uncertainty consistently yielded lower CIs than greedy and GU, reflecting its objective of reducing estimation variance. Additionally, comparing predicted vs. true CXCL9 responses highlighted a bias toward false positives over false negatives, as all acquisition functions, except for random sampling, selected two false positives and zero false negatives (Fig. 4D). Uncertainty showed great potential in correctly predicting also “non-hits” (i.e., conditions with low CXCL9 expression), while greedy and GU were biased toward high-response predictions. These results suggest that greedy is effective for prioritization of potential hits, but introduces estimation bias for true positive hits, whereas uncertainty offers a better balance between true positive and negative hits.

Next, we examined the selected drug combinations (Fig. 4E). As expected, acquisition functions prioritize different sets of conditions. Greedy favored combinations containing PI3Ki—which we previously identified as strong CXCL9 inducers (Fig. 1A,B)—while uncertainty focused on ERKi and AKTi combinations whose effects on CXCL9 remain uncharacterized. GU blended both approaches. The distinct sampling patterns help discover valuable candidates for further wet lab validation.

### Initial training set composition influences active learning outcome

In the preceding sections, we used an initial training set consisting of ten conditions manually selected to cover a broad range of perturbations affecting CXCL9 responses based on our experimental data (referred to as “Manual”, see Methods). To assess how the choice of initial training set influences hit discovery and model performance, we created four additional initial training sets (Appendix Fig. S6A): one including all 24 conditions from wet lab screenings (‘All’), and three randomly selected sets of ten conditions (“Random1”, “Random2”, and “Random3”).

In round 1, Random1 and Random2 had fewer predicted hits, while Manual and All and Random3 performed similarly (Fig. 4F; Appendix Fig. S6C). Across all rounds, the greedy approach consistently identified the most hits, followed by GU, uncertainty, and random sampling. Final average hit counts were highest for Manual (17) and lowest for Random2 (9), indicating that the quality of initial sampling impacts final outcomes. Despite differences in hit counts, the ratio of correct hits remained above 0.78 for all initial training sets, with random sampling yielding the most false positives (Fig. 4G). Uncertainty was the most sensitive to the initial training set, with its correct hits ratio dropping below 0.88 (Random1, Random2, and All), while the remaining ratios were significantly higher (>0.98) for the remaining initial training sets (Random3 and Manual). Especially the poorer performance with the initial training set All is interesting, although this could be due to the complete experimental data set being an imbalanced data set with more non-hits than hits. These results for uncertainty contrast with the acquisition function’s stability across varying numbers of added conditions per round (Fig. 4A), suggesting greater dependence on initial data.

Model generalizability (R²) also varied depending on the initial training set. In round 1, Random1 and Random2 showed poor model performance (*R*² < 0.53; Fig. 4H), while Random3 (*R*² = 0.75) showed performance comparable with All (*R*² = 0.75) and Manual (*R*² = 0.73). After five rounds, Random1 recovered to match other sets (*R*² = 0.86), but Random2 remained the weakest (*R*² = 0.72), likely due to a lack of key cytokine-containing conditions. GU and uncertainty showed the least variation across initial training sets (except in Random2), with either one being the best-performing model depending on the initial set, reflecting their respective strengths in generalization. We also examined the drug combinations selected by different initial sets. Greedy and GU showed consistent sampling patterns, always selecting several key PI3Ki- and MEKi-containing combinations across all setups (MEKi with PDK1i/PI3Ki and PI3Ki with p53i/PDK1i/RASi; Fig. 4I). In contrast, the uncertainty approach led to more variable selections, focusing on different areas of the search space.

Overall, while the choice of initial training sets do influence early model performance, particularly for uncertainty, the active learning pipeline can recover from suboptimal starting conditions. The Manual and All sets yielded comparable final performance, indicating that well-chosen small sets can be as effective as larger ones. Moreover, greedy and GU were more sensitive to the number of added conditions, but more stable across different initial training sets. These findings from our in silico benchmarking highlight how acquisition strategy and initial sampling interact, offering guidance for future experimental designs.

### Wet lab experiments validate differences between acquisition strategies

**Figure 5:**
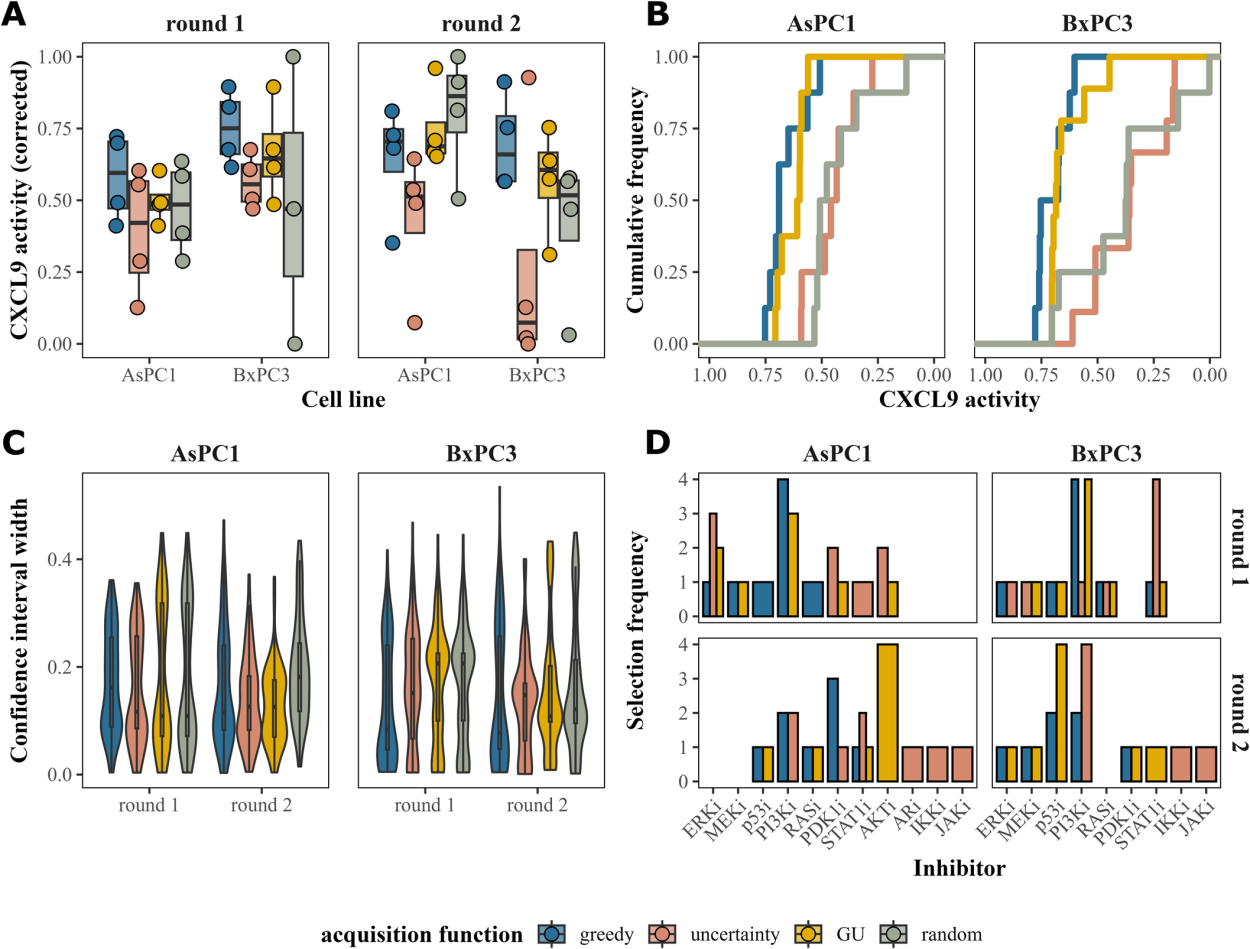
Integration of wet lab screenings in active learning workflow. (**A**) CXCL9 activities measured in AsPC1 and BxPC3 wet lab screenings for four conditions selected by different acquisition functions. Each data point represents the mean value obtained from duplicate measurements. For each set of conditions per cell line and acquisition function, box plots show the median (center line), interquartile range (IQR, box bounds; 25th–75th percentiles) and the 1.5 x IQR (whiskers). Individual data points are overlaid on each box plot, also illustrating the minima and maxima. (**B**) Cumulative distribution functions of CXCL9 activity for each cell line and acquisition function. An early rise indicates that selected conditions yielded higher CXCL9 activity values. Greater distances between curves reflect larger differences in measured activity. (**C**) Confidence interval widths from models (*n* = 10) trained on conditions sampled in rounds 1 and 2. Violin plots show the distribution of widths from model simulations across all conditions in the selection pool. Embedded box plots indicate the median (center line), interquartile range (box bounds; 25th–75th percentiles), and whiskers extending to 1.5 x the interquartile range (IQR). (**D**) Frequency of inhibitor selection, either as single agents or in combinations, across rounds and acquisition functions. A value of four indicates that the inhibitor was chosen by all acquisition strategies. Source data are available online for this figure.

After benchmarking our active learning pipeline in silico, we next applied the workflow to real experimental data by returning to the lab to measure CXCL9 responses for conditions selected through active learning, to test whether the main differences observed between acquisition strategies in silico could also be reproduced experimentally (Fig. 5). In particular, the benchmarking showed two characteristic behaviors that already emerged during the early rounds and remained qualitatively stable thereafter. First, regarding hit discovery, greedy and GU more rapidly identified high-CXCL9 conditions than uncertainty or random sampling. This difference was already visible from round 1 and remained consistent across rounds (Fig. 4A), with uncertainty sampling never reaching the cumulative CXCL9 activity achieved by greedy or GU in any round (average differences 3.44 and 3.10 across rounds for greedy and GU, respectively; Appendix Fig. S6B,C). Second, regarding model generalizability, uncertainty sampling achieved better agreement between model predictions and ground-truth data when considering both training and unseen data (Fig. 4B). These differences were also already visible during the early rounds (Appendix Fig. S7B), where the largest improvements in predictive performance occurred. Specifically, the average increase in *R*² between rounds 1 and 2 was 0.01 (greedy), 0.10 (uncertainty), and 0.11 (GU), whereas average changes across acquisition functions progressively decreased to 0.05 (round 3), 0.02 (round 4), and 0.003 (round 5). Based on these observations, and considering the practical constraints of iterative wet-lab experimentation, we focused the experimental validation on the first two active learning rounds.

Starting from the Manual initial training set, ensembles of ten models per acquisition strategy (greedy, uncertainty, GU, and random) were trained for both AsPC1 and BxPC3 cell lines and used to iteratively select four new conditions per round, which were experimentally tested for CXCL9 expression. The selected perturbations primarily involved IFNγ and TNFα stimulation combined with inhibitors targeting ERK, AKT, PDK1, STAT1, AR, and p53. CXCL9 levels were quantified via immunoassays and reported as normalized mean fluorescent intensity (MFI), consistent with previous experiments (Fig. 5A, see Appendix Fig. S8A for perturbations per condition). Similarly to the previous perturbation screenings, a high correlation is present between technical replicates (*R* > 0.95, Appendix Fig. S8B).

Several combinations, particularly those selected by greedy, resulted in lower-than-expected CXCL9 activity. For example, PI3Ki + ERKi, showed this effect in both cell lines (Appendix Note—Apoptosis Correction, Appendix Fig. S8A). We hypothesized that higher cumulative drug concentrations in combination treatments (5 μM per drug) increased cell death, thereby reducing the number of viable cells capable of secreting CXCL9. In this scenario, individual surviving cells may still express high levels of CXCL9, but the overall secretion measured in the assay is lower due to reduced cell viability. To test this, we performed a Caspase-3 (Cas3) assay to assess apoptosis levels. Strong negative Spearman correlations between Cas3 and CXCL9 were observed for conditions selected using the greedy approach (*R* = −1 and −0.8 in AsPC1 and BxPC3, respectively; Appendix Fig. S8C), while conditions selected using uncertainty had lower Cas3 values, suggesting less apoptosis.

As our current models do not account for apoptosis effects, we integrated confounding effects of apoptosis through combining normalized CXCL9 and Cas3 values, rescaling them to reflect CXCL9 secretion adjusted for apoptosis (Methods, Appendix Note—Apoptosis correction). Using the rescaled CXCL9 values, greedy-selected combinations showed the highest corrected CXCL9 levels, while uncertainty-selected combinations had the lowest. GU-selected conditions were intermediate, and random sampling showed more variability across conditions (Fig. 5A). This ordering aligns with the in silico benchmarking, where greedy and GU prioritized higher-activity conditions, while uncertainty sampling favored conditions with greater model uncertainty, often resulting in more moderate measured responses. These findings underscore the reliability of the active learning framework and its potential to guide targeted experimentation.

In round 2, rankings shifted slightly, most notably with increased hits by random sampling in AsPC1 (Fig. 5A). Still, greedy and GU consistently selected higher-activity combinations. Cumulative frequency plots confirmed this trend, greedy and GU sampled a greater number of conditions with high-CXCL9 expression (defined as normalized CXCL9 > 0.6), especially in BxPC3 (Fig. 5B). In contrast, uncertainty and random sampling explored a broader range of responses, including lower-activity conditions. The larger difference in CXCL9 activity between acquisition functions in BxPC3 compared to AsPC1 could be due to the cell line being more sensitive to perturbations, resulting in a larger selection pool of possible hits.

While uncertainty is not expected to perform as well as greedy or GU in identifying strong CXCL9 inducers, as shown in silico and validated experimentally, it may offer advantages in improving model refinement, as previously observed in silico. To evaluate this, we examined prediction confidence across all conditions over the active learning rounds (Fig. 5C). Indeed, uncertainty sampling showed the most consistent reduction in prediction uncertainty, reflected in narrower and more stable confidence interval width across conditions. Although greedy had on average lower confidence intervals, it also showed greater spread, with several conditions having much higher uncertainty, likely due to its focus on a narrower region of the perturbation space. GU again showed intermediate behavior, balancing exploration and exploitation, and further supporting its value as a trade-off between hit discovery and model refinement. Model generalization of AsPC1 in particular was consistent with the in silico benchmarking results, quantified as Pearson correlation (*R*²) between predicted and measured CXCL9 levels across all experimentally tested conditions. *R*² increased the most for uncertainty and GU from round 1 to round 2 (uncertainty: 0.70 to 0.80, GU: 0.71 to 0.81), while greedy sampling did not achieve a significant gain (*R*² = 0.71 to 0.70). For BxPC3, *R*² reached 0.84, 0.83, and 0.8 for uncertainty, GU and greedy, respectively.

Altogether, the in vitro experiments reproduced the same qualitative distinctions between acquisition strategies as the in silico benchmarking within the first two sampling rounds. Our findings validate that the acquisition strategies have distinct effects on both selected conditions and model generalization, and further demonstrate that these differences can already be captured within early experimental rounds, making iterative wet lab validation experimentally feasible.

### Distinct selected targets reflect sampling strategy behavior

Beyond validating acquisition functions with experimental data, we examined which inhibitors were most frequently selected by each sampling strategy. Frequent selection, especially by greedy or GU, may indicate biological relevance. We tallied inhibitor appearances across rounds and cell lines and observed distinct patterns (Fig. 5D). In round 1, PI3Ki was prominently selected by greedy and GU in both AsPC1 and BxPC3, appearing in every combination selected by greedy. Uncertainty showed no strong preference in AsPC1, but consistently selected STAT1i in BxPC3. Although STAT1i was expected to suppress CXCL9, screenings showed limited downregulation, possibly explaining why uncertainty prioritized it for further exploration.

By round 2, selections shifted and distinct patterns emerged between AsPC1 and BxPC3 (Fig. 5D). PI3Ki remained prominent in BxPC3, but was favored by the uncertainty acquisition function. This result might reflect how PI3Ki’s induction of CXCL9 could, while not part of the main regulatory pathways, point to interesting targets within a less-characterized search space. GU frequently selected AKTi in AsPC1 and p53i in BxPC3, both of which were validated in the wet lab screenings as CXCL9 inducers (Appendix Fig. S8). These selections may reflect either high uncertainty or expected CXCL9 activity, aligning with GU’s dual objective. Biologically, the emphasis on AKTi and p53i, both outside the canonical CXCL9 regulatory pathways, such as JAK-STAT or NFKB, suggests the involvement of less-characterized signaling mechanisms. While p53 is a tumor suppressor and not an ideal inhibition target, its apparent role in CXCL9 induction in our data set may point to indirect regulatory effects or interactions with other pathways. These observations could motivate further studies to identify alternative compounds that mimic this effect without compromising p53’s tumor-suppressive function.

## Discussion

The chemokine CXCL9 is an important driver of immune cell infiltration and has been associated with improved immunotherapy responses in several cancer types (Mishra et al, 2022; Märkl et al, 2022; Gao et al, 2020). To better understand how pancreatic cancer cells regulate CXCL9 expression, we developed an integrated experimental–computational framework that combines mechanistic logic-ODE modeling with active learning to analyze signaling mechanisms controlling CXCL9 and guide perturbation experiments.

We started by curating a CXCL9-specific prior knowledge network (PKN) and performing cytokine and inhibitor screenings, leading to cell line-specific models. These models successfully captured experimental responses, such as CXCL9 upregulation by the PI3K inhibitor taselisib, emphasizing the NFKB pathway’s central role in CXCL9 regulation. In silico knockouts revealed the NFKB pathway’s involvement in cytokine synergy, particularly in AsPC1, suggesting that restoring NFKB activity may help recover synergistic cytokine response. This finding may explain the higher sensitivity of AsPC1 to the IKK inhibitor BMS-345541, as the IKKi is more effective in cells where NFKB signaling is pivotal (Yang et al, 2006). In addition, differences between AsPC1 and BxPC3 reflect previous findings linking aberrant NFKB signaling pathway behavior to drug resistance (Mezencev et al, 2016; Pramanik et al, 2018). Parameter analysis of the fitted models revealed high variability in several NFKB-related parameters based on bootstrap analysis, suggesting limited identifiability of specific interactions within this pathway. Importantly, uncertainty-based active learning did not identify NFKB-related interactions as a dominant source of predictive uncertainty. Together, these results indicate that, despite limited parameter identifiability, the functional role of NFKB signaling is robustly captured by the model. More broadly, this illustrates how interpretable mechanistic models trained on perturbation data can reveal cell-specific regulatory features.

By coupling active learning with mechanistic modeling, we established a data-efficient strategy to identify informative perturbations under experimental constraints. Although this integration has rarely been explored due to the limited and context-specific nature of mechanistic datasets, our study shows that it is both feasible and beneficial. The curated prior knowledge network helped compensate for data scarcity by constraining the model hypothesis space, while the interpretable logic-ODE structure ensured that acquisition strategies remained mechanistically meaningful: greedy and GU focusing on CXCL9 induction, and uncertainty emphasizing model refinement. Together, these features enable interpretable, biologically grounded experimental design through active learning.

Our benchmarking experiments confirmed that active learning can be effectively applied to logic-ODE models. Simulations on synthetic data highlighted the importance of acquisition function choice, initial training set composition, and the number of conditions added per round. Even when trained on sparse data, the models were able to capture the combined effects of screened inhibitors (e.g., JAKi and PI3Ki) and generalize to unseen perturbations (e.g., ERK and p53 inhibitors), underscoring the ability of the framework to extrapolate from limited experimental conditions. Greedy and GU acquisition functions performed best for identifying CXCL9-inducing perturbations, while uncertainty-based sampling led to better generalization and model stability. After characterizing our pipeline, we applied the active learning framework with in vitro validation experiments, which revealed biologically different responses to perturbations selected by each acquisition function. Conditions prioritized by greedy and GU led to stronger CXCL9 induction, while selected by uncertainty often targeted regulators with ambiguous or poorly constrained effects, offering insights into less-characterized parts of the network and helping to refine specific regulatory interactions.

Importantly, our framework supports iterative experimental design: model predictions can be continuously refined as new data become available, enabling adaptive experimentation. We observed that even when starting from small, manually selected training sets, the models rapidly converged toward high-performing perturbation conditions over successive rounds of active learning. This positions our approach as a scalable and data-efficient strategy for systematically exploring combinatorial perturbations in signaling networks.

This promising approach also highlights several opportunities for further development and improvement of the pipeline. First, model uncertainty is currently estimated from multiple model optimizations, but more principled approaches, for example, via Bayesian parameter inference such as BayesFit (Egea et al, 2014), could offer calibrated uncertainty estimates and support more nuanced acquisition decisions. Second, while our approach enables efficient exploration of combinatorial treatments focusing on CXCL9, predictions are constrained by the structure of the prior knowledge network. The model can only simulate perturbations acting within the defined signaling axes. Expanding the PKN to include additional signaling axes, for instance, by incorporating insights from perturbation transcriptomics datasets such as MIX-Seq (McFarland et al, 2020), could broaden the hypothesis space and allow active learning to operate over a more comprehensive set of potential regulatory mechanisms. Especially as the mechanistic model is flexible for changes in the PKN, which allows for smoother integration of different mechanisms incorporated in the prior knowledge.

In addition, future studies could further strengthen the framework by expanding the experimental design to include additional biological replicates and a broader panel of cell lines, enabling more comprehensive statistical analyses of acquisition strategies across biological contexts. Independent validation of model predictions could also be incorporated, for example, by using the trained models to perform in silico knockout screenings followed by targeted experimental testing. Such validation experiments would further assess the predictive performance and generalizability of the active learning framework beyond the iterative training rounds explored here.

Finally, while this study focused on a single immunomodulatory readout (CXCL9) to set up the novel coupling between active learning and mechanistic modeling, future applications could benefit from multiplexed measurements—such as cell viability, apoptosis, or other immune-relevant axes like immunoinhibitory markers (e.g., PD-L1), stress response pathways or TGF-β. Modeling additional immunomodulatory mechanisms would allow distinguishing between different immunomodulation processes. However, this would increase model complexity and require the development of new strategies for multi-objective active learning, for example, to simultaneously maximize anti-tumor immune responses while minimizing immunosuppressive signals.

Our data revealed a negative correlation between CXCL9 secretion and apoptosis, particularly in high-dose treatments, suggesting that cytotoxicity may reduce chemokine production by depleting viable, CXCL9-producing cells. This could help explain conflicting reports linking CXCL9 levels to both favorable and unfavorable prognosis in pancreatic cancer (Huang et al, 2021; Gao et al, 2020; Pan et al, 2023). Incorporating cell viability markers into CXCL9 modeling could help disentangle these effects and better distinguish immunostimulatory from purely cytotoxic responses. To support this interpretation, we compared our results to drug sensitivity classifications from the GDSC database (Yang et al, 2013), which assesses compounds based on their ability to reduce cell viability. Combining this with CXCL9 data enables the identification of treatments with both cytotoxic and immunostimulatory effects. For example, AsPC1 cells were classified as insensitive to IKK inhibitors and showed reduced CXCL9 induction upon IKKi treatment, indicating limited benefit. In contrast, PI3K inhibition in BxPC3 moderately decreased viability while enhancing CXCL9 secretion, a more favorable dual effect. These examples highlight how integrating viability and immunomodulatory data can support more informed selection of perturbation strategies.

In summary, our framework combines mechanistic modeling with active learning to efficiently identify perturbation strategies that promote immunomodulatory responses in cancer cells. By integrating prior knowledge with experimental data, we demonstrate that interpretable, data-efficient modeling of chemokine regulation is feasible even in complex signaling contexts. The ability to iteratively refine model predictions based on targeted measurements opens new avenues for systematic exploration of treatment combinations. Beyond pancreatic cancer and CXCL9, the approach can be readily extended to other signaling outputs and cellular contexts, supporting rational, mechanism-driven design of combination therapies.

## Methods

**Table:** Reagents and tools table

Reagent/resource	Reference or source	Identifier or catalog number
**Experimental models**		
AsPC1	ATCC	CRL-1682
BxPC3	ATCC	CRL-1687
**Chemicals, enzymes and other reagents**		
Recombinant Human CXCL9 (MIG) (carrier-free)	BioLegend	578104
LEGENDplex^TM^ Human CXCL9 (MIG) Capture Bead B3, 13X	BioLegend	740994
LEGENDplex^TM^ HU Proinflam. Chemokine Panel 1 Standard	BioLegend	740987
LEGENDplex^TM^ HU Proinflam. Chemokine Panel 1 Detection Abs	BioLegend	740986
LEGENDplex^TM^ Buffer Set A	BioLegend	740368
Invitrogen^TM^ EnzChek^TM^ Caspase-3 Activity Assay Kit	Thermo Fisher Scientific	E13184
Recombinant Human IFNγ (carrier-free)	BioLegend	570204
Recombinant Human TNFα (carrier-free)	BioLegend	592702
Recombinant Human IFNα2 (carrier-free)	BioLegend	570102
Momelotinib (JAKi)	MedChem Express	HY-10961
BMS-345541 (IKKi)	MedChem Express	HY-10519
Salirasib (RASi)	MedChem Express	HY-14754
Trametinib (MEKi)	MedChem Express	HY-10999
Taselisib (PI3Ki)	MedChem Express	HY-13898
SCH772984 (ERKi)	Fisher Scientific	T6066
Pifithrin (p53i)	Fisher Scientific	T2707
MK-2206 (AKTi)	Fisher Scientific	T1952
BX-795 (PDK1i)	Fisher Scientific	T1830
Fludarabine (STAT1i)	Fisher Scientific	T1038
Enzalutamide (ARi)	Fisher Scientific	T6002
Dimethyl sulfoxide (DMSO)	Merck Life Science NV	102952
**Software**		
Cytoscape v.3.10.2		
**Other**		
BD FACSSymhony A3		
BioTek Synergy H1 Microplate Reader		

### Perturbation data

#### In vitro CXCL9 screening

Two pancreatic cancer cell lines were used for the perturbation screenings: AsPC1 and BxPC3. These were cultured using RPMI-1640 medium supplemented with 10% FBS, 1% penicillin/streptomycin, L-glutamine and phenol red. We prepared 12-well plates for the CXCL9 assays containing RPMI-1640 medium with 1E6 cells seeded per microwell. The cytokines IFNγ and TNFα were used at 10 and 1 ng/mL for AsPC1 and BxPC3, respectively. Different cytokine concentrations were used due to the higher sensitivity of BxPC3 to CXCL9 inducement.

Inhibitors were selected based on two criteria: (1) inhibitor targets a protein that could be of interest potentially for CXCL9 regulation and (2) prior studies have demonstrated clinical relevance of the inhibitor for (pancreatic) cancer treatment, or showcased a response to the inhibitor. Alternatively, we also leveraged reanalysis of existing databases to identify possible inhibitors affecting CXCL9. Based on the first criteria, we selected five targets: JAK, IKK, RAS, MEK, and PI3K. Next, we used the second criteria to select the following inhibitors: momelotinib (JAKi (Ng et al, 2019)), BMS-345541 (IKKi (Yang et al, 2006)), salirasib (RASi (Sun et al, 2020)), trametinib (MEKi (McFarland et al, 2020)) and taselisib (PI3Ki (McFarland et al, 2020)). Inhibitors, diluted in DMSO, were added at a concentration of 5 μM. All samples in an inhibitor screening contained 1% of DMSO, which was not found to affect cell population numbers significantly. See the Reagent and Tool Table for an overview of materials. A blank control is used in every screening and is defined as having no cytokines or inhibitors. Seeded cells were kept in an incubator (37 ⁰C, 5% CO₂) for 24 h before retrieving the supernatant for the CXCL9 assay.

We used the LEGENDplex™ HU Proinflam. Chemokine Panel 1 Standard Kit from BioLegend containing Human CXCL9 (MIG) Capture Beads B3, 13X (Reagent and Tool Table). This kit measures CXCL9 response through the formation of a sandwich assay complex consisting of the analyte, beads pre-coated with antibodies (diluted to 1X), biotinylated antibodies and streptavidin-phycoerythrin (SA-PE). We kept a 1:1 ratio between all assay components. The PE fluorescence was measured with the FACS Symphony A3 to quantify CXCL9. The assay was performed on a 96-well plate according to the LEGENDplex™ protocol given for this kit. Every condition was measured in duplicates.

#### CXCL9 data processing and analysis

We performed gating on the raw flow cytometry data to select the capture beads population, which were distinctive due to their intrinsic APC fluorescence. To remove outliers from this selected population, a lower limit was defined using Q1 - 1.5IQR and an upper limit using Q3 + 1.5IQR. Q1 and Q3 refer to the 25th and 75th quartiles, respectively. The IQR represents the interquartile range of the data. The mean fluorescent intensity (MFI) was calculated as the arithmetic mean. We used normalization to scale the data to a range between 0 and 1, which is a requirement for the logic models. The condition with the lowest MFI was set as the minimum (0) and the condition with the highest MFI as the maximum (1). However, to avoid saturation, we capped the highest training value at 0.8 instead of 1. With such an adjustment, the model can simulate higher CXCL9 levels than observed in the screenings. For statistical analysis, either post-hoc pairwise comparisons with the Wilcoxon rank-sum test or Spearman correlation tests were performed. For the pairwise comparisons, it was commonly a test for significant differences across means. In particular, we conducted significance tests for synergy effects, where the null hypothesis is that there is no difference in the means between the measured combination of IFNγ + TNFα and the value resulting from adding the individual IFNγ and TNFα measurements. A rejection of this hypothesis indicates a significant difference, and in the case of the measured value being higher than the additive value. For such a result, we conclude that there is significant synergy present. We corrected for multiple hypothesis testing using the Benjamini-Hochberg Procedure. These non-parametric tests were chosen due to their high robustness against non-normality. Additionally, Cohen’s d effect size was calculated as the standardized mean difference between the two groups. We selected a significance threshold of 0.05 (95% confidence interval).

#### Apoptosis assay

We measured the apoptosis marker Caspase-3 with the Invitrogen™ EnzChek™ Caspase-3 Activity Assay Kit from ThermoFisher, which includes cell lysis and quantification of Caspase-3 using Z-DEVD-R110 substrates. The fluorescent signals emitted by these substrates upon cleavage by Caspase-3 is measured with the BioTek Synergy H1 Microplate Reader set at measuring fluorescence at excitation/emission ~496/520 nm. Preparation of the samples in a 96-well plate is performed according to the protocol supplied for this kit. We corrected for apoptosis by summing the normalized CXCL9 and Cas3 assay measurements (per assay), and rescaling these values to a range of 0–1 (Appendix Note—Apoptosis correction).

### Logic-ODE model

#### Model structure

We used the logic-ODE formalism implemented in the CNORode add-on of CellNOptR (Eduati et al, 2017; Terfve et al, 2012; Wittmann et al, 2009). This approach requires two inputs: a scaffold model and experimental data. We curated a CXCL9-specific prior knowledge network (PKN) for the scaffold model. Nodes represent proteins and edges represent protein interactions. The network contains directional edges with a distinction between stimulatory and inhibitory protein interactions. The experimental data, also referred to as training data, were obtained through performing perturbation screenings measuring CXCL9 responses under different conditions. The MIDAS format, which is required for CellNOptR, encodes the experimental setup using binary values (Saez**-**Rodriguez et al, 2008). This format indicates the presence (1) or absence (0) of stimuli (e.g., cytokines) and targeted drugs. These inputs force the activity of a node to 1 if it is a stimulus or to 0 if it is a targeted molecular inhibitor. Each condition has a unique combination of perturbations.

The PKN determines the set of equations within the logic-ODE model, as an ODE is fitted to every protein (i.e., node *x*_*i*_, Equation 1). For each ODE, *B*_*i*_ is a continuous update function that indicates the regulation by *N*_*i*_ upstream nodes. The sigmoidal transfer function $f\left({x}_{i,j}\right)$ (Equation 2) contains several tunable parameters: * T*_*i*_ is the life-time of node *i*, *n*_*i,j*_, the Hill coefficient of the interaction between nodes *i* and *j*, and *k*_*i,j*_ is the strength of the regulation of node *j* on *i*. If *k*_*i,j*_=0, no regulation is present and the higher the value, the stronger the regulation. For this study, *n* was fixed at 3, while *τ* and *k* were bounded between 0 and 1 (Eduati et al, 2017; Wittmann et al, 2009). As every node has a distinct *τ* value and each edge a *k* value, this means that the number of parameters to be estimated scales with the PKN size (number of proteins and interactions). For the curated CXCL9 PKN, this corresponds to 33 *τ* and 61 *k* parameters to be optimized. The initial activities of simulated proteins were set at 0.5 to allow for both up and down regulation, except for CXCL9, which was set to 0 as no downregulation was observed experimentally.

1 $$\frac{{{dx}}_{i}}{{dt}}={\tau }_{i}\left({B}_{i}\left(f\left({x}_{i1}\right),f\left({x}_{i2}\right),\ldots ,f\left({x}_{{iN}}\right)\right)-{x}_{i}\right)$$

2 $$f\left({x}_{{ij}}\right)=1-\frac{\frac{{(1-{x}_{{ij}})}^{{n}_{{ij}}}}{{(1-{x}_{{ij}})}^{{n}_{{ij}}}+{k}_{{ij}}^{{n}_{{ij}}}}}{\frac{1}{1+{k}_{{ij}}^{{n}_{{ij}}}}}$$

The CellNOptR workflow consists of the following steps: (step 1) import PKN and training data, (step 2) process network, and (step 3) model training. In (step 2), the complexity of the given logic model is reduced through compression while still retaining logical consistency (Saez**-**Rodriguez et al, 2009). In step 3, model optimization is performed using the toolbox MEIGO with the metaheuristic enhanced scatter search. Advantages of this stochastic global optimization method include its robustness and ability to tackle complex optimization problems (Egea et al, 2014). However, as each optimization could therefore also converge to slightly different parameter values, we repeated model optimization ten times on the same data set. For follow-up simulations and analyses, we average model outputs across the ensemble of models. The loss function for model optimization calculates the mean squared deviation between model prediction and training data and includes L1 regularization for the *k* parameter (Eduati et al, 2017; Terfve et al, 2012). The L1 regularization term has a weight factor λ and sums the absolute values of every optimized *k* parameter. λ = 1e-10 was used during optimization (see Appendix Fig. S5 on the effect of the regularization term on model parameters). Additionally, each model is required to have a best objective function value below 0.5 to ensure proper convergence.

#### Prior knowledge network

We curated a CXCL9-specific PKN to act as a scaffold network for CellNOptR. This PKN was curated via the following steps: (1) determining relevant transcription factors (TF) of CXCL9 that have a direct binding (i.e., TF binds to the DNA site on the gene) and functional binding (i.e., binding induces protein up-/down-regulation) to the CXCL9 gene, (2) determining cytokines that affect CXCL9 levels, and (3) connecting TF and cytokines through established signaling pathways. The resulting PKN was validated through a qualitative analysis, where we tested if simple effects from literature can be simulated correctly. See Appendix Note—Prior knowledge network for a more detailed description of PKN curation and validation. The final network consists of proteins from five major signaling pathways: JAK-STAT, NFKB, PI3K-AKT, MAPK, and p53. For the logic-ODE, this PKN constrains our model by limiting the number of parameters to estimate.

#### Parameter analysis

We compared parameter values between cell line-specific models using parameter distributions retrieved through bootstrapping, i.e., repeated optimization using sampled data with replacement (Joshi et al, 2006). We performed pairwise comparisons of the *k* parameter distributions from the two cell line-specific models. This was done with the Wilcoxon rank-sum test and a significance threshold of 0.05. Additionally, we performed two types of parameter analyses: a global sensitivity analysis and in silico knockout experiments.

We implemented multi-parametric sensitivity analysis (MPSA) as described by Zi (Zi, 2011). Our goal was to retrieve high-level insights into sensitive model parameters for CXCL9. Latin hypercube sampling is used to generate different model parameter sets based on a parameter set from an optimized model. We did not fix any *k* parameter values and varied all of them for each parameter set. The resulting sum of squared differences (SSD) between CXCL9 model outputs and training data was compared with the SSD of the original parameter set. The differences were used to calculate the Kolmogorov–Smirnov (KS) statistic. The higher this value, which ranges between 0 and 1, the more impact the variation of a parameter has on the model output. The mean of dummy parameters is used to create a threshold between significant and non-significant KS statistics. Furthermore, we analyzed protein interactions and their relevance to CXCL9 responses with in silico knockouts. Knockouts were mimicked in the model by setting the *k* parameter of an edge to 0. We compared simulations of the adjusted model with the original model. The bigger the difference, the bigger the impact of the knocked-out interaction on the CXCL9 response.

### Active learning workflow

We developed an active learning pipeline to select the new conditions to test in the wet lab. Within the active learning loop, a set of trained models predicts CXCL9 responses for unseen conditions in the candidate set. A subset of conditions is selected based on an acquisition function. After retrieving measurements from the wet lab, these conditions are added to the training set and removed from the candidate set. The previously trained models are further optimized on the updated training set. We define the moment in which the data sets change as the initialization of a new round within the active learning process. The loop continues with the next round until the candidate set is empty or if an early stopping criteria has been met.

#### Sampling

In each round of the active learning workflow, samples from the candidate set are selected by ranking conditions based on scores computed with an acquisition function. This function takes the predictions of an ensemble of models into account. Different types of acquisition functions are used, based on prior work by Vasanthakumari et al (2024), who investigated active learning strategies for anti-cancer drug screenings (Vasanthakumari et al, 2024). Additionally, we also used random sampling as a baseline for comparisons. Acquisition functions use either the mean (*μ*) and/or the 95% confidence interval width of the model output (CI_width_). The confidence interval is calculated using simulations from an ensemble of models. We distinguished three types of acquisition functions:

- Greedy: F(x) = μ(x), which considers the mean of the predictions. If a sample has a high greedy F(x) value, the corresponding perturbations are predicted to induce an upregulation of CXCL9.
- Uncertainty: F(x) = CI_width_(x), which considers the confidence interval of the model output. It represents the uncertainty of a prediction.
- GU: F(x) = μ(x) + CI_width_(x), which is a combination of the greedy and uncertainty approaches.

#### Synthetic data set

The active learning loop contains several decisions that can impact the quality of the optimization. To enable systematic analysis of different configurations across multiple rounds, something not feasible with in vitro experiments, we generated a biologically plausible synthetic data set. This systematic benchmarking enables a detailed characterization of the active learning framework and provides guidance on how to apply it in combination with in vitro experiments. The synthetic data set consists of a simulation of a set of conditions using the AsPC1 model. Gaussian noise was added to the values with a mean of 0 and standard deviation of 0.2 to simulate experimental noise. The synthetic data set was used to systematically assess the impact of varying the number of conditions added in each round and the selected conditions in the initial training set. When choosing the number of conditions to add, we want to create a balance between efficiency and feasibility in the wet lab. The initial training set is important, as the advantage of active learning is that it is unnecessary to use all conditions from the perturbation screenings. However, it is unclear what the effect is of the initial training set on model convergence. Therefore, we investigated this effect using five different initial configurations, ranging from handpicked conditions (“Manual”) to random sampling. For Manual, we selected conditions based on the following criteria:

1. Include condition with no perturbations (no cytokines or inhibitors)
2. Include conditions describing effects of cytokines on CXCL9 in the absence of inhibitors (IFNγ, TNFα or IFNγ, and TNFα only)
3. Include an inhibitor with an inhibitory effect on CXCL9 (JAKi)
4. Include one or two inhibitors with a stimulatory effect on CXCL9 (PI3Ki and MEKi)
5. Include inhibitors that had an effect only in one of the two cell lines (IKKi)

#### Evaluation

Models trained with the active learning approach can be evaluated on different criteria: (1) the rate of detecting hits, where a hit is defined as a condition with a CXCL9 value above a specific threshold (0.2), and (2) the model prediction performance. For hit discovery, we valued both efficiency in detecting hits (i.e., hits are sampled in the early rounds) and correctness of predicted hits. We represented model performance for each round with the Pearson correlation coefficient (*R*^2^).

## Supplementary information

Appendix

Peer Review File

Source data Fig. 1

Source data Fig. 2

Source data Fig. 4

Source data Fig. 5

Appendix Source Data 5

## Data Availability

All reagents used in this study are commercially available, and suppliers are provided in the Methods section. Data and additional information for perturbation experiments is provided at https://github.com/SysBioOncology/ActiveLearning_MechanisticModels_CXCL9. All code to ensure reproducibility of the results is available at https://github.com/SysBioOncology/ActiveLearning_MechanisticModels_CXCL9. This includes preparation of the perturbation screening data, model training, active learning pipeline and parameter analyses such as in silico knockouts, sensitivity analysis (MPSA), and bootstrapping. Scripts to reproduce the main figures in this manuscript are also included. Installation information and package versions used for the manuscript are available in the repository. The source data of this paper are collected in the following database record: biostudies:S-SCDT-10_1038-S44320-026-00221-w.
