# Supplementary material for: Active learning-guided mechanistic modeling reveals context-specific regulators of CXCL9 expression in pancreatic cancer cells
Source: Mol Syst Biol. 2026 Jun 4;22(8):1360–75. doi: 10.1038/s44320-026-00221-w (PMC13434641; doi:10.1038/s44320-026-00221-w)
Supplement: Supplementary file 1 [file 44320_2026_221_MOESM1_ESM.pdf]

Appendix for

Active learning-guided mechanistic modeling reveals context-specific regulators of CXCL9 expression in pancreatic cancer cells

Contact information
Lead contact: Eduati F. (f.eduati@tue.nl)

TABLE OF CONTENTS

Appendix Figure S1 Analysis of GDSC and MIX-Seq data sets 2
Appendix Figure S2 Additional results for cell lines 3
Appendix Table S1 Mean fluorescent intensities 4
Appendix Figure S3 In silico single-edge knockouts 5
Appendix Figure S4 In-depth parameter analyses 6
Appendix Figure S5 Effect of L1 regularization 7
Appendix Figure S6 Varying number of added conditions and initial training sets 8
Appendix Figure S7 Evaluation of model performances across active learning rounds 9
Appendix Figure S8 Experimental data of selected conditions 10
Appendix Table S2 Mean fluorescent intensities AL 12
Appendix Figure S9 Small PKN 14
Appendix Note - Prior knowledge network 15
Appendix Table S3 Parameter values of original AsPC1 and BxPC3 models 17
Appendix Table S4 Parameter values of AsPC1 active learning models 19
Appendix Table S5 Parameter values of BxPC3 active learning models 21
Appendix Note - Apoptosis correction 23
Appendix References 24

Appendix Figure S1 Analysis of GDSC and MIX-Seq data sets

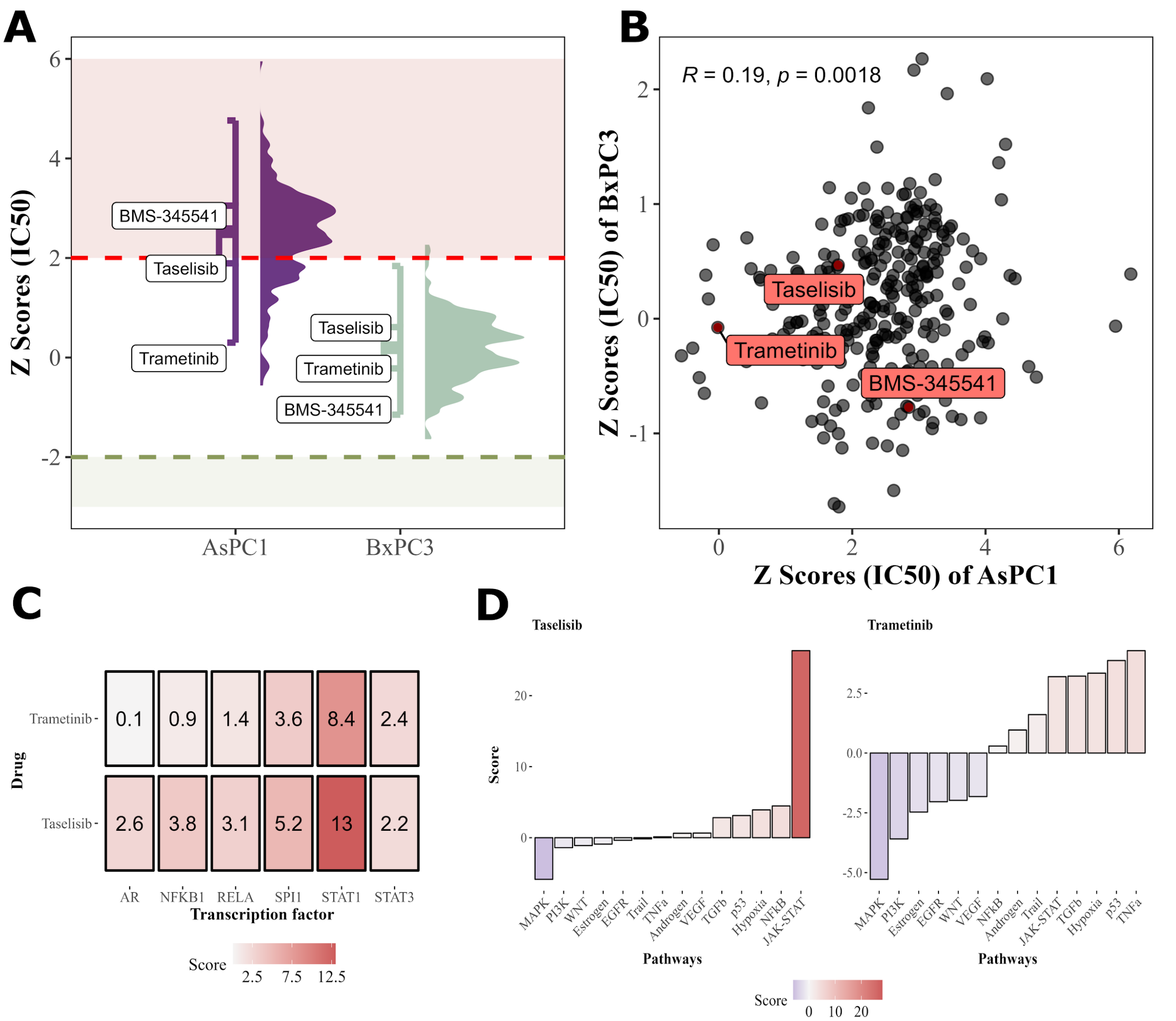

Appendix Figure S1. Analysis of GDSC and MIX-Seq data sets (A) Z-scores of the IC50 from the GDSC dataset for AsPC1 and BxPC3. Three drugs are highlighted, taselisib (PI3Ki), BMS-345541 (IKKi) and trametinib (MEKi). The GDSC dataset defined Z-scores <-2 as being sensitive, while Z-score > 2 represents drugs to which cell lines are insensitive. **(B)** Z-scores of IC50 from GDSC plotted with each axis representing AsPC1 or BxPC3. The Spearman Rank Correlation was used to compare the two cell lines. **(C)** Transcription factor (TF) activities for BxPC3 retrieved with DoRothEA for the TF regulating CXCL9 (Garcia-Alonso *et al*, 2019). Activity inference was performed using an Univariate Linear Model. A positive value represents increased activity with respect to the control experiment from the differential analysis. **(D)** Signaling pathway activities for BxPC3 inferred with PROGENy using the Multivariate Linear Model (Schubert *et al*, 2018). A positive score represents increased activity compared to the control.

Appendix Figure S2 Additional results for cell lines

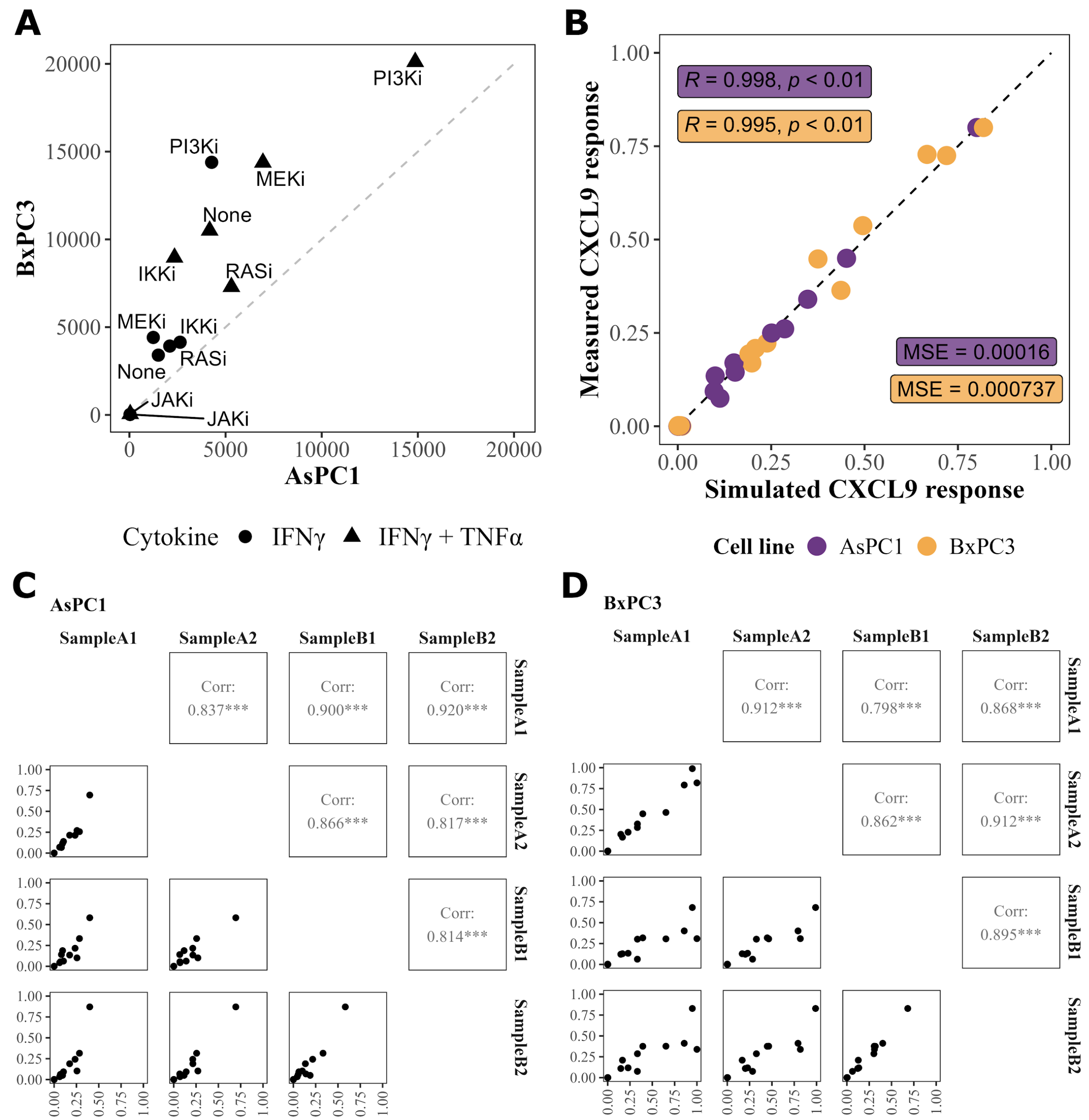

Appendix Figure S2. Additional results for cell lines (A) Comparison of measured CXCL9 in AsPC1 and BxPC3 cell lines from wet lab screenings. The dotted line indicates y=x. **(B)** Model fitting results of AsPC1 and BxPC3, comparing simulated and measured CXCL9 responses. The dotted line represents y=x, increased proximity of data to this line reflects better model fitting. **(C-D)** Correlation between replicates of both biological (Sample A, B) and technical (Sample 1, 2) replicates. Spearman correlations are included.

Appendix Table S1 Mean fluorescent intensities

Appendix Table S1: Mean fluorescent intensities of AsPC1 and BxPC3 measurements, source data of Figure 1B

Cytokine	Inhibitors	MFI	sd	Cell line	label
IFNγ	IKKi	2635.06	1340.899	AsPC1	2635.06
IFNγ	JAKi	31.51734	5.575555	AsPC1	31.52
IFNγ	None	1503.001	168.472	AsPC1	1503
IFNγ	RASi	2098.129	816.8285	AsPC1	2098.13
IFNγ	PI3Ki	4276.513	2176.002	AsPC1	4276.51
IFNγ	MEKi	1240.602	395.0651	AsPC1	1240.6
IFNγ + TNFα	IKKi	2345.462	739.7692	AsPC1	2345.46
IFNγ + TNFα	JAKi	41.01859	7.996724	AsPC1	41.02
IFNγ + TNFα	None	4171.211	777.314	AsPC1	4171.21
IFNγ + TNFα	RASi	5303.262	331.7407	AsPC1	5303.26
IFNγ + TNFα	PI3Ki	14861.38	4614.592	AsPC1	14861.38
IFNγ + TNFα	MEKi	6942.444	786.7661	AsPC1	6942.44
None	IKKi	26.45175	4.27202	AsPC1	26.45
None	JAKi	30.66988	4.150403	AsPC1	30.67
None	None	28.29937	3.103326	AsPC1	28.3
None	RASi	28.22567	5.343646	AsPC1	28.23
None	PI3Ki	29.39907	4.57831	AsPC1	29.4
None	MEKi	32.41582	2.754847	AsPC1	32.42
TNFα	IKKi	28.6216	3.865832	AsPC1	28.62
TNFα	JAKi	29.14795	4.568804	AsPC1	29.15
TNFα	None	27.48589	4.104912	AsPC1	27.49
TNFα	RASi	29.02447	5.603252	AsPC1	29.02
TNFα	PI3Ki	26.23725	5.315408	AsPC1	26.24
TNFα	MEKi	24.97547	4.186669	AsPC1	24.98
IFNγ	IKKi	4140.864	1390.733	BxPC3	4140.86
IFNγ	JAKi	25.98325	3.531241	BxPC3	25.98
IFNγ	None	3404.243	958.7296	BxPC3	3404.24
IFNγ	RASi	3921.2	775.2085	BxPC3	3921.2
IFNγ	PI3Ki	14391.62	8072.974	BxPC3	14391.62
IFNγ	MEKi	4413.225	3257.224	BxPC3	4413.23
IFNγ + TNFα	IKKi	8965.572	1245.518	BxPC3	8965.57
IFNγ + TNFα	JAKi	53.67485	38.0492	BxPC3	53.67
IFNγ + TNFα	None	10505.67	3492.608	BxPC3	10505.67
IFNγ + TNFα	RASi	7294.399	504.5485	BxPC3	7294.4
IFNγ + TNFα	PI3Ki	20114.9	3219.205	BxPC3	20114.9
IFNγ + TNFα	MEKi	14376.19	5673.471	BxPC3	14376.19
None	IKKi	24.42004	0.688525	BxPC3	24.42
None	JAKi	26.16762	3.915039	BxPC3	26.17
None	None	19.76502	1.105655	BxPC3	19.77
None	RASi	21.43759	1.196884	BxPC3	21.44
None	PI3Ki	54.54781	29.73917	BxPC3	54.55
None	MEKi	34.69997	11.23594	BxPC3	34.7
TNFα	IKKi	23.96506	4.658422	BxPC3	23.97
TNFα	JAKi	24.43282	1.479993	BxPC3	24.43
TNFα	None	23.17732	1.355325	BxPC3	23.18
TNFα	RASi	23.94257	2.095527	BxPC3	23.94
TNFα	PI3Ki	22.7971	1.690566	BxPC3	22.8
TNFα	MEKi	24.27299	2.37227	BxPC3	24.27

Appendix Figure S3 In silico single-edge knockouts

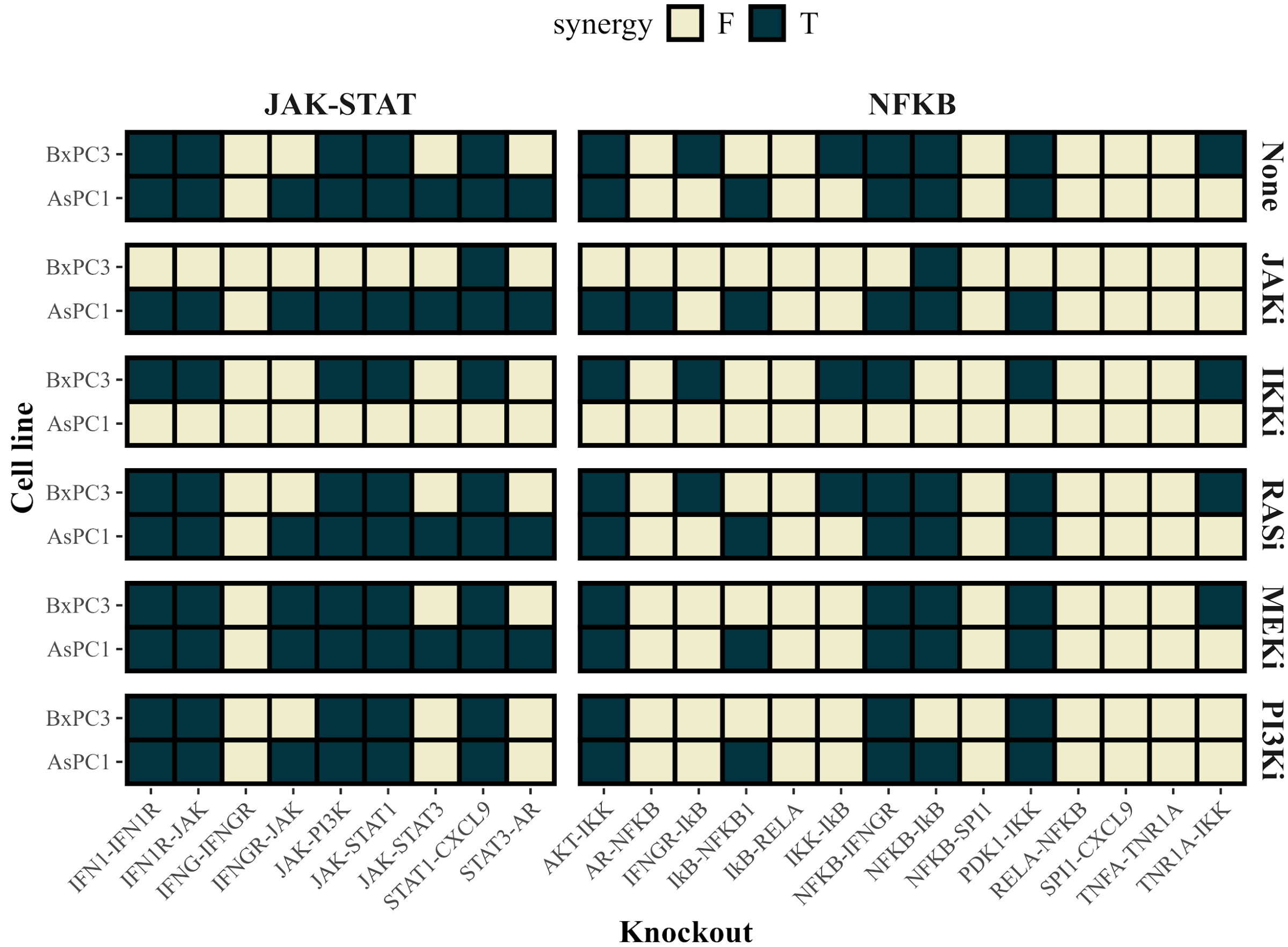

Appendix Figure S3. In silico single-edge knockouts All single-edge knockouts of the JAK-STAT and NFKB pathway, and their effect on synergy (F = no synergy, T = synergy) for each inhibitor.

Appendix Figure S4 In-depth parameter analyses

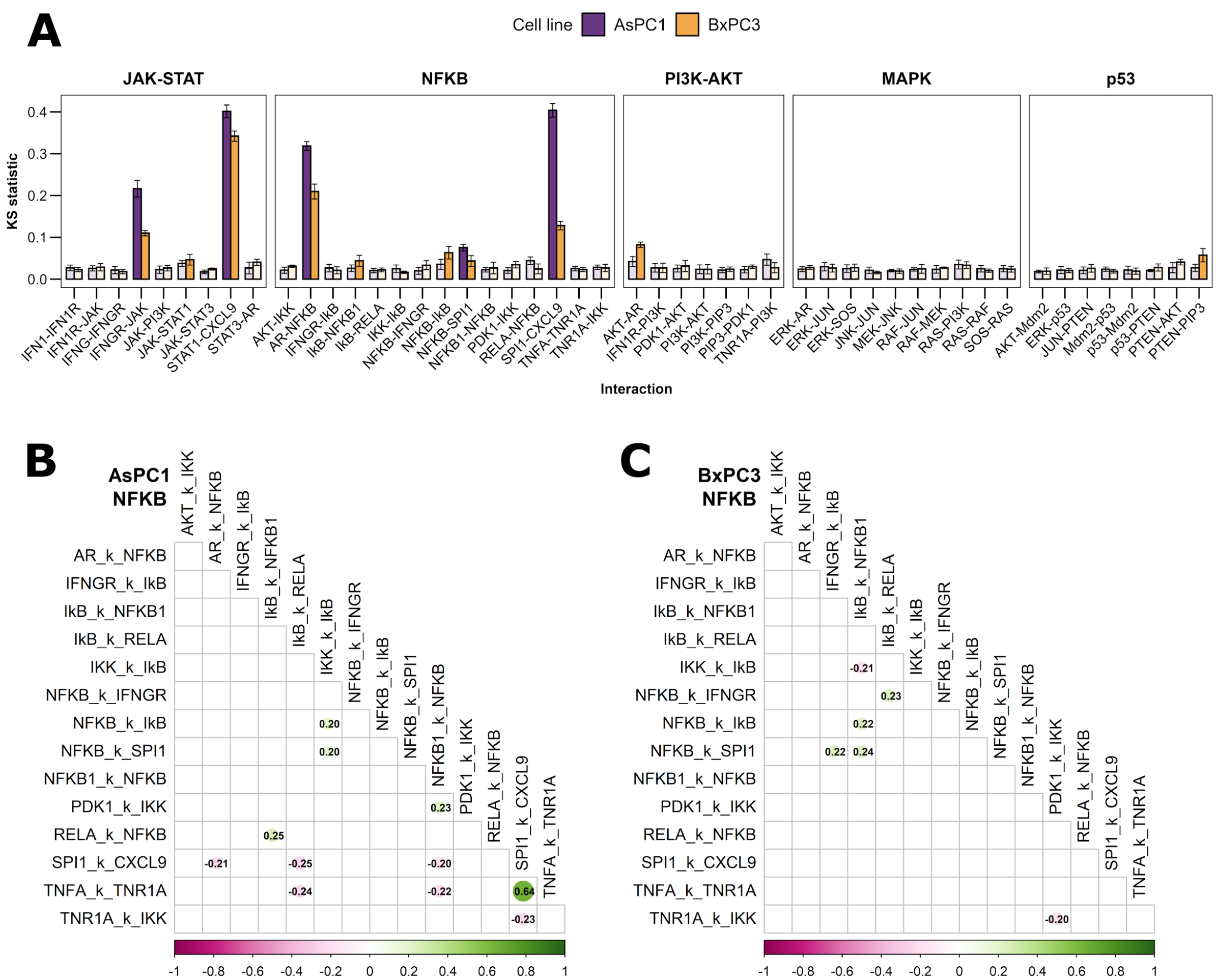

Appendix Figure S4. In-depth parameter analyses (A) Multi-parametric sensitivity analysis of both pancreatic cancer cell lines for varying parameter values with +/- 0.3. Parameter values are sampled using Latin hybercube sampling. Each bar represents the mean across five MPSA runs and the error bar the standard deviation. Parameters that are highlighted have a higher KS statistic compared to the mean of the dummy parameters. The number of samples (20 000) was determined using dummy parameters. **(B-C)** Parameter correlations (Pearson) within the NFKB pathway (α = 0.05) for both cell lines. Only significant correlations are included in the visualization.

Appendix Figure S5 Effect of L1 regularization

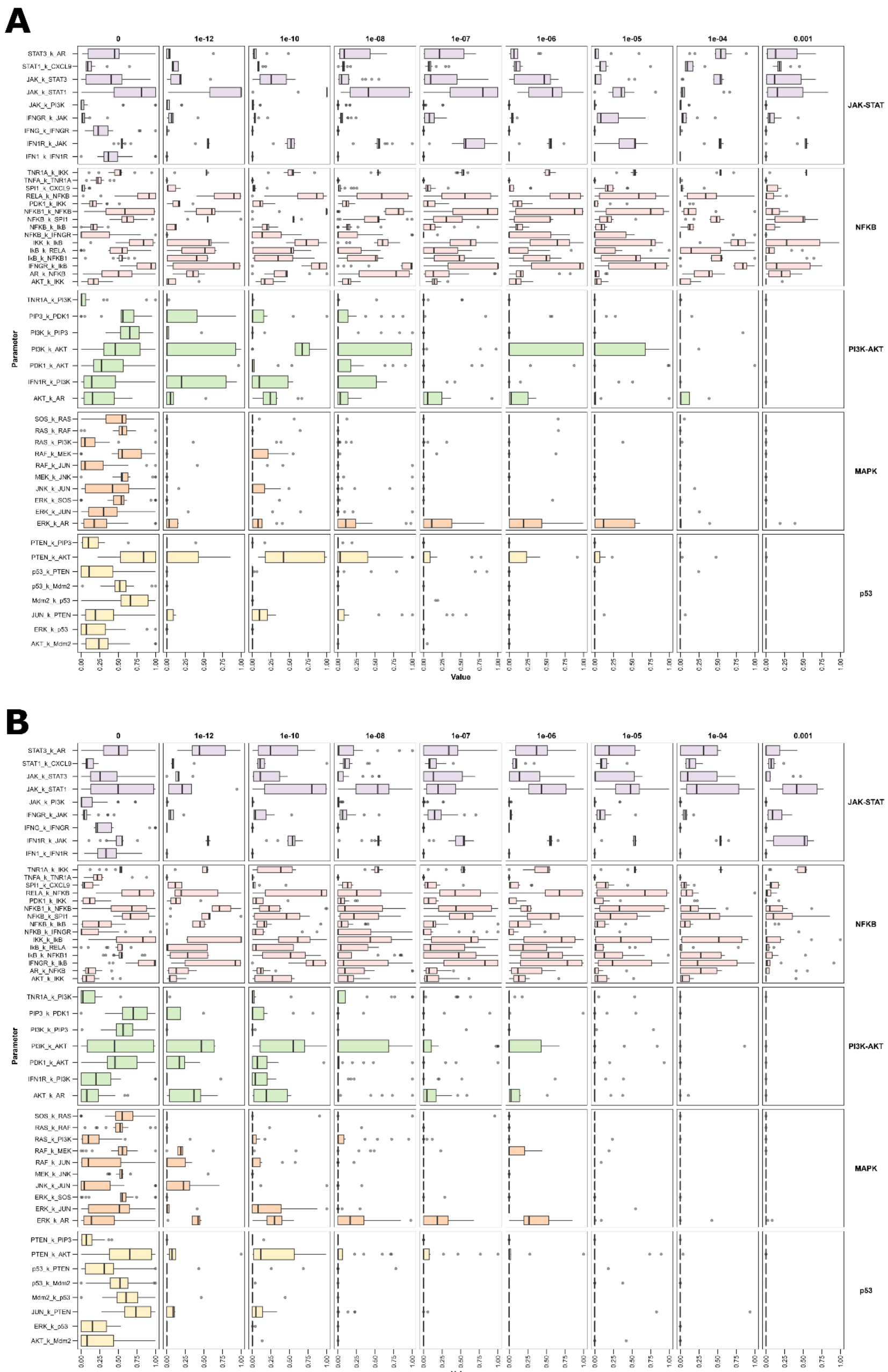

Appendix Figure S5. Effect of L1 regularization AsPC1 **(A)** and BxPC3 **(B)** models were trained with different L1 regularization terms. A comparison is visualized of the impact of L1 regularization on parameters of ten optimized models categorized by signaling pathway.

Appendix Figure S6 Varying number of added conditions and initial training sets

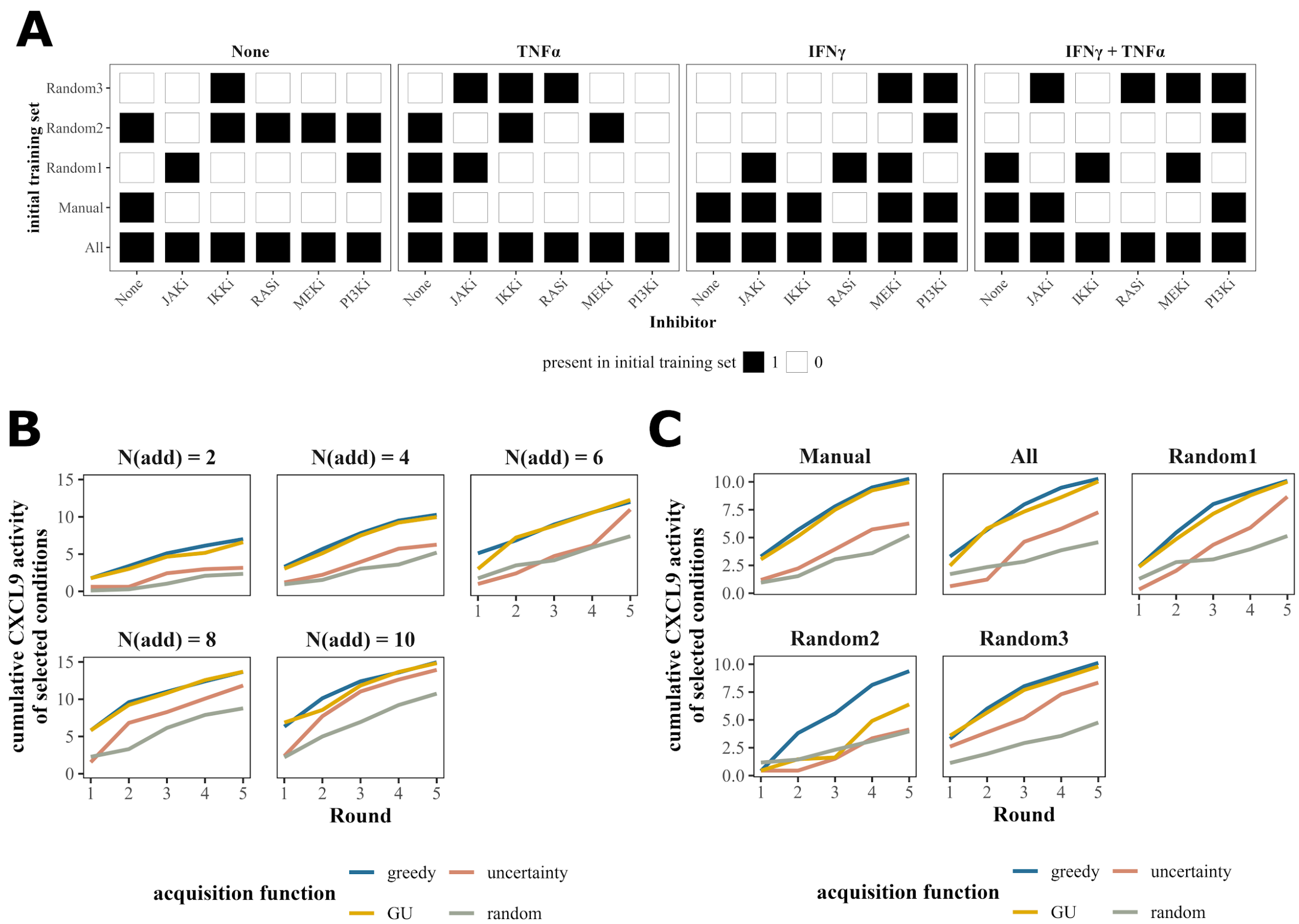

Appendix Figure S6. Varying number of added conditions and initial training sets (A) Conditions present in each of the initial training sets tested using the synthetic data. **(B-C)** Comparing cumulative CXCL9 activities of selected conditions for differing number of conditions added per round (N(add), **(B)**) or initial training sets **(C)**). CXCL9 activities of the synthetic data set are provided for each acquisition function.

Appendix Figure S7 Evaluation of model performances across active learning rounds

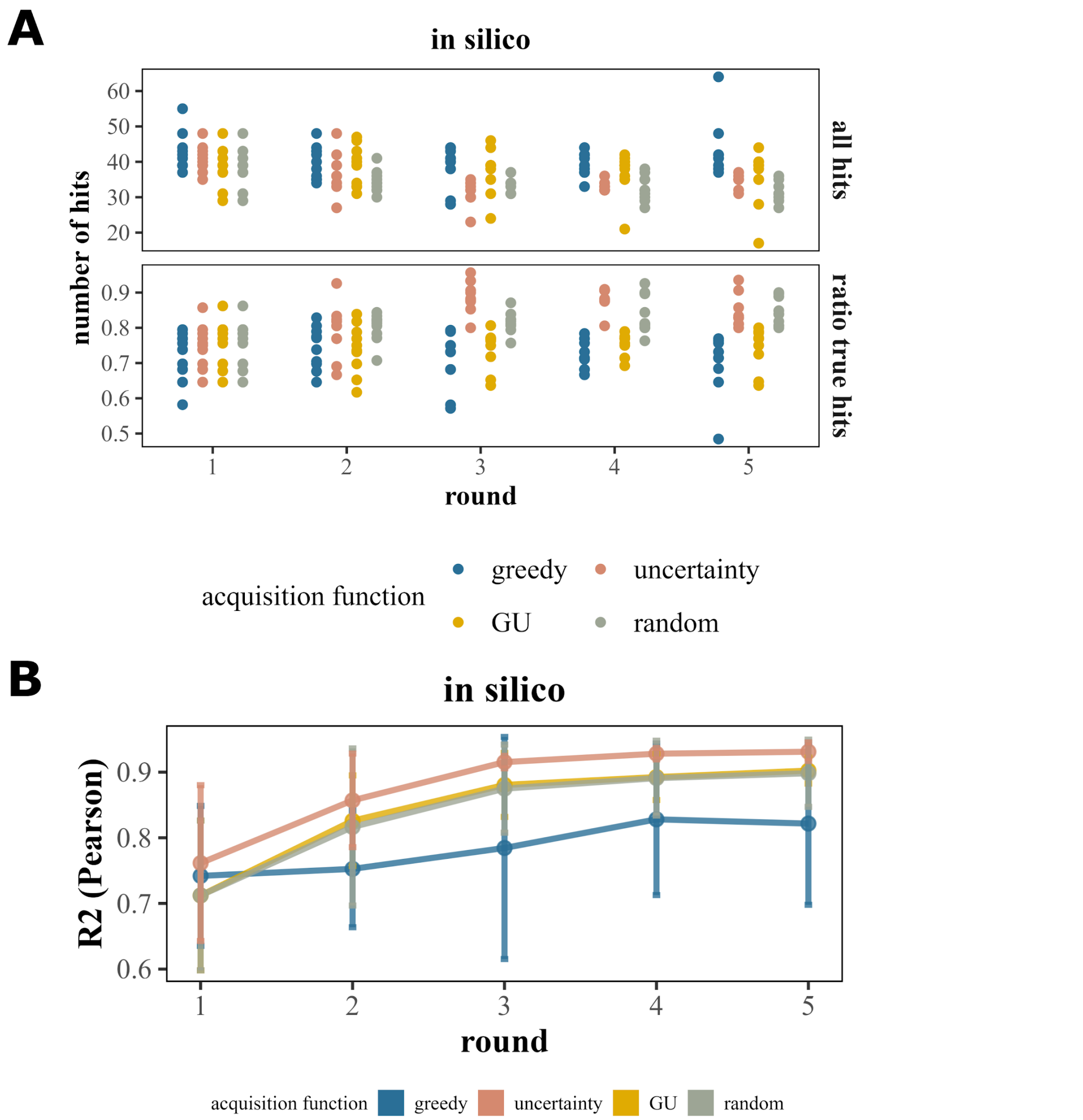

Appendix Figure S7 Effect active learning rounds (A) Number of hits per round, using predictions on all data. The figures are split between visualizing all predicted hits (top, all hits) and the ratio between correct and incorrect hits (bottom, ratio true hits). Each data point represents an optimized model. **(B)** Model performance throughout the rounds, evaluated by comparing model simulations with ground-truth data across both training and unseen datasets. The mean R2 is visualized from an ensemble of ten models of the error on all data, and error bars represent the standard deviation.

Appendix Figure S8 Experimental data of selected conditions

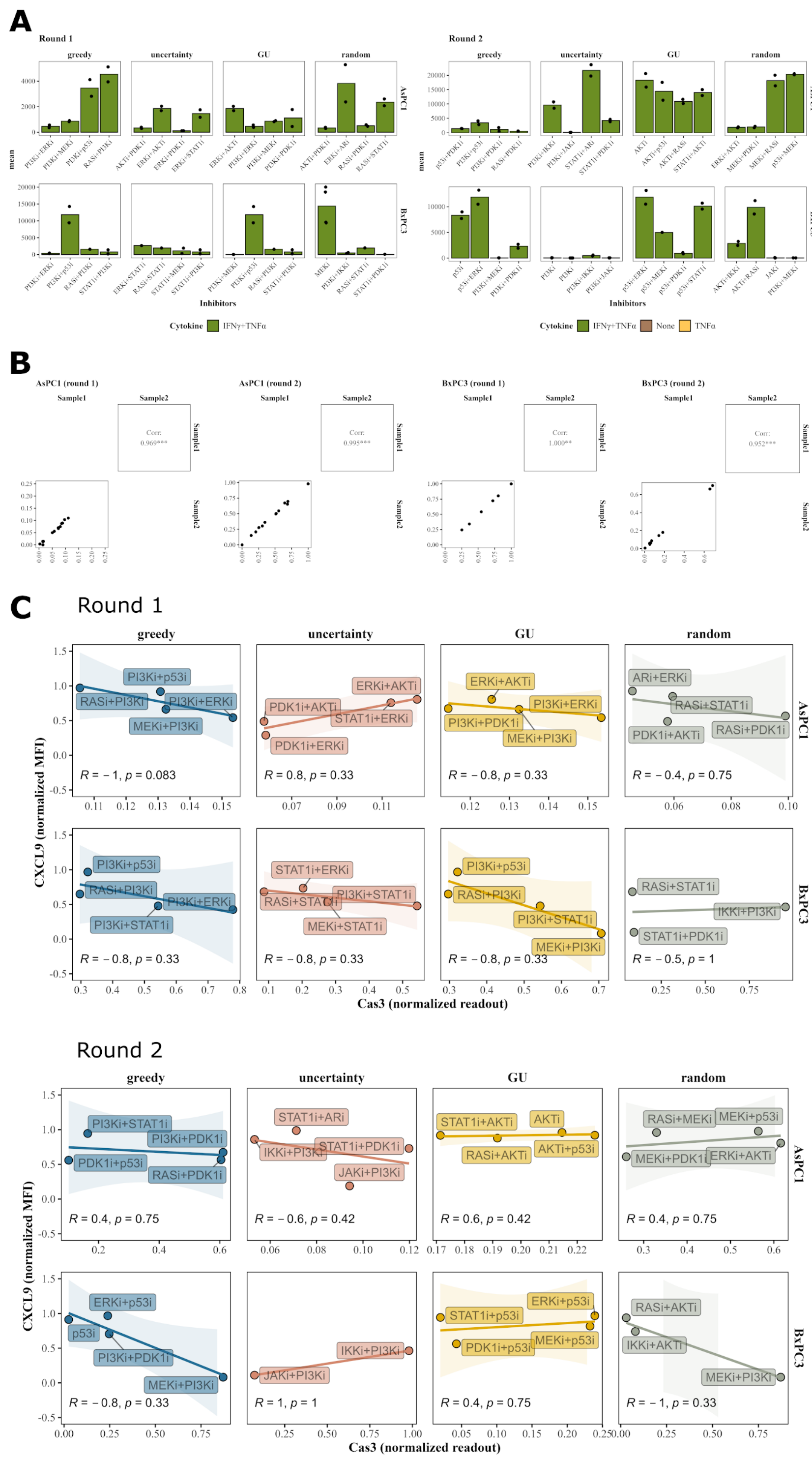

Appendix Figure S8. Experimental data of selected conditions (A) Experimental measurements of the selected conditions per acquisition function for cell lines AsPC1 and BxPC3, across two rounds of active learning. The average is given of two technical replicates, and the error bars represent the standard deviation. **(B)** Correlations between technical replicates over two rounds. Spearman correlations are included to represent the strength of the correlation. **(C)** Caspase-3 and CXCL9 analysis with normalized CXCL9 and Cas3 assay measurements from round 1 (top) and round 2 (bottom). For each acquisition function and cell line, the Spearmon correlation coefficient is calculated. Only conditions with drug combinations are visualized, as they have an increased risk of drug toxicity.

Appendix Table S2 Mean fluorescent intensities AL

Appendix Table S2: Mean fluorescent intensities of AsPC1 and BxPC3 measurements, source data of Figure 5A

cytokine	perturbation	cell line	acquisition function	CXCL9_correction	round
IFNg+TNFa	PI3Ki+ERKi	AsPC1	greedy	0.411153	round 1
IFNg+TNFa	MEKi+PI3Ki	AsPC1	greedy	0.49179	round 1
IFNg+TNFa	RASi+PI3Ki	AsPC1	greedy	0.721598	round 1
IFNg+TNFa	PI3Ki+p53i	AsPC1	greedy	0.699196	round 1
IFNg+TNFa	ERKi+AKTi	AsPC1	uncertainty	0.603348	round 1
IFNg+TNFa	PDK1i+ERKi	AsPC1	uncertainty	0.126308	round 1
IFNg+TNFa	PDK1i+AKTi	AsPC1	uncertainty	0.288259	round 1
IFNg+TNFa	STAT1i+ERKi	AsPC1	uncertainty	0.554489	round 1
IFNg+TNFa	PI3Ki+ERKi	AsPC1	GU	0.411153	round 1
IFNg+TNFa	ERKi+AKTi	AsPC1	GU	0.603348	round 1
IFNg+TNFa	PI3Ki+PDK1i	AsPC1	GU	0.485852	round 1
IFNg+TNFa	MEKi+PI3Ki	AsPC1	GU	0.49179	round 1
IFNg+TNFa	RASi+STAT1i	AsPC1	random	0.584327	round 1
IFNg+TNFa	RASi+PDK1i	AsPC1	random	0.386055	round 1
IFNg+TNFa	ARi+ERKi	AsPC1	random	0.635549	round 1
IFNg+TNFa	PDK1i+AKTi	AsPC1	random	0.288259	round 1
IFNg+TNFa	PI3Ki+p53i	BxPC3	greedy	0.894814	round 1
IFNg+TNFa	RASi+PI3Ki	BxPC3	greedy	0.614941	round 1
IFNg+TNFa	PI3Ki+ERKi	BxPC3	greedy	0.824868	round 1
IFNg+TNFa	PI3Ki+STAT1i	BxPC3	greedy	0.676273	round 1
IFNg+TNFa	PI3Ki+STAT1i	BxPC3	uncertainty	0.676273	round 1
IFNg+TNFa	MEKi+STAT1i	BxPC3	uncertainty	0.503841	round 1
IFNg+TNFa	STAT1i+ERKi	BxPC3	uncertainty	0.607562	round 1
IFNg+TNFa	RASi+STAT1i	BxPC3	uncertainty	0.470331	round 1
IFNg+TNFa	PI3Ki+STAT1i	BxPC3	GU	0.676273	round 1
IFNg+TNFa	RASi+PI3Ki	BxPC3	GU	0.614941	round 1
IFNg+TNFa	PI3Ki+p53i	BxPC3	GU	0.894814	round 1
IFNg+TNFa	MEKi+PI3Ki	BxPC3	GU	0.485697	round 1
IFNg+TNFa	IKKi+PI3Ki	BxPC3	random	1	round 1
IFNg+TNFa	RASi+STAT1i	BxPC3	random	0.470331	round 1
IFNg+TNFa	STAT1i+PDK1i	BxPC3	random	0	round 1
IFNg+TNFa	PI3Ki+PDK1i	AsPC1	greedy	0.810585	round 2
IFNg+TNFa	PI3Ki+STAT1i	AsPC1	greedy	0.681687	round 2
IFNg+TNFa	PDK1i+p53i	AsPC1	greedy	0.351502	round 2
IFNg+TNFa	RASi+PDK1i	AsPC1	greedy	0.726979	round 2
IFNg+TNFa	JAKi+PI3Ki	AsPC1	uncertainty	0.073744	round 2
IFNg+TNFa	IKKi+PI3Ki	AsPC1	uncertainty	0.536352	round 2
IFNg+TNFa	STAT1i+PDK1i	AsPC1	uncertainty	0.490101	round 2

IFNg+TNFa	STAT1i+ARi	AsPC1	uncertainty	0.644138	round 2
IFNg+TNFa	STAT1i+AKTi	AsPC1	GU	0.666896	round 2
IFNg+TNFa	AKTi	AsPC1	GU	0.959644	round 2
IFNg+TNFa	AKTi+p53i	AsPC1	GU	0.708675	round 2
IFNg+TNFa	RASi+AKTi	AsPC1	GU	0.652923	round 2
IFNg+TNFa	MEKi+p53i	AsPC1	random	1	round 2
IFNg+TNFa	MEKi+PDK1i	AsPC1	random	0.505277	round 2
IFNg+TNFa	RASi+MEKi	AsPC1	random	0.813623	round 2
IFNg+TNFa	ERKi+AKTi	AsPC1	random	0.911918	round 2
IFNg+TNFa	MEKi+PI3Ki	BxPC3	greedy	0.566063	round 2
IFNg+TNFa	ERKi+p53i	BxPC3	greedy	0.753348	round 2
IFNg+TNFa	PI3Ki+PDK1i	BxPC3	greedy	0.565905	round 2
IFNg+TNFa	p53i	BxPC3	greedy	0.912502	round 2
TNFa	PI3Ki	BxPC3	uncertainty	0.020725	round 2
IFNg+TNFa	JAKi+PI3Ki	BxPC3	uncertainty	0	round 2
None	PI3Ki	BxPC3	uncertainty	0.127154	round 2
IFNg+TNFa	IKKi+PI3Ki	BxPC3	uncertainty	0.927298	round 2
IFNg+TNFa	ERKi+p53i	BxPC3	GU	0.753348	round 2
IFNg+TNFa	PDK1i+p53i	BxPC3	GU	0.310668	round 2
IFNg+TNFa	STAT1i+p53i	BxPC3	GU	0.574049	round 2
IFNg+TNFa	MEKi+p53i	BxPC3	GU	0.637463	round 2
TNFa	JAKi	BxPC3	random	0.031007	round 2
IFNg+TNFa	RASi+AKTi	BxPC3	random	0.577943	round 2
IFNg+TNFa	IKKi+AKTi	BxPC3	random	0.468785	round 2
IFNg+TNFa	MEKi+PI3Ki	BxPC3	random	0.566063	round 2

Appendix Figure S9 Small PKN

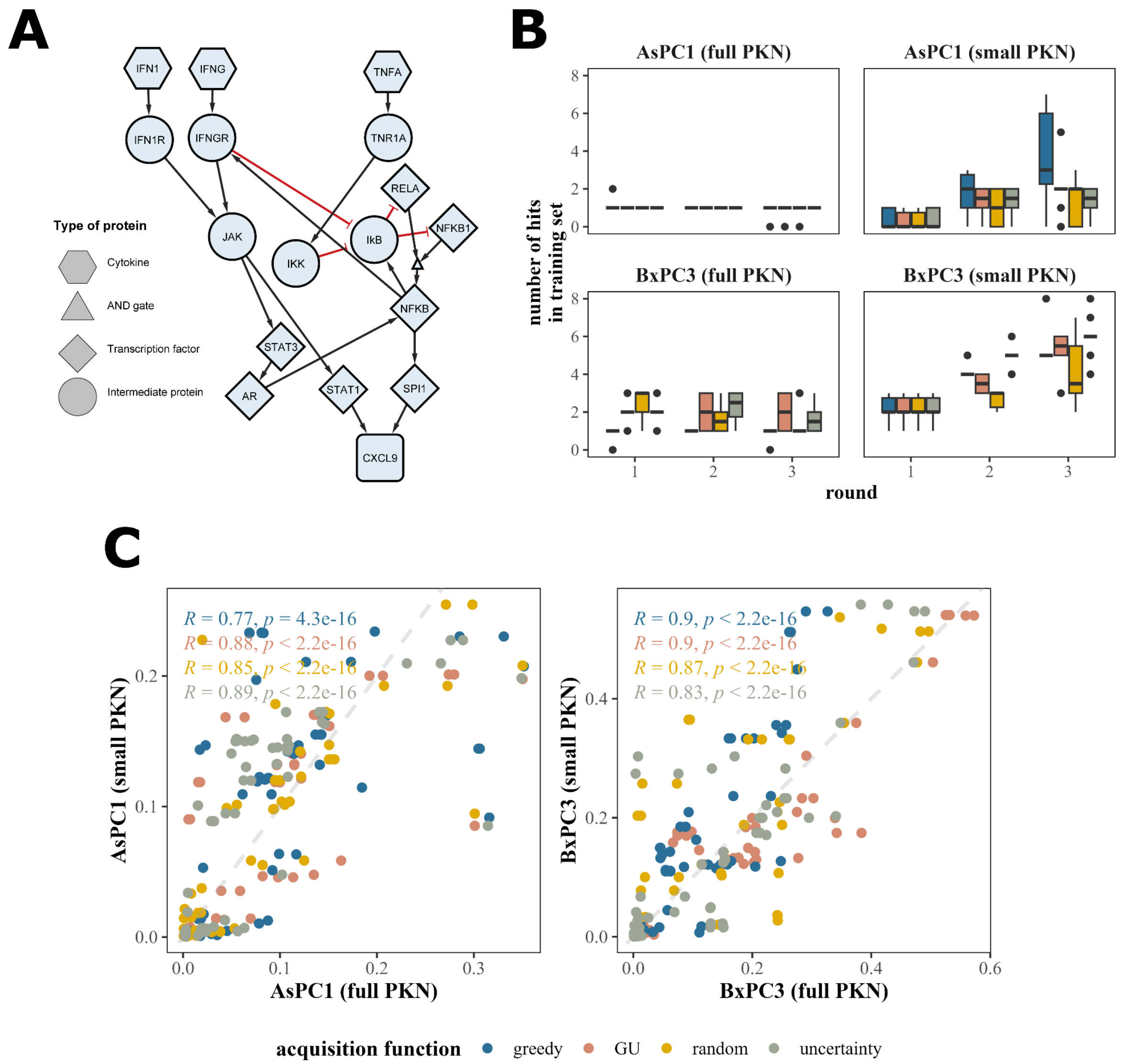

Appendix Figure S9. Small PKN (A) Reduced PKN including only proteins associated with the JAK-STAT and NFKB pathways. **(B)** Comparison of hit discovery across small and full PKN of both cell lines, represented by the number of hits in the training set used to train the model in each round. Results are shown for perturbation conditions within the scope of the reduced PKN (i.e. affecting JAK-STAT and NFKB pathways). Boxplots represents the training sets for ten different models. **(C)** Comparison of the models trained using the small or full PKN. Average simulations from ten models are illustrated, with Spearman correlations for each acquisition function separately. The dotted line represents the y=x line.

Appendix Note - Prior knowledge network

PKN curation

For the prior knowledge network curation, the goal was to construct a cancer signaling network focusing on CXCL9 regulation. We first created a more general PKN focusing on PDL1 and CXCL9/10/11. This curation consisted of the following steps:

1. Determine relevant transcription factors
2. Determine relevant cytokines
3. Link cytokines to transcription factors using intermediate proteins and literature related to major signaling pathways such as JAK-STAT and NFKB

A list of cytokines was retrieved from Cytosig, which contains a database of target genes modulated by cytokines (Jiang *et al*, 2021). The transcription factors (TF) were retrieved from DoRothEA, a database containing a collection of TF and target gene interactions. Each of the transcription factors has a confidence level ranging from A (high) to E (low). This scoring indicates how reliable the found evidence was for the TF-target gene interaction (Garcia-Alonso *et al*, 2019). We only included transcription factors with a confidence level of either A or B. Additionally, the TF should have both a direct (TF binds to gene) and functional (protein levels change) regulation. After determining the cytokines and TF, intermediate nodes and interactions were based on both information from scientific studies related to the immune response of a tumor as well as general knowledge about the signaling pathways JAK-STAT, NFKB, PI3K-AKT, MAPK and p53. Based on the found information, we refined the PKN to focus on CXCL9. The network was iterated upon to filter out less relevant or uncertain nodes.

In the final PKN, we included four cytokines (EGF, IFN1, IFNG and TNFA) as the main stimulators of the MAPK, JAK-STAT and NFKB pathway. Two TF have both a direct and functional regulation: SPI1(Ellis *et al*, 2010) and STAT1 (Tokunaga *et al*, 2018; Sun *et al*, 2018).

For all the details related to the PKN curation, see Supplementary Tables in the Github repository for the literature research we conducted to create the network.

- Supplementary Table 1: Transcription factors of PDL1
- Supplementary Table 2: Transcription factors of CXCL9/10/11
- Supplementary Table 3: Cytokines
- Supplementary Table 4: Interactions (linking cytokines to transcription factors)
- Supplementary Table 5: Adjustments made to create the final CXCL9-specific PKN

Translation to logic-ODE

The CXCL9-specific PKN defines the ODE equations that describe the protein activities within the logic-ODE model. The final version of the PKN used in our study consists of 37 nodes and 61 edges. For the 37 nodes, we had four cytokines (model inputs, EGF, IFN1, IFNG and TNFA) and seven transcription factors (RELA, NFKB1, NFKB, SPI1, STAT1, STAT3, AR) from which two were directly regulating the readout CXCL9 (STAT1, SPI1).

For the parameterization, each protein was described with an ODE with three tunable parameters. We optimized a *k* parameter for each interaction (61 *k* parameters) and a *tau* parameter for each protein that is not an input (33 *tau* parameters). For the JAK-STAT/NFKB-only PKN, the number of optimized parameters was reduced to 20 *k* parameters and 14 *tau* parameters.

Model parameter values

We included average cell line-specific model parameter values of AsPC1 and BxPC3, including original models (trained on first set of perturbation experiments, these models were used for **Figure 2**), and the active learning (AL) models trained with additional *in vitro* experiments, used to generate results of **Figure 5** (models trained for two rounds for each acquisition function). We also added corresponding standard deviations (SD).

Appendix Table S3 Parameter values of original AsPC1 and BxPC3 models

parameter	Mean (AsPC1, original)	SD (AsPC1, original)	Mean (BxPC3, original)	SD (BxPC3, original)
**AKT_k_AR**	0.489055	0.226246	0.420498	0.306074
**AKT_k_IKK**	0.11403	0.123394	0.142129	0.089813
**AKT_k_Mdm2**	9.51E-05	0.000301	0.054483	0.171843
**AR_k_NFKB**	0.229761	0.225897	0.116581	0.147369
**ERK_k_AR**	0.345782	0.274257	0.611926	0.21243
**ERK_k_JUN**	0.193807	0.261739	0.085086	0.176849
**ERK_k_SOS**	0.099165	0.199198	0.169923	0.325938
**ERK_k_p53**	0.017759	0.056056	0.047718	0.150898
**IFN1R_k_JAK**	0.427867	0.197031	0.536863	0.034867
**IFN1R_k_PI3K**	0.268845	0.21977	0.295815	0.222019
**IFN1_k_IFN1R**	2.04E-07	6.45E-07	3.09E-11	6.70E-11
**IFNGR_k_IkB**	0.842312	0.31499	0.462623	0.422311
**IFNGR_k_JAK**	0.245143	0.193722	0.068588	0.073724
**IFNG_k_IFNGR**	2.93E-05	6.73E-05	0.000214	0.000433
**IKK_k_IkB**	0.578868	0.262226	0.738391	0.35805
**IkB_k_NFKB1**	0.201265	0.275071	0.291206	0.340391
**IkB_k_RELA**	0.305534	0.243966	0.274526	0.22524
**JAK_k_PI3K**	0.030107	0.048903	0.035027	0.069814
**JAK_k_STAT1**	0.296092	0.252656	0.053484	0.073118
**JAK_k_STAT3**	0.206435	0.217622	0.248614	0.165544
**JNK_k_JUN**	0.144785	0.308917	0.067788	0.162105
**JUN_k_PTEN**	0.292768	0.360272	0.143376	0.171121
**MEK_k_ERK**	0.502938	0.368252	0.542129	0.347386
**MEK_k_JNK**	0.099385	0.31399	1.10E-08	3.02E-08
**Mdm2_k_p53**	0.110222	0.313862	0.011828	0.037403
**NFKB1_k_NFKB**	0.698732	0.257565	0.567771	0.369096
**NFKB_k_IFNGR**	7.81E-05	0.000245	0.040463	0.099449
**NFKB_k_IkB**	0.11141	0.082119	0.298323	0.145296
**NFKB_k_SPI1**	0.245304	0.195051	0.221999	0.188378
**PDK1_k_AKT**	0.230922	0.203648	0.364759	0.268183
**PDK1_k_IKK**	0.183274	0.165568	0.168366	0.100453
**PI3K_k_AKT**	0.659942	0.346031	0.740311	0.329129
**PI3K_k_PIP3**	0.153716	0.325654	0.182177	0.341896
**PIP3_k_PDK1**	0.207416	0.277253	0.180626	0.225366
**PTEN_k_AKT**	0.617071	0.29398	0.447967	0.357756
**PTEN_k_PIP3**	0.011741	0.034179	0.023008	0.050179
**RAF_k_JUN**	0.198599	0.184586	0.064938	0.14308
**RAF_k_MEK**	0.15773	0.192898	0.226352	0.146883
**RAS_k_PI3K**	0.201879	0.142877	0.047711	0.0807
**RAS_k_RAF**	0.111176	0.279794	0.122532	0.200099
**RELA_k_NFKB**	0.670467	0.362992	0.525494	0.351023
**SOS_k_RAS**	0.13409	0.134172	0.279312	0.452371
**SPI1_k_CXCL9**	0.07178	0.050952	0.031347	0.030931
**STAT1_k_CXCL9**	0.078473	0.13156	0.084027	0.043899
**STAT3_k_AR**	0.161167	0.17251	0.241699	0.290331
**TNFA_k_TNR1A**	1.31E-05	2.88E-05	8.29E-05	0.000245
**TNR1A_k_ERK**	0.020177	0.040863	0.177312	0.23331
**TNR1A_k_IKK**	0.467106	0.121381	0.45585	0.102454

**TNR1A_k_PI3K**	0.004904	0.012429	0.055708	0.086714
**p53_k_Mdm2**	0.000159	0.000502	0.020125	0.061687
**p53_k_PTEN**	0.101198	0.165398	0.055112	0.100981
**tau_AKT**	0.012711	0.006338	0.013877	0.007785
**tau_AR**	0.110575	0.312644	0.015669	0.011338
**tau_CXCL9**	0.366112	0.425981	0.366623	0.425227
**tau_ERK**	0.007989	0.015318	0.004904	0.005659
**tau_IFN1R**	0.003051	0.002742	0.006441	0.004028
**tau_IFNGR**	0.007686	0.003399	0.015523	0.00807
**tau_IKK**	0.572348	0.378858	0.115935	0.188556
**tau_IkB**	0.114904	0.311196	0.064872	0.179008
**tau_JAK**	0.077207	0.121957	0.15587	0.29725
**tau_JNK**	0.000373	0.001129	9.63E-09	3.04E-08
**tau_JUN**	0.005561	0.013557	0.003336	0.006912
**tau_MEK**	0.006621	0.008364	0.00681	0.007394
**tau_Mdm2**	3.35E-06	7.57E-06	3.85E-05	0.000117
**tau_NFKB**	0.102362	0.253438	0.007831	0.006734
**tau_NFKB1**	0.032226	0.068328	0.012394	0.009542
**tau_PDK1**	0.008841	0.017438	0.005217	0.006991
**tau_PI3K**	0.01194	0.011598	0.01065	0.014228
**tau_PIP3**	0.001852	0.005588	0.002687	0.005358
**tau_PTEN**	0.00407	0.00593	0.012156	0.028502
**tau_RAF**	0.000938	0.001905	0.001373	0.00162
**tau_RAS**	0.002195	0.00207	0.007071	0.015353
**tau_RELA**	0.018056	0.017496	0.017655	0.013282
**tau_SOS**	3.61E-05	7.79E-05	0.001225	0.003736
**tau_SPI1**	0.089908	0.214949	0.00997	0.005044
**tau_STAT1**	0.00152	0.001545	0.001155	0.000534
**tau_STAT3**	0.012328	0.00713	0.085617	0.242533
**tau_TNR1A**	0.004124	0.002067	0.004671	0.003094
**tau_p53**	0.000592	0.001319	0.000736	0.002327

Appendix Table S4 Parameter values of AsPC1 active learning models

parameter	Mean (AsPC1, greedy)	SD (AsPC1, greedy)	Mean (AsPC1, uncertainty)	SD (AsPC1, uncertainty)	Mean (AsPC1, GU)	SD (AsPC1, GU)	Mean (AsPC1, random)	SD (AsPC1, random)
**AKT_k_AR**	0.556933	0.322888	0.462473	0.212152	0.758215	0.316791	0.282276	0.20981
**AKT_k_IKK**	0.217677	0.155498	0.316191	0.234458	0.487807	0.329226	0.303498	0.257411
**AKT_k_Mdm2**	0.016741	0.039564	1.35E-11	4.27E-11	0.000249	0.000774	0.003943	0.012297
**AR_k_NFKB**	0.16975	0.191834	0.329771	0.292469	0.242052	0.275029	0.468095	0.301535
**ERK_k_AR**	0.19395	0.242533	0.362687	0.224098	0.321535	0.381143	0.341999	0.388448
**ERK_k_JUN**	0.266081	0.25756	0.187327	0.319739	0.162813	0.220737	0.40936	0.381025
**ERK_k_SOS**	0.027138	0.081498	0	0	0.000139	0.000312	0.190211	0.391234
**ERK_k_p53**	0.011307	0.022587	2.65E-05	5.70E-05	0.000441	0.000904	0.000178	0.000557
**IFN1R_k_JAK**	0.542229	0.115758	0.485437	0.114153	0.443253	0.142163	0.511562	0.352953
**IFN1R_k_PI3K**	0.309669	0.200388	0.368049	0.32447	0.161841	0.179751	0.360407	0.321404
**IFN1_k_IFN1R**	3.39E-08	1.07E-07	0	0	1.78E-09	5.53E-09	2.50E-09	5.30E-09
**IFNGR_k_IkB**	0.787001	0.372	0.737666	0.366874	0.624465	0.394913	0.646776	0.382028
**IFNGR_k_JAK**	0.094702	0.112301	0.094563	0.118333	0.204451	0.210373	0.406074	0.378644
**IFNG_k_IFNGR**	2.52E-05	7.29E-05	0.000731	0.002054	1.39E-05	1.35E-05	0.008623	0.026993
**IKK_k_IkB**	0.587375	0.312866	0.605743	0.297308	0.465028	0.184945	0.366775	0.230984
**IkB_k_NFKB1**	0.260989	0.281649	0.25866	0.188402	0.152105	0.183989	0.228383	0.320089
**IkB_k_RELA**	0.451039	0.333485	0.406551	0.299638	0.279189	0.25098	0.243592	0.216164
**JAK_k_PI3K**	0.078258	0.136928	0.028656	0.054913	0.026875	0.069048	0.142468	0.090312
**JAK_k_STAT1**	0.168586	0.188155	0.025484	0.042382	0.189658	0.31061	0.083676	0.113944
**JAK_k_STAT3**	0.217996	0.19073	0.295453	0.205671	0.264875	0.189301	0.379977	0.319529
**JNK_k_JUN**	0.190795	0.238252	0.183368	0.291729	0.125604	0.187971	0.092335	0.126894
**JUN_k_PTEN**	0.316353	0.384415	0.362214	0.190592	0.220152	0.231388	0.369148	0.371798
**MEK_k_ERK**	0.647568	0.413305	0.531196	0.236883	0.613295	0.374036	0.301349	0.264128
**MEK_k_JNK**	0.197629	0.3227	0.098222	0.27473	0.055765	0.120936	0.126843	0.206161
**Mdm2_k_p53**	0.155629	0.202322	0.034656	0.109593	0.146438	0.18252	0.011683	0.033308
**NFKB1_k_NFKB**	0.513934	0.377163	0.51113	0.313252	0.593678	0.353306	0.604984	0.374277
**NFKB_k_IFNGR**	0.006641	0.016317	0.044584	0.113894	0.000215	0.00046	0.006538	0.015723
**NFKB_k_IkB**	0.155729	0.163914	0.100894	0.082756	0.117192	0.105769	0.045961	0.056067
**NFKB_k_SPI1**	0.292984	0.253511	0.268526	0.188868	0.226453	0.182298	0.251162	0.146463
**PDK1_k_AKT**	0.482473	0.344009	0.04385	0.067455	0.031872	0.03877	0.194246	0.137114
**PDK1_k_IKK**	0.283425	0.135824	0.363619	0.287415	0.28977	0.234991	0.206838	0.162535
**PI3K_k_AKT**	0.553135	0.316257	0.649087	0.126362	0.514438	0.290836	0.65607	0.188322
**PI3K_k_PIP3**	0.090391	0.2758	0.198516	0.375686	0.210502	0.390542	0.311455	0.415991
**PIP3_k_PDK1**	0.214239	0.267964	0.420916	0.366969	0.319446	0.414379	0.309902	0.425544
**PTEN_k_AKT**	0.546567	0.27478	0.773491	0.267633	0.834016	0.206956	0.387396	0.32069
**PTEN_k_PIP3**	0.010843	0.032308	0.056088	0.109214	0.048651	0.112663	0.031985	0.042329
**RAF_k_JUN**	0.096298	0.133348	0.200654	0.220022	0.070713	0.096227	0.172232	0.333499
**RAF_k_MEK**	0.458787	0.49669	0.343428	0.288704	0.143637	0.239077	0.113531	0.313815
**RAS_k_PI3K**	0.117524	0.145979	0.242434	0.134975	0.181574	0.176575	0.221684	0.159849
**RAS_k_RAF**	0.193765	0.297277	0.239359	0.355513	0.126661	0.290521	0.049165	0.077707
**RELA_k_NFKB**	0.638011	0.259953	0.681784	0.325813	0.647614	0.146943	0.574194	0.329467
**SOS_k_RAS**	0.141901	0.21263	0.118271	0.146908	0.119372	0.303811	0.161824	0.16453
**SPI1_k_CXCL9**	0.061953	0.033624	0.103417	0.080466	0.074782	0.07142	0.169656	0.103635
**STAT1_k_CXCL9**	0.10244	0.065197	0.39783	0.193454	0.107098	0.086426	0.399699	0.171444
**STAT3_k_AR**	0.197027	0.272313	0.256031	0.254934	0.243414	0.274727	0.077568	0.099682
**TNFA_k_TNR1A**	1.56E-05	4.09E-05	9.36E-05	0.00016	2.68E-05	4.50E-05	0.000194	0.0004
**TNR1A_k_ERK**	0.295189	0.329925	0.061774	0.11618	0.208473	0.361173	0.060536	0.099527
**TNR1A_k_IKK**	0.278464	0.194114	0.198874	0.163135	0.264728	0.245877	0.300412	0.165533

**TNR1A_k_PI3K**	0.002654	0.005985	0.024679	0.07324	0.000248	0.00043	0.076875	0.082619
**p53_k_Mdm2**	0.002312	0.004986	1.35E-11	4.27E-11	0.001092	0.003452	7.98E-08	2.52E-07
**p53_k_PTEN**	0.110726	0.139465	0.083038	0.122208	0.088261	0.124865	0.133945	0.197741
**tau_AKT**	0.018133	0.013619	0.02654	0.018009	0.137355	0.302709	0.013758	0.016419
**tau_AR**	0.011861	0.012507	0.019976	0.019365	0.007995	0.007085	0.042861	0.100772
**tau_CXCL9**	0.150603	0.114947	0.121675	0.295342	0.226724	0.307757	0.064986	0.126828
**tau_ERK**	0.007242	0.011516	0.024819	0.028237	0.026605	0.036092	0.013379	0.012746
**tau_IFN1R**	0.005	0.00432	0.00734	0.005598	0.00424	0.004955	0.004268	0.003058
**tau_IFNGR**	0.012336	0.009221	0.010334	0.005377	0.011503	0.004773	0.008265	0.004072
**tau_IKK**	0.095813	0.143301	0.018273	0.014323	0.204028	0.378134	0.142158	0.310825
**tau_IkB**	0.040566	0.054816	0.011948	0.008003	0.110644	0.312508	0.071685	0.190704
**tau_JAK**	0.069111	0.132534	0.12307	0.311077	0.068417	0.128947	0.103152	0.279213
**tau_JNK**	0.002582	0.004374	0.002643	0.007199	0.000595	0.001252	0.002213	0.003923
**tau_JUN**	0.039026	0.098219	0.006727	0.007687	0.090953	0.225111	0.011964	0.024041
**tau_MEK**	0.007456	0.012227	0.006311	0.002618	0.004903	0.005493	0.004161	0.005231
**tau_Mdm2**	0.001313	0.003169	1.35E-11	4.27E-11	9.76E-05	0.000266	0.000259	0.00082
**tau_NFKB**	0.018644	0.014927	0.02112	0.007269	0.01472	0.009553	0.024295	0.026574
**tau_NFKB1**	0.20028	0.397563	0.008958	0.006202	0.00821	0.0049	0.016503	0.026033
**tau_PDK1**	0.004184	0.004098	0.032244	0.05253	0.042704	0.116161	0.005529	0.007426
**tau_PI3K**	0.166902	0.341485	0.132784	0.307213	0.208845	0.413098	0.14422	0.313892
**tau_PIP3**	0.001201	0.003006	0.004251	0.010053	0.010787	0.028859	0.005151	0.007442
**tau_PTEN**	0.009854	0.007349	0.06316	0.113401	0.023615	0.042487	0.011028	0.008626
**tau_RAF**	0.000961	0.002074	0.066755	0.205552	0.000367	0.00096	0.001538	0.003482
**tau_RAS**	0.002914	0.004602	0.004308	0.003029	0.003451	0.003322	0.003421	0.002882
**tau_RELA**	0.057781	0.151286	0.00732	0.004753	0.033091	0.060864	0.013821	0.016396
**tau_SOS**	8.46E-05	0.00018	0	0	0.000102	0.000242	0.000554	0.001038
**tau_SPI1**	0.00877	0.009937	0.080461	0.207891	0.10505	0.313225	0.010847	0.011362
**tau_STAT1**	0.000543	0.000373	0.004309	0.002431	0.001613	0.002341	0.00495	0.004154
**tau_STAT3**	0.011386	0.005763	0.019423	0.015195	0.013393	0.012888	0.009754	0.016569
**tau_TNR1A**	0.009085	0.007917	0.003806	0.00306	0.005937	0.005182	0.005322	0.005907
**tau_p53**	0.008135	0.009099	0.000735	0.001746	0.010221	0.022299	0.004184	0.008537

Appendix Table S5 Parameter values of BxPC3 active learning models

parameter	Mean (BxPC3, greedy)	SD (BxPC3, greedy)	Mean (BxPC3, uncertainty)	SD (BxPC3, uncertainty)	Mean (BxPC3, GU)	SD (BxPC3, GU)	Mean (BxPC3, random)	SD (BxPC3, random)
**AKT_k_AR**	0.36295	0.23951	0.357163	0.22097	0.385551	0.251341	0.588554	0.319476
**AKT_k_IKK**	0.210196	0.154899	0.241995	0.172796	0.373668	0.302299	0.042011	0.115155
**AKT_k_Mdm2**	0.111781	0.31413	0.138581	0.325712	3.60E-07	1.13E-06	0.000165	0.000519
**AR_k_NFKB**	0.188244	0.191635	0.225692	0.175662	0.131998	0.094053	0.11159	0.131662
**ERK_k_AR**	0.578118	0.268353	0.539664	0.298701	0.291834	0.241181	0.387149	0.37463
**ERK_k_JUN**	0.311882	0.349008	0.188516	0.255307	0.264541	0.343599	0.129954	0.176505
**ERK_k_SOS**	0.030557	0.062017	0.080005	0.193424	0.00753	0.023811	0.039229	0.122895
**ERK_k_p53**	0.008661	0.027267	0.034164	0.108035	0.041266	0.129032	0.101693	0.31492
**IFN1R_k_JAK**	0.580571	0.176937	0.502156	0.087742	0.549309	0.189276	0.463517	0.182057
**IFN1R_k_PI3K**	0.364302	0.21712	0.380669	0.224953	0.371409	0.295977	0.427156	0.258695
**IFN1_k_IFN1R**	1.54E-11	4.89E-11	5.79E-10	1.62E-09	1.50E-09	4.60E-09	1.93E-09	5.94E-09
**IFNGR_k_IkB**	0.874819	0.162127	0.540158	0.413457	0.595953	0.323314	0.668701	0.39714
**IFNGR_k_JAK**	0.250203	0.349671	0.1678	0.184055	0.121267	0.157364	0.184062	0.218888
**IFNG_k_IFNGR**	3.53E-06	7.15E-06	0.001367	0.004319	0.000571	0.001283	0.003389	0.010284
**IKK_k_IkB**	0.741558	0.275966	0.53184	0.336476	0.597808	0.345044	0.508389	0.36138
**IkB_k_NFKB1**	0.378813	0.214126	0.422934	0.239855	0.254826	0.256556	0.184316	0.23137
**IkB_k_RELA**	0.269856	0.244909	0.39156	0.188663	0.284766	0.233311	0.337306	0.267817
**JAK_k_PI3K**	0.083604	0.116933	0.051273	0.119029	0.049086	0.066571	0.002599	0.004149
**JAK_k_STAT1**	0.179868	0.181416	0.184978	0.177843	0.068713	0.143894	0.274807	0.195795
**JAK_k_STAT3**	0.330423	0.220685	0.415255	0.296811	0.272451	0.208772	0.172549	0.203869
**JNK_k_JUN**	0.339074	0.349392	0.104623	0.227289	0.344667	0.324019	0.267644	0.233184
**JUN_k_PTEN**	0.555402	0.313858	0.304504	0.265316	0.461706	0.34187	0.357397	0.303513
**MEK_k_ERK**	0.190887	0.105426	0.341142	0.139029	0.179325	0.295524	0.508815	0.348977
**MEK_k_JNK**	0.007977	0.024931	0.057109	0.180593	0.129534	0.278007	0.228553	0.415378
**Mdm2_k_p53**	0.208017	0.188292	0.059258	0.14343	0.180089	0.218628	0.052853	0.163536
**NFKB1_k_NFKB**	0.724854	0.276272	0.396556	0.364513	0.414496	0.460652	0.38363	0.372859
**NFKB_k_IFNGR**	0.011712	0.037038	0.006727	0.021271	0.030805	0.070573	0.001924	0.005961
**NFKB_k_IkB**	0.25889	0.197494	0.173147	0.239177	0.13868	0.114138	0.138916	0.203496
**NFKB_k_SPI1**	0.292117	0.18764	0.232815	0.173744	0.270914	0.18501	0.170604	0.15137
**PDK1_k_AKT**	0.122131	0.134324	0.157413	0.164077	0.177053	0.159997	0.33403	0.284457
**PDK1_k_IKK**	0.107374	0.095406	0.086163	0.104139	0.19095	0.152211	0.195447	0.146802
**PI3K_k_AKT**	0.505993	0.334352	0.773917	0.287218	0.60296	0.360217	0.641673	0.35316
**PI3K_k_PIP3**	0.188114	0.379239	0.04637	0.146634	0.11178	0.25789	0.106654	0.314033
**PIP3_k_PDK1**	0.2774	0.317968	0.199267	0.337886	0.252474	0.277856	0.739637	0.412138
**PTEN_k_AKT**	0.488595	0.314911	0.590852	0.374522	0.445349	0.38582	0.612291	0.318534
**PTEN_k_PIP3**	0.010725	0.022792	1.49E-05	4.71E-05	0.015937	0.04469	0.146557	0.30323
**RAF_k_JUN**	0.288076	0.307681	0.089294	0.189863	0.356693	0.302053	0.163703	0.141744
**RAF_k_MEK**	0.371003	0.422677	0.19847	0.148143	0.186045	0.39067	0.126764	0.160218

**RAS_k_PI3K**	0.087801	0.132054	0.135929	0.139498	0.049718	0.069709	0.083602	0.114737
**RAS_k_RAF**	0.234205	0.263813	0.21145	0.335401	0.074034	0.165496	0.350927	0.457331
**RELA_k_NFKB**	0.519079	0.277088	0.55426	0.357065	0.379214	0.420396	0.391707	0.407457
**SOS_k_RAS**	0.5494	0.407827	0.132167	0.312137	0.110728	0.258143	0.113622	0.206785
**SPI1_k_CXCL9**	0.089471	0.094259	0.073625	0.064742	0.106854	0.11089	0.071518	0.050211
**STAT1_k_CXCL9**	0.08609	0.06624	0.121595	0.178154	0.101806	0.095093	0.140601	0.110731
**STAT3_k_AR**	0.373638	0.192758	0.114867	0.159733	0.193806	0.196516	0.223472	0.343855
**TNFA_k_TNR1A**	6.76E-06	1.31E-05	2.23E-06	4.18E-06	0.00011	0.000232	0.000454	0.001425
**TNR1A_k_ERK**	0.039992	0.069385	0.16119	0.326044	0.157069	0.330797	0.088384	0.09915
**TNR1A_k_IKK**	0.385351	0.162158	0.382485	0.227744	0.243124	0.181438	0.135134	0.172131
**TNR1A_k_PI3K**	0.010258	0.015941	0.006034	0.015706	0.052288	0.095869	0.06116	0.110644
**p53_k_Mdm2**	0.125466	0.311629	0.1114	0.314271	0.022056	0.060946	0.003224	0.010196
**p53_k_PTEN**	0.251223	0.146058	0.150382	0.267798	0.207515	0.164945	0.231097	0.313467
**tau_AKT**	0.011767	0.01067	0.00866	0.005125	0.013671	0.009234	0.01289	0.010901
**tau_AR**	0.010064	0.004454	0.008175	0.005176	0.060665	0.072654	0.021497	0.029992
**tau_CXCL9**	0.05662	0.06024	0.057144	0.044734	0.044282	0.054045	0.099963	0.097346
**tau_ERK**	0.003074	0.001614	0.00615	0.004905	0.003662	0.006829	0.015686	0.026093
**tau_IFN1R**	0.002378	0.001343	0.00388	0.001397	0.004436	0.003252	0.005036	0.004386
**tau_IFNGR**	0.008708	0.004488	0.00924	0.004019	0.009215	0.004202	0.009854	0.006262
**tau_IKK**	0.248705	0.401047	0.142768	0.306623	0.115316	0.252312	0.09783	0.283721
**tau_IkB**	0.101162	0.279058	0.019742	0.028145	0.091329	0.142226	0.100577	0.224317
**tau_JAK**	0.010418	0.007836	0.155898	0.283916	0.060481	0.077434	0.248629	0.352237
**tau_JNK**	3.97E-06	8.77E-06	0.000959	0.003033	0.001102	0.002315	0.002645	0.00451
**tau_JUN**	0.003184	0.005305	0.004777	0.007974	0.02577	0.0541	0.083718	0.257864
**tau_MEK**	0.001937	0.003228	0.002892	0.001808	0.003486	0.007076	0.006969	0.00601
**tau_Mdm2**	0.00067	0.001266	4.63E-05	0.000139	0.000725	0.002018	3.06E-05	9.67E-05
**tau_NFKB**	0.010392	0.007789	0.070888	0.199698	0.102123	0.296892	0.017475	0.021203
**tau_NFKB1**	0.120419	0.30397	0.012656	0.012756	0.028405	0.054781	0.096089	0.280585
**tau_PDK1**	0.004058	0.005864	0.0253	0.076645	0.001953	0.003842	0.010931	0.010767
**tau_PI3K**	0.015757	0.020889	0.063404	0.15178	0.006266	0.00462	0.073104	0.19321
**tau_PIP3**	0.002485	0.005419	0.001086	0.003434	0.001825	0.005006	0.000264	0.000539
**tau_PTEN**	0.085563	0.20182	0.009388	0.016795	0.103961	0.261074	0.049729	0.141655
**tau_RAF**	0.013659	0.020021	0.009714	0.02957	0.00951	0.02571	0.002972	0.003804
**tau_RAS**	0.005201	0.006117	0.001273	0.001794	0.001188	0.003201	0.002554	0.003787
**tau_RELA**	0.039996	0.100246	0.163745	0.328534	0.027798	0.035059	0.10714	0.289442
**tau_SOS**	0.000271	0.000545	3.38E-05	6.88E-05	0.000977	0.003067	8.76E-05	0.000182
**tau_SPI1**	0.006264	0.007697	0.008648	0.007426	0.011904	0.010964	0.008405	0.010372
**tau_STAT1**	0.001148	0.000865	0.001816	0.001468	0.000723	0.000793	0.040001	0.119123
**tau_STAT3**	0.012261	0.006477	0.011371	0.010375	0.01543	0.010849	0.008358	0.00918
**tau_TNR1A**	0.002865	0.001183	0.002023	0.001787	0.003718	0.002023	0.004164	0.003802
**tau_p53**	0.008132	0.008423	0.00034	0.000844	0.006513	0.005607	0.001712	0.003018

Appendix Note - Apoptosis correction

An issue of testing inhibitor combinations is the increased risk of drug toxicity, which could affect cell viability and apoptosis. We decided to investigate apoptosis because we observed that conditions sampled by greedy or GU sometimes had visibly lower CXCL9 activities compared to conditions selected by for instance uncertainty (**Appendix Figure S8A**). Changes in cell counts can in turn affect CXCL9 secretion. A limitation of the current PKN is that we focus on CXCL9 regulation and have not included pathways such as apoptosis or stress response. Therefore, if a clear negative correlation is present between CXCL9 secretion and apoptosis, suggesting that induced apoptosis affected CXCL9 secretion, our logic-ODE will not take apoptosis into account. What would be possible however is to integrate the knowledge of this possibly confounding factor in the data preparation for the model.

Therefore, we first quantified the effect of apoptosis on CXCL9. The results were used to decide whether we would need to take apoptosis into account via e.g. correction of normalized CXCL9 values. As strong negative correlations could be found (**Appendix Figure S8C**), especially for sampling strategies focusing on high CXCL9 values (i.e. greedy, GU), we continued with a correction for the impact of apoptosis on CXCL9 values. Our aim was to ensure that we could recover trends that focus on CXCL9 effects, i.e. drug combinations that result in the highest CXCL9. If a side effect of this combination is apoptosis, we would like to diminish the impact on the CXCL9 trends so we can focus on CXCL9 regulation as our model does not include apoptosis. In other words, based on the correlation between CXCL9 and apoptosis, we made an assumption that a high apoptosis overpowered CXCL9 secretion. So conditions with a high apoptosis require the biggest correction (increase in CXCL9), which is possible through summing normalized CXCL9 and apoptosis values, before rescaling the resulting activities to a range of 0-1. This correction allowed us to recover more distinct trends across acquisition functions.

Appendix References

Ellis SL, Gysbers V, Manders PM, Li W, Hofer MJ, Müller M & Campbell IL (2010) The cell-specific induction of CXC chemokine ligand 9 mediated by IFN-gamma in microglia of the central nervous system is determined by the myeloid transcription factor PU.1. *J Immunol* 185: 1864–1877

Garcia-Alonso L, Holland CH, Ibrahim MM, Turei D & Saez-Rodriguez J (2019) Benchmark and integration of resources for the estimation of human transcription factor activities. *Genome Res* 29: 1363–1375

Jiang P, Zhang Y, Ru B, Yang Y, Vu T, Paul R, Mirza A, Altan-Bonnet G, Liu L, Ruppin E, *et al* (2021) Systematic investigation of cytokine signaling activity at the tissue and single-cell levels. *Nat Methods* 18: 1181–1191

Schubert M, Klinger B, Klünemann M, Sieber A, Uhlitz F, Sauer S, Garnett MJ, Blüthgen N & Saez-Rodriguez J (2018) Perturbation-response genes reveal signaling footprints in cancer gene expression. *Nat Commun* 9: 20

Sun C, Mezzadra R & Schumacher TN (2018) Regulation and function of the PD-L1 checkpoint. *Immunity* 48: 434–452

Tokunaga R, Zhang W, Naseem M, Puccini A, Berger MD, Soni S, McSkane M, Baba H & Lenz H-J (2018) CXCL9, CXCL10, CXCL11/CXCR3 axis for immune activation - A target for novel cancer therapy. *Cancer Treat Rev* 63: 40–47
