# Supplementary material for: Active learning-guided mechanistic modeling reveals context-specific regulators of CXCL9 expression in pancreatic cancer cells
Source: Mol Syst Biol. 2026 Jun 4;22(8):1360–75. doi: 10.1038/s44320-026-00221-w (PMC13434641; doi:10.1038/s44320-026-00221-w)
Supplement: Supplementary file 2 [file 44320_2026_221_MOESM2_ESM.pdf]

molecular systems biology

Active learning-guided mechanistic modeling of CXCL9 regulation in pancreatic cancer

Bi-rong Wang, Maaruthy Yelleswarapu, Lucie Descamps, and Federica Eduati

Corresponding author(s): Federica Eduati (f.eduati@tue.nl)

Review Timeline:

Submission Date:	24th Oct 25
Editorial Decision:	9th Dec 25
Appeal Received:	12th Dec 25
Editorial Decision:	19th Dec 25
Revision Received:	19th Mar 26
Editorial Decision:	28th Apr 26
Revision Received:	22nd May 26
Accepted:	27th May 26

Editor: Jingyi Hou

Transaction Report:

(Note: With the exception of the correction of typographical or spelling errors that could be a source of ambiguity, letters and reports are not edited. Depending on transfer agreements, referee reports obtained elsewhere may or may not be included in this compilation. Referee reports are anonymous unless the Referee chooses to sign their reports.)

9th Dec 2025
RE: Manuscript MSB-2025-13425, Active learning-guided mechanistic modeling of CXCL9 regulation in pancreatic cancer

Dear Dr Eduati,

Thank you for submitting your work to Molecular Systems Biology. We have now heard back from three reviewers who agreed to evaluate your manuscript. As you will see from their comments below, the reviewers raise substantial concerns about your study, which unfortunately preclude its publication in Molecular Systems Biology.

While Reviewer #3 is generally supportive, Reviewer #1 expressed significant concerns regarding both the overall computational methodology and the resulting biological insights. Reviewer #2 provided several positive remarks but also identified important methodological limitations. Notably, they expressed concern that the scope of the study is limited to CXCL9, which aligns with our editorial perspective that the work in its current form does not sufficiently demonstrate the broader applicability of the proposed modeling approach or yield more broadly relevant biological insights.

In view of the reviewers' comments and our editorial evaluation, we regret to inform you that we are unable to offer publication of your manuscript.

While we cannot pursue this manuscript further, we encourage you to transfer your study to our not-for-profit open-access sister journal, Life Science Alliance (LSA). We shared your manuscript and the accompanying reviews with LSA Executive Editor, Tim Fessenden, who is interested in these findings. He is pleased to offer consideration of this manuscript at LSA pending the following revisions:

-We encourage you to address the point of Reviewer 1 on the analyses being circular, and the lack of additional data to train the model. In the least, the discussion must elaborate on these concerns.
-Further you must elaborate on the technical concerns raised by all the reviewers (rev 1, major point 2, all of rev2's points, and rev 3, ref line 173).
-Finally, you must include all the additional details and description of results requested by the reviewer 3 and reviewer 1, major point 3.

We understand that such a revision might need to be re-reviewed, in which case Dr. Fessenden will walk the Reviewers through our transfer process.

We encourage you to contact Dr. Fessenden at t.fessenden@life-science-alliance.org to discuss this work and the revisions requested. You may use the link below to immediately transfer your manuscript to LSA. You do not need to revise the manuscript before transferring it to LSA. Once you transfer, you will receive an invitation to revise and resubmit. Again please contact Dr. Fessenden if you have any questions about the LSA journal, the transfer process, or the revisions requested.

I am very sorry that the review of your work did not result in a more favorable outcome on this occasion, but I hope you will not be discouraged from sending your work to Molecular Systems Biology in the future. In any case, thank you for the opportunity to examine this work.

Sincerely,
Jingyi

Jingyi Hou, PhD
Senior Editor
Molecular Systems Biology

Reviewer #1:

In their manuscript entitled „Active learning-guided mechanistic modeling of CXCL9 regulation in pancreatic cancer", Eduerti and colleagues describe the development of a logic-ODE model to describe the signal-dependent expression of CXCL9 in two pancreatic cancer cell lines. They train the model based on a perturbation data set where they inhibit some pathways with small molecule inhibitors and/or stimulate cells with TNFa, IFNg or both. They then use the Model to simulate (combinatorial) knockouts and found combinations that led to synergy for the induction between TNFa and IFNg. These simulation results were

not tested experimentally.
Subsequently, they implemented an active learning pipeline to learn optimal "experiments" to better refine the model. Briefly, they used an ensemble of models optimized to the original data set, then used this to sample conditions that either maximise CXCL9 induction, or that show maximal uncertainty, or a mixture of both or random. Then they simulated data from one model to refine the parameterization, and iterated these. They then describe which strategies lead to optimized CXCL9 production or better accuracy of the model. For instance, they show that a mixture of greedy and uncertainty performs best when maximizing CXCL9. The authors then perform selected experiments that somewhat cover perturbations that were selected during the training. They then show that these experiments correlate with their findings of the in silico training.
Overall I think the manuscript could nicely illustrate the combination of modelling and active learning for a technical audience. However, the current manuscript has some methodological shortcomings and is not conclusive (both computationally and biologically) to meet the interest for a broader audience of MSB. I am not sure what exactly I learn biologically or methodologically, given the lack of biological testing or deep methodological description.
Major comments:
- The way the authors perform the analysis is circular - they train the model on a model trained by the same data. This training will never be able to perform better then the original model. Thus the trained models can also not be "more" predictive then that model. Why they did not use additional data on the predicted optimal perturbations to retrain the models is unclear to me.
- The fact that the CXCL9 protein levels are higher in the greedy strategy is not surprising, this is what the conditions are optimized for. Why not validating the model with the condition that maximizes CXCL9 expression in the final model pool *after* training? That would show that how the model training actually performs.
- I find the presentation at many parts very superficial. It would be very important in my eyes which exact conditions were tested in the data of Fig. 5. Were these all conditions? Or selected ones? How does the data actually look like? Also, I would have appreciated a clearer description of the PKN (how exactly does the model look like?

Reviewer #2:

Very well written paper. An interesting, presented tool to drive hypothesis building. Some issues which are listed below, but do not detract from the conceptual advancement and impact on this field. The format of my review follows the editor's requested points.
1. Understanding of the Story
The manuscript addresses the challenge of poor immune infiltration in pancreatic cancer (PDAC) by focusing on CXCL9, a chemokine that recruits $CD8^+$ T cells. The authors propose an integrated experimental-computational pipeline combining:
• Mechanistic logic-ODE models of signaling pathways regulating CXCL9.
• Active learning to guide perturbation experiments efficiently.
The goal is to identify signaling mechanisms and drug combinations that enhance CXCL9 expression, thereby improving tumor immunogenicity.
2. Key Conclusions
• CXCL9 regulation involves JAK-STAT and NFKB pathways, with PI3K and MAPK also contributing.
• IFNγ strongly induces CXCL9, synergizing with TNFα; synergy is context-dependent and disrupted by certain inhibitors (e.g., IKKi in AsPC1, PI3Ki in BxPC3).
• Mechanistic models accurately capture perturbation responses and reveal cell line-specific regulatory differences.
• Active learning improves experimental efficiency:
o Greedy sampling finds more CXCL9-inducing conditions.
o Uncertainty sampling improves model generalization.
o Combined greedy+uncertainty offers a trade-off.
• Wet lab validation confirms predictions and highlights apoptosis as a confounder in high-dose combinations.
3. Methodology and Model System
• Model system: Two PDAC cell lines (AsPC1 and BxPC3).
• Experimental design: Perturbation screenings with cytokines (IFNγ, TNFα) and inhibitors (JAKi, IKKi, PI3Ki, MEKi, RASi).
• Readout: CXCL9 protein quantified via bead-based immunoassay (flow cytometry).
• Computational approach:
o Curated prior knowledge network (JAK-STAT, NFKB, PI3K-AKT, MAPK, p53).
o Logic-ODE modeling.
o Active learning loop with acquisition functions (greedy, uncertainty, GU, random).
o Benchmarking on synthetic data and iterative wet lab validation.
General Remarks
• Are you convinced of the key conclusions? Yes, the conclusions are supported by strong experimental and computational evidence.
• Context of the work: Fits within efforts to overcome immunotherapy resistance in PDAC by modulating tumor-intrinsic signaling to enhance immune infiltration.
• Nature of the advance: Conceptual: Demonstrates feasibility of coupling active learning with mechanistic models.
• Significance compared to previous knowledge: High. Previous studies used active learning with ML surrogates; this work directly integrates mechanistic models, improving interpretability and biological grounding.

• Audience: Systems biologists, computational modelers, and drug discovery teams.
Major Points
Criticisms
1. Limited scope of PKN: Only five pathways included. Authors exclude other immune-relevant axes (e.g., TGF-β, metabolic signaling).
1. The PKN includes only five major pathways: JAK-STAT, NFKB, PI3K-AKT, MAPK, and p53. Excludes other immune-relevant axes such as TGF-β signaling, metabolic pathways, and stress response pathways. This constrains the hypothesis space: the model can only simulate perturbations acting within these predefined axes. Predictions may miss alternative regulatory mechanisms influencing CXCL9 or immune modulation. Provide a reason as to why only these 5 pathways were included, and could you have gotten similar results with even fewer?
2. The model optimizes for CXCL9 expression only: Immunomodulation is multifactorial; focusing on one chemokine may oversimplify... Drug combinations that improve CXCL9 but worsen other immune parameters could be falsely prioritized.
3. Identifiability issues in NFKB parameters: High variability suggests model uncertainty in this pathway, but this is a major pathway with notable impact on CXCL9 levels. How is this reconciled? Adding a sentence in the second paragraph of the discussion addressing this could help.
4. Apoptosis confounding: I am not convinced that the approach taken by the authors to "adjust" for apoptosis makes sense. I fear that there is some extrapolation with their approach. The result "negative correlation between CXCL9 secretion and apoptosis" (line 486), is an artifact. High-dose combinations reduce viability, affecting CXCL9 measurements.

Reviewer #3:

The manuscript by Wang et al. describes a combined logic-ODE modeling and active learning workflow to conceive targeted combination therapy schemes for perturbing cell-signaling networks. It is here applied to pancreatic cancer cell lines with the aim of identifying conditions with high CXCL9 expression. The approach is analyzed using synthetic data and validated experimentally.

The study adresses an important problem, namely the question of how to assess the combinatorial complexity arising in targeted combination therapy. The presented approach is a thoughtful combination of mechanistic modeling and data-based machine-learning, and it is applied to a scenario of high relevance in cancer therapy, namely chemokine signaling in pancreatic cancer. The detailed analysis of the work-flow using synthetic data nicely shows the pros and cons of various choices to be taken in practice.

Overall, the paper is well-written and well-suited for MSB. Before publication, I feel some parts could be described more clearly and some more detail could be provided, especially regarding model development, and there are some slightly confusing results the authors may want to clarify. Most notably, since round 2 of the validation experiment (Figure 5A) did not result in much improved cxcl9 activity in at least one of the two cell lines, it would make sense to check if further rounds of active learning would help, and how that compares to synthetic data analysis (where mostly round 5 is analysed) - or also to consider additional cell lines.

Specific points:
- In 136/139, it would help to have some more information on the developed mechanistic model (PKN), how many variables and parameters, how were the parameters chosen? I could not find the model in any of the provided files, can it be attached as supplementary table and is source-code available?

- Figure 1B and 5A, it is stated that duplicates are shown for the data points - please provide the raw data at least as supplementary data sheets, how well do the duplicates align?

- Figure 1C, this is a bit to me, is it the same data as in panel B? Why 4 replicates here (instead of 2)? And the values seem to be not the same, e.g. JAKi condition in the 2nd cell line has values ~10000, but in B Jaki in the IFNg + TNF condition is below 5000.

- In 171, "we trained an ensemble of 10 models" - it is not quite clear to me how these 10 models were designed or selected. Is there a specific reason to work with 10 different models per cell line? And are the models shown in Figure 2A/B examples of those 10 models?

- In 173, "... confirming the models' ability to capture the observed perturbation response" - if the different models all capture the data, is that not a sign of strong overfitting, which would make sense given the large networks started from and rather sparse data? I am not sure if that is a problem or actually a feature providing a good basis for the active learning workflow. Anyhow, it would help to analyse and discuss this a bit more.

- Figure 2C, how is the significance test designed? Does it account for all analysed model configurations?

- In 349, "surprisingly ... resulted in lower-than-expected CXCL9 activity" - how can I see that? Figure 5A only presents a single value of cxcl9 activity for each condition, so perhaps it was planned to have a supplement on this topic which I cannot find.

** As a service to authors, EMBO Press offers the possibility to directly transfer declined manuscripts to another EMBO Press title or to the open access journal Life Science Alliance launched in partnership between EMBO Press, Rockefeller University Press and Cold Spring Harbor Laboratory Press. The full manuscript and if applicable, reviewers' reports, are automatically sent to the receiving journal to allow for fast handling and a prompt decision on your manuscript. For more details of this service, and to transfer your manuscript please click on Link Not Available. **

19th Dec 2025

Manuscript Number: MSB-2025-13425R-Q
Title: Active learning-guided mechanistic modeling of CXCL9 regulation in pancreatic cancer
Author: Bi-rong Wang
Maaruthy Yelleswarapu
Lucie Descamps
Federica Eduati

Dear Dr Eduati,

Thank you for your message requesting that we reconsider our recent decision on your manuscript. We have carefully reviewed the points raised in your letter, the original reviewers' reports, and have additionally consulted with an arbitrator who is also an expert in the field.

The arbitrator reviewed the paper positively and expressed reservations about several points raised by the original Reviewer #1. We also find your clarifications on these points to be reasonable. Under these circumstances, we would be pleased to invite you to submit a revised version of the manuscript.

The concerns raised by Reviewer #1 need to be carefully addressed and clarified. All other issues raised by the reviewers should also be satisfactorily addressed. As you may know, our editorial policy generally allows a single round of major revision, so it is essential to provide responses to the reviewers' comments that are as complete as possible. Please feel free to contact me if you would like to discuss any of the reviewers' points in further detail.

On a more editorial level, we would ask you to address the following issues:

- Please provide a .docx formatted version of the manuscript text (including legends for main figures, EV figures and tables). Please make sure that the changes are highlighted to be clearly visible.

- Please provide individual production quality figure files as .eps, .tif, .jpg (one file per figure). When assembling figures, please refer to our figure preparation guideline in order to ensure proper formatting and readability in print as well as on screen: https://media.springernature.com/original/springer-cms/rest/v1/content/27825798/data/v1

-Please provide a .docx formatted letter INCLUDING the reviewers' reports and your detailed point-by-point responses to their comments. As part of the EMBO Press transparent editorial process, the point-by-point response is part of the Review Process File (RPF), which will be published alongside your paper.

-Please note that all corresponding authors are required to supply an ORCID ID for their name upon submission of a revised manuscript.

-We replaced Supplementary Information with Expanded View (EV) Figures and Tables that are collapsible/expandable online. A maximum of 5 EV Figures can be typeset. EV Figures should be cited as 'Figure EV1, Figure EV2" etc... in the text and their respective legends should be included in the main text after the legends of regular figures.

Additional Tables/Datasets should be labeled and referred to as Table EV1, Dataset EV1, etc. Legends have to be provided in a separate tab in case of .xls files. Alternatively, the legend can be supplied as a separate text file (README) and zipped together with the Table/Dataset file.

For the figures and tables that you do NOT wish to display as Expanded View figures, they should be bundled together with their legends in a single PDF file called *Appendix*, which should start with a short Table of Content. Each legend should be below the corresponding Figure/Table in the Appendix. Appendix figures and tables should be referred to in the main text as: "Appendix Figure S1, Appendix Figure S2, Appendix Table S1" etc. See detailed instructions regarding expanded view here: https://link.springer.com/journal/44320/submission-guidelines#cms-Expanded-View-data.

-Before submitting your revision, primary datasets (and computer code, where appropriate) produced in this study need to be deposited in an appropriate public database.
Please remember to provide a reviewer password if the datasets are not yet public.
The accession numbers and database should be listed in a formal "Data Availability" section (placed after Materials & Method) that follows the model below (see also https://link.springer.com/partners/embo-press/editorial-policies#Data%20availability%20statement). Please note that the Data Availability Section is restricted to new primary data that are part of this study.

Data availability
The datasets (and computer code) produced in this study are available in the following databases:
- RNA-Seq data: Gene Expression Omnibus GSE46843 (https://www.ncbi.nlm.nih.gov/geo/query/acc.cgi?acc=GSE46843)
- [data type]: [name of the resource] [accession number/identifier/doi] ([URL or identifiers.org/DATABASE:ACCESSION])
*** Note - All links should resolve to a page where the data can be accessed. ***

- At EMBO Press we ask authors to provide source data for the main manuscript figures. You will receive a separate email with instructions for providing source data with your revised manuscript, including how to upload and organize the files.
Additional information on source data and instruction on how to label the files are available < https://link.springer.com/journal/44320/submission-guidelines#cms-Source-data >

- Our journal encourages inclusion of *data citations in the reference list* to directly cite datasets that were re-used and obtained from public databases. Data citations in the article text are distinct from normal bibliographical citations and should directly link to the database records from which the data can be accessed. In the main text, data citations are formatted as follows: "Data ref: Smith et al, 2001". In the Reference list, data citations must be labeled with "[DATASET]". A data reference must provide the database name, accession number/identifiers and a resolvable link to the landing page from which the data can be accessed at the end of the reference. Further instructions are available at < https://link.springer.com/journal/44320/submission-guidelines#cms-Reference-guidelines >.

- We updated our journal's competing interests policy in January 2022 and request authors to consider both actual and perceived competing interests. Please review the policy (https://link.springer.com/partners/embo-press/editorial-policies#Competing%20interest%20disclosures) and update your competing interests if necessary.
Please use the heading "Disclosure statement and competing interests".

- All Materials and Methods need to be described in the main text using our 'Structured Methods' format. According to this format, the Methods section includes a Reagents and Tools Table (listing key reagents, experimental models, software and relevant equipment and including their sources and relevant identifiers) followed by a Methods and Protocols section describing the methods, ideally using a step-by-step protocol format. The aim is to facilitate adoption of the methodologies across labs.

Please download and fill our Reagents and Tools Table template (.docx), which you can find in our author guidelines: https://link.springer.com/journal/44320/submission-guidelines#structuredmethods.

When submitting your revised manuscript, please do not include the Reagents and Tools Table in the Methods section of the manuscript but upload it as a separate file choosing the file type "Reagent Table".

-Regarding data quantification:
Please ensure to specify the name of the statistical test used to generate error bars and P values, the number (n) of independent experiments (please specify technical or biological replicates) underlying each data point and the test used to calculate p-values in each figure legend. Discussion of statistical methodology can be reported in the materials and methods section, but figure legends should contain a basic description of n, P and the test applied.
Graphs must include a description of the bars and the error bars (s.d., s.e.m.).
Please also include scale bars in all microscopy images.

- Please provide a "standfirst text" summarizing the study in one or two sentences (approximately 250 characters, including space), three to four "bullet points" highlighting the main findings and a "synopsis image" (550px width and 400-600 px height, PNG format) to highlight the paper on our homepage.
Please refer to published papers for examples.

When you resubmit your manuscript, please download our CHECKLIST (https://media.springernature.com/original/springer-cms/rest/v1/content/27825796/data/v1) and include the completed form in your submission.
Please note that the Author Checklist will be published alongside the paper as part of the transparent process .

If you feel you can satisfactorily deal with these points and those listed by the referees, you may wish to submit a revised version of your manuscript. Please attach a covering letter giving details of the way in which you have handled each of the points raised by the referees. A revised manuscript will be reviewed once again, and you probably understand that we can give you no guarantee at this stage that the eventual outcome will be favorable.

I look forward to receiving the revised manuscript soon.

Kind regards,
Jingyi

Jingyi Hou, PhD
Senior Editor
Molecular Systems Biology

We realize that it is difficult to revise to a specific deadline. In the interest of protecting the conceptual advance provided by the work, we recommend a revision within 3 months (19th Mar 2026). Please discuss the revision progress ahead of this time with the editor if you require more time to complete the revisions. Use the link below to submit your revision:

https://msb.msubmit.net/cgi-bin/main.plex

IMPORTANT: Please read our guidelines for revised submissions: https://link.springer.com/journal/44320/submission-guidelines#cms-Revised-submissions

*** PLEASE NOTE *** As part of the EMBO Press transparent editorial process initiative (see our Editorial at https://dx.doi.org/10.1038/msb.2010.72), Molecular Systems Biology publishes online a Review Process File with each accepted manuscripts. This file will be published in conjunction with your paper and will include the anonymous referee reports, your point-by-point response and all pertinent correspondence relating to the manuscript. If you do NOT want this File to be published, please inform the editorial office at contact@molsystbiol.org within 14 days upon receipt of the present letter.

We thank the reviewers and the editor for their careful evaluation of our manuscript and for the constructive comments. We have carefully considered all suggestions and believe that the revisions have helped improve the clarity and quality of the manuscript.

Below we provide a point-by-point response to all comments. For each comment, we indicate how the manuscript has been revised and refer to the corresponding sections (with line numbers) and, in some cases, to new supplementary figures in the revised version. For ease of review, all changes in the manuscript have been marked in red.

Reviewer #1:

In their manuscript entitled „Active learning-guided mechanistic modeling of CXCL9regulation in pancreatic cancer", Eduerti and colleagues describe the development of a logic-ODE model to describe the signal-dependent expression of CXCL9 in two pancreatic cancer cell lines. They train the model based on a perturbation data set where they inhibit some pathways with small molecule inhibitors and/or stimulate cells with TNFa, IFNg or both. They then use the Model to simulate (combinatorial) knockouts and found combinations that led to synergy for the induction between TNFa and IFNg. These simulation results were not tested experimentally.
Subsequently, they implemented an active learning pipeline to learn optimal "experiments" to better refine the model. Briefly, they used an ensemble of models optimized to the original data set, then used this to sample conditions that either maximise CXCL9 induction, or that show maximal uncertainty, or a mixture of both or random. Then they simulated data from one model to refine the parameterization, and iterated these. They then describe which strategies lead to optimized CXCL9 production or better accuracy of the model. For instance, they show that a mixture of greedy and uncertainty performs best when maximizing CXCL9. The authors then perform selected experiments that somewhat cover perturbations that were selected during the training. They then show that these experiments correlate with their findings of the in silico training.
Overall I think the manuscript could nicely illustrate the combination of modelling and active learning for a technical audience. However, the current manuscript has some methodological shortcomings and is not conclusive (both computationally and biologically) to meet the interest for a broader audience of MSB. I am not sure what exactly I learn biologically or methodologically, given the lack of biological testing or deep methodological description.

We thank the reviewer for the summary of our work and for recognizing the potential of combining mechanistic modeling with active learning. We would like to clarify several aspects of the study:

Biological testing.
While the in silico knockout analysis was not experimentally validated, it serves primarily to interpret the trained signaling models and generate mechanistic hypotheses rather than experimentally validated predictions, and is therefore outside the main scope of the study. To address this we have softened the claims on the results of the knockout analysis in the Discussion (line 461-463) and Results (line 204). However, it is important to note that the manuscript does include biological testing through iterative wet-lab perturbation experiments used to validate the active learning framework, where model-selected perturbations were experimentally tested and incorporated into subsequent rounds of model refinement.

Methodological contribution.
The core contribution of this study is the development and application of an active learning pipeline for mechanistic signaling models. To our knowledge, this represents the first implementation of active learning directly coupled with mechanistic models, accompanied by extensive in silico benchmarking and experimental validation.

Biological insights from the mechanistic model.
In parallel, the mechanistic model and its analyses (sensitivity analysis, in silico knockdowns, and bootstrapping) provide biological insights into context-specific regulatory mechanisms controlling CXCL9 expression. We clarified this objective in the revised Introduction (lines 92-95).

We have revised the first paragraph of the Discussion (lines 441-446) to emphasize both the biological insights obtained from the model analysis and the broader applicability of the active learning framework. Additional methodological clarifications are provided below in response to the reviewer's specific concerns.

Major comments:
- The way the authors perform the analysis is circular - they train the model on a model trained by the same data. This training will never be able to perform better than the original model. Thus the trained models can also not be "more" predictive than that model. Why they did not use additional data on the predicted optimal perturbations to retrain the models is unclear to me.

We agree that models trained on synthetic data cannot outperform the model used to generate the data. However, the purpose of this analysis was not to improve upon the generating model, but to compare the behavior of different active learning acquisition strategies under controlled conditions where the ground truth is known.

This benchmarking phase therefore serves to characterize the pipeline in silico. Importantly, this is followed by an application phase in which perturbations selected by the active learning framework are tested experimentally and incorporated into subsequent rounds of model training.

To further emphasize this distinction, we revised several sections of the manuscript to explicitly distinguish between the benchmarking phase, where the pipeline is characterized in silico using a biologically plausible synthetic dataset, and the application phase, where the framework guides and iteratively refines wet-lab perturbation experiments. In particular, we clarified this distinction in the last paragraph of the Introduction (lines 94-95, 98-101), in the Results section describing the active learning workflow and benchmarking analyses (lines 229-231), and in the Methods section where we now explain the rationale for using synthetic data to efficiently explore different design choices before linking the framework to in vitro experiments (lines 708-713). We also added brief clarifications in the Results (lines 348) and Discussion (line 481) to highlight how the in silico characterization of the pipeline informs its subsequent experimental application.

- The fact that the CXCL9 protein levels are higher in the greedy strategy is not surprising, this is what the conditions are optimized for. Why not validating the model with the condition that maximizes CXCL9 expression in the final model pool *after* training? That would show that how the model training actually performs.

We thank the reviewer for this comment and understand the point raised regarding the evaluation of model performance. To provide additional insight, we performed complementary analyses assessing model predictions across the full perturbation space.

Specifically, we assessed how many CXCL9-inducing conditions (“hits”) are identified by each acquisition strategy when evaluating model predictions on all perturbations in the in silico benchmark dataset. As shown in the added Appendix Figure S7A, the greedy strategy identifies a larger number of hits, whereas the uncertainty-based strategy yields a higher fraction of correct hits, with the combined strategy (GU) showing intermediate behavior. These results have been added in lines 259-261.

In addition, to assess how model predictions improve during training, we extended the correlation analysis between model predictions and ground truth across active learning rounds (new Appendix Figure S7B). This analysis shows that uncertainty-based sampling reaches high predictive performance earlier and achieves better overall model generalization than greedy sampling, while GU again shows intermediate behavior, which we added to the Results (lines 268-269).

At the same time, we would like to emphasize that the most relevant performance metric depends on the objective of the analysis. When the goal is to identify CXCL9-inducing conditions ("hits"), the key metric is how well each acquisition strategy identifies hits among the conditions selected at each round, as these correspond to the experiments that would actually be performed in practice. This is the evaluation presented in Figure 4A, where greedy and GU strategies identify more hits in early rounds when the aim is to rapidly discover high-response conditions. In contrast, when the goal is to assess how well the model captures the underlying system and generalizes to unseen perturbations, metrics such as the correlation between predictions and ground truth (R^2) are more informative. This is evaluate in Figure 4B (and new Appendix Figure S7B), showing the higher generalizability of models trained using uncertainty and GU strategies.

Taken together, these analyses are complementary, with hit discovery among selected conditions reflecting practical efficiency and full-space metrics such as R^2 capturing model learning and generalization.

- I find the presentation at many parts very superficial. It would be very important in my eyes which exact conditions were tested in the data of Fig. 5. Were these all conditions? Or selected ones? How does the data actually look like? Also, I would have appreciated a clearer description of the PKN (how exactly does the model look like?

We apologize if some details of the experimental data presentation and model description were not sufficiently clear in the original manuscript.

In Fig. 5, the reported measurements correspond to all the perturbation conditions selected by the active learning pipeline in each round (four conditions per cell line and acquisition strategy). To improve clarity, we clarified this in the caption of Fig. 5 and expanded the description in the Results section. We also added Appendix Table S2, which reports the raw experimental measurements for all tested conditions.

To better illustrate the reliability of the measurements, we now explicitly report the correlation between technical replicates in the Results section (lines 146, 362-363), highlighting the high agreement between replicates.

In addition, to clarify the structure of the mechanistic model, we expanded the description of the prior knowledge network (PKN) in the Appendix Note - Prior knowledge network. We now provide Supplementary Tables in the shared Github repository, listing the proteins and interactions included in the PKN as well as the estimated model parameters (Appendix Table S3-S5). These additions provide a clearer view of the model structure and facilitate reproducibility.

Reviewer #2:

Very well written paper. An interesting, presented tool to drive hypothesis building. Some issues which are listed below, but do not detract from the conceptual advancement and impact on this field. The format of my review follows the editor's requested points.

1. Understanding of the Story

The manuscript addresses the challenge of poor immune infiltration in pancreatic cancer (PDAC) by focusing on CXCL9, a chemokine that recruits $CD8^+$ T cells. The authors propose an integrated experimental-computational pipeline combining:

• Mechanistic logic-ODE models of signaling pathways regulating CXCL9.
• Active learning to guide perturbation experiments efficiently.

The goal is to identify signaling mechanisms and drug combinations that enhance CXCL9expression, thereby improving tumor immunogenicity.

2. Key Conclusions

• CXCL9 regulation involves JAK-STAT and NFKB pathways, with PI3K and MAPK also contributing.
• IFNγ strongly induces CXCL9, synergizing with TNFα; synergy is context-dependent and disrupted by certain inhibitors (e.g., IKKi in AsPC1, PI3Ki in BxPC3).
• Mechanistic models accurately capture perturbation responses and reveal cell line-specific regulatory differences.
• Active learning improves experimental efficiency:
o Greedy sampling finds more CXCL9-inducing conditions.
o Uncertainty sampling improves model generalization.
o Combined greedy+uncertainty offers a trade-off.
• Wet lab validation confirms predictions and highlights apoptosis as a confounder in high-dose combinations.

3. Methodology and Model System

• Model system: Two PDAC cell lines (AsPC1 and BxPC3).
• Experimental design: Perturbation screenings with cytokines (IFNγ, TNFα) and inhibitors (JAKi, IKKi, PI3Ki, MEKi, RASi).
• Readout: CXCL9 protein quantified via bead-based immunoassay (flow cytometry).
• Computational approach:
o Curated prior knowledge network (JAK-STAT, NFKB, PI3K-AKT, MAPK, p53).
o Logic-ODE modeling.
o Active learning loop with acquisition functions (greedy, uncertainty, GU, random).
o Benchmarking on synthetic data and iterative wet lab validation.

General Remarks

• Are you convinced of the key conclusions? Yes, the conclusions are supported by strong experimental and computational evidence.
• Context of the work: Fits within efforts to overcome immunotherapy resistance in PDAC by modulating tumor-intrinsic signaling to enhance immune infiltration.
• Nature of the advance: Conceptual: Demonstrates feasibility of coupling active learning with mechanistic models.

• Significance compared to previous knowledge: High. Previous studies used active learning with ML surrogates; this work directly integrates mechanistic models, improving interpretability and biological grounding.
• Audience: Systems biologists, computational modelers, and drug discovery teams.

We thank the reviewer for the very positive and detailed assessment of our work. We appreciate the reviewer's recognition of the conceptual advance of integrating active learning with mechanistic signaling models and the potential relevance of this approach for systems biology and drug discovery applications. We address the specific points raised below.

Major Points
Criticisms
1. Limited scope of PKN: Only five pathways included. Authors exclude other immune-relevant axes (e.g., TGF-β, metabolic signaling). The PKN includes only five major pathways: JAK-STAT, NFKB, PI3K-AKT, MAPK, and p53. Excludes other immune-relevant axes such as TGF-β signaling, metabolic pathways, and stress response pathways. This constrains the hypothesis space: the model can only simulate perturbations acting within these predefined axes. Predictions may miss alternative regulatory mechanisms influencing CXCL9 or immune modulation. Provide a reason as to why only these 5 pathways were included, and could you have gotten similar results with even fewer?

We thank the reviewer for this important comment regarding the scope of the prior knowledge network (PKN). Below we clarify (i) the rationale for constraining the PKN, (ii) the biological motivation for the selected pathways, and (iii) the effect of network size on the perturbation space explored by the model.

Rationale for constraining the PKN
We agree that the PKN constrains the mechanistic model, but this constraint is important for enabling feasible and context-specific model optimization. Otherwise, the parameter space can become very large relative to the available data, making parameter estimation challenging. To clarify this point, we added explanations in the Methods section (Logic-ODE model – Prior knowledge network; lines 613-622) highlighting how the size of the PKN directly determines the number of model parameters that must be estimated, and how overly large networks would make optimization substantially more difficult.

Biological motivation for the selected pathways
The selected pathways include the main regulatory axes of CXCL9 known from the literature (JAK-STAT and NFKB), together with interacting signaling pathways (PI3K-AKT, MAPK, and p53) that are known to modulate inflammatory signaling and tumor cell responses. In particular, PI3K-AKT and MAPK contain targets of inhibitors used in perturbation experiments (PI3K and MEK), which have been reported to influence

CXCL9 expression. Although the role of p53 in CXCL9 regulation is less well characterized, it was included due to its extensive crosstalk with the other pathways and its central role in tumor signaling. To make this rationale clearer, we expanded the description of the PKN in the Results section (Prior knowledge network curation and experimental design, lines 117-118 and 121-122) and Methods section (lines 655-656) to emphasize both the role of the PKN in constraining the mechanistic model and the biological motivation for including these five pathways as critical signaling pathways in cancer mechanisms. In addition, we expanded the Appendix Note on the Prior Knowledge Network and added references to the supplementary tables added to the Github repository listing the literature sources used to curate the proteins and interactions included in the PKN. We also expanded the Discussion (lines 497 - 513) to discuss how the PKN constrains the prediction space and how the framework could be extended to incorporate additional immune-relevant pathways or multiplexed measurements.

Effect of network size

To follow the reviewer's suggestion of considering a smaller set of pathways, we also applied the active learning pipeline to a reduced PKN containing only the JAK-STAT and NFKB pathways, which represent the main regulatory axes of CXCL9. By construction, this smaller network can only describe perturbations acting on these pathways and therefore restricts the hypothesis space.

When evaluating model performance on the subset of perturbations represented in this reduced network, we observed similar predictive performance compared to the full PKN, suggesting that these core pathways are sufficient to capture these responses. However, the reduced PKN limits the range of perturbations that can be explored and primarily identifies hits within the JAK-STAT and NFKB axes, whereas the full PKN enables exploration of a broader perturbation space and identifies additional hits involving other pathways. We added the results with the small PKN in Appendix Figure S9 and in a new paragraph in the Results section (lines 276-280)

2. The model optimizes for CXCL9 expression only: Immunomodulation is multifactorial; focusing on one chemokine may oversimplify... Drug combinations that improve CXCL9 but worsen other immune parameters could be falsely prioritized.

We agree with the reviewer that tumor immunomodulation is multifactorial and involves multiple signaling pathways and immune mediators. In this study, we focused on CXCL9 because it is a chemokine with increasing evidence for clinical relevance as an immune attractant, particularly in poorly infiltrated ("cold") tumors such as PDAC. At the same time, the regulatory mechanisms controlling CXCL9 expression remain incompletely understood, motivating a focused and mechanistic investigation.

In addition, focusing on a single readout allowed us to systematically characterize the proposed framework that integrates mechanistic modeling with active learning. As this study represents the first application of active learning directly coupled to mechanistic signaling models, restricting the optimization to CXCL9 enabled us to more clearly analyze the behavior of the pipeline and experimentally validate its predictions.

To better reflect the scope of the study, we clarified it at the beginning of the Discussion (lines 441–446). Additionally, we added a comment on the fact that tumor–immune interactions extend beyond CXCL9 regulation and outlined how the framework can be extended to multi-objective settings to jointly optimize multiple immune-related readouts (lines 498, 503–513).

3. Identifiability issues in NFKB parameters: High variability suggests model uncertainty in this pathway, but this is a major pathway with notable impact on CXCL9 levels. How is this reconciled? Adding a sentence in the second paragraph of the discussion addressing this could help.

We thank the reviewer for highlighting this point. Bootstrap-based parameter analysis revealed high variability in several NFKB-related parameters, suggesting limited identifiability of specific interactions within this pathway. Importantly, this variability did not substantially affect the model's ability to reproduce perturbation responses or predict CXCL9 dynamics.

Following the reviewer's suggestion, we revised the Discussion (lines 456–463) to clarify this point. In particular, we now note that uncertainty-based active learning did not identify NFKB-related interactions as a dominant source of predictive uncertainty, indicating that the functional role of NFKB signaling is robustly captured despite limited parameter identifiability. This clarifies how parameter variability and predictive robustness can coexist in the model.

4. Apoptosis confounding: I am not convinced that the approach taken by the authors to "adjust" for apoptosis makes sense. I fear that there is some extrapolation with their approach. The result "negative correlation between CXCL9 secretion and apoptosis" (line 486), is an artifact. High-dose combinations reduce viability, affecting CXCL9measurements.

We thank the reviewer for raising this important point. We agree that the observed negative correlation between CXCL9 secretion and apoptosis likely reflects reduced cell viability at high drug concentrations rather than a direct mechanistic interaction captured by the signaling model. Because apoptosis is not explicitly represented in the PKN, the mechanistic model cannot explain this effect directly.

For this reason, we applied a normalization step to reduce the influence of viability loss on the measured CXCL9 levels used for model analysis. The goal of this adjustment is not to model apoptosis mechanistically, but rather to mitigate measurement bias introduced by reduced cell viability in highly cytotoxic perturbation conditions.

To clarify this point, we added an Appendix Note (Apoptosis correction) providing a detailed explanation of the assumptions and implementation of the normalization step, together with visualizations illustrating the data before and after correction and refer to that in the Methods and in the Results.

Reviewer #3:

The manuscript by Wang et al. describes a combined logic-ODE modeling and active learning workflow to conceive targeted combination therapy schemes for perturbing cell-signaling networks. It is here applied to pancreatic cancer cell lines with the aim of identifying conditions with high CXCL9 expression. The approach is analyzed using synthetic data and validated experimentally.

The study adresses an important problem, namely the question of how to assess the combinatorial complexity arising in targeted combination therapy. The presented approach is a thoughtful combination of mechanistic modeling and data-based machine-learning, and it is applied to a scenario of high relevance in cancer therapy, namely chemokine signaling in pancreatic cancer. The detailed analysis of the work-flow using synthetic data nicely shows the pros and cons of various choices to be taken in practice.

Overall, the paper is well-written and well-suited for MSB. Before publication, I feel some parts could be described more clearly and some more detail could be provided, especially regarding model development, and there are some slightly confusing results the authors may want to clarify.

We thank the reviewer for the positive assessment of our work and for recognizing the relevance of combining mechanistic modeling with active learning to address the combinatorial complexity of targeted perturbations. We appreciate the reviewer's comments regarding the clarity of model development and data presentation, and we have revised several sections of the manuscript to provide additional methodological details and clarify the presentation of experimental results, as described in the point-by-point responses below.

Most notably, since round 2 of the validation experiment (Figure 5A) did not result in much improved cxcl9 activity in at least one of the two cell lines, it would make sense

to check if further rounds of active learning would help, and how that compares to synthetic data analysis (where mostly round 5 is analysed) - or also to consider additional cell lines.

We thank the reviewer for this suggestion and have further investigated the effect of additional active learning rounds through extended analyses.

In our original analysis, we focused on rounds 1 and 2. Benchmarking on synthetic data showed that from round 3 onwards, hit discovery continued to decline while model performance remained largely unchanged. To better illustrate these trends, we have added Appendix Figure S6B–C. These results are consistent with an effect driven by the fixed selection pool: as rounds progress, high-performing conditions are progressively depleted, and later rounds are therefore less likely to identify conditions with higher CXCL9 activity. This suggests the presence of a saturation point, where the best candidates have already been selected, independent of the acquisition strategy.

To further assess this in the *in vitro* setting, we analyzed round 3 using conditions for which additional measurements were already available. We observed that mean CXCL9 activity for the greedy strategy remained stable across rounds in AsPC1 and decreased in BxPC3 (Figure below, not included in the manuscript). These trends are consistent with our synthetic data results. Based on these observations, we did not pursue additional rounds, as later iterations are unlikely to provide further insight into differences between acquisition functions. Instead, the diminishing returns appear to be primarily driven by depletion of the selection pool rather than the sampling strategy itself.

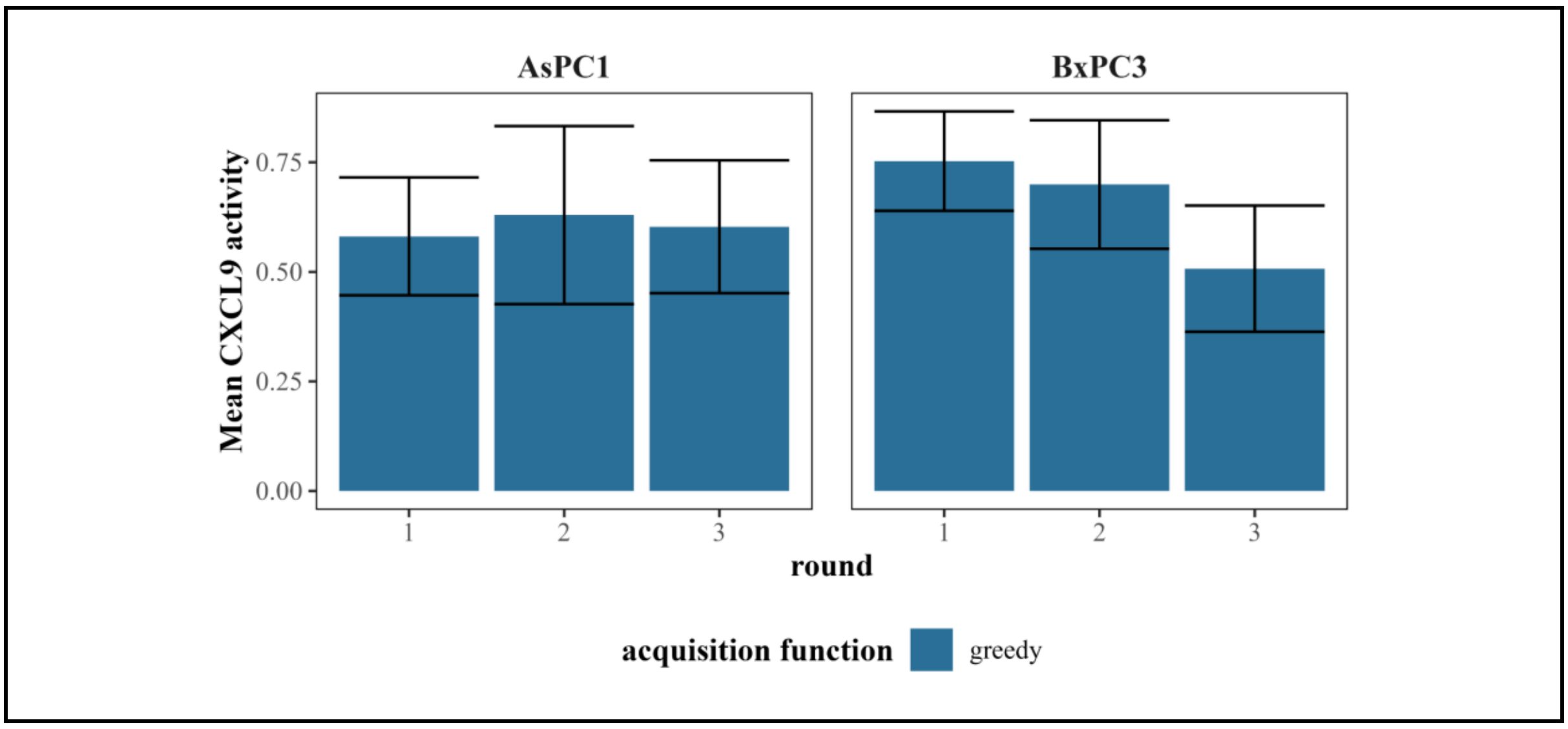

Extending the analysis to additional cell lines would primarily address generality, whereas the focus of this study is on establishing and experimentally validating a mechanistic active learning framework. We therefore prioritized in-depth

characterization in two PDAC cell lines, which already capture distinct regulatory behaviors, rather than increasing the number of systems

Specific points:
- In 136/139, it would help to have some more information on the developed mechanistic model (PKN), how many variables and parameters, how were the parameters chosen? I could not find the model in any of the provided files, can it be attached as supplementary table and is source-code available?

We thank the reviewer for this suggestion and agree that providing additional details on the mechanistic model improves transparency and reproducibility.

To clarify how the prior knowledge network (PKN) translates into the mechanistic model, we expanded the description of the model structure and parameterization in several parts of the manuscript. In the Results section ("Prior knowledge network curation and experimental design", lines 133, 139-141) we now explain how the PKN defines the architecture of the logic-ODE model. In the Methods section (Logic-ODE model – Model structure, lines 620-622) we added a more detailed explanation of how proteins and interactions in the PKN translate into model variables and parameters within the logic-ODE framework. In addition, the Appendix Note "Prior knowledge network" now includes a dedicated subsection describing the translation of the PKN into the system of logic-ODE equations and the corresponding parameterization.

To further facilitate reproducibility, we also added Appendix Tables S3-S5 reporting the optimized model parameters. Finally, all model files are available in the GitHub repository (link provided in the data and code availability section) associated with the manuscript (folder output/mechanistic_model), together with the code required to reproduce all analyses presented in the paper.

- Figure 1B and 5A, it is stated that duplicates are shown for the data points - please provide the raw data at least as supplementary data sheets, how well do the duplicates align?

We thank the reviewer for this suggestion and agree that providing the raw data and information on replicate agreement improves transparency of the experimental results.

We now provide the raw experimental measurements underlying Figures 1B and 5A in Appendix Table S1 and S2, also available in the Github repository (data + code to reproduce figures). In addition, we clarified in the Results section (lines 146, 362-363) that the replicate measurements show high agreement. For the perturbation screenings shown in Figure 1, replicates show high correlations ($R > 0.80$), and similarly high agreement was observed for the perturbation experiments performed

during the active learning rounds (Figure 5). These values are now explicitly reported in the manuscript and added as additional figures in Appendix Figures S2C-D and S8B.

- Figure 1C, this is a bit to me, is it the same data as in panel B? Why 4 replicates here (instead of 2)? And the values seem to be not the same, e.g. JAKi condition in the 2nd cell line has values ~10000, but in B Jaki in the IFNg + TNF condition is below 5000.

We thank the reviewer for pointing out this lack of clarity. Figure 1C is based on the same experimental dataset as Figure 1B, but focuses specifically on the IFNγ–TNFα synergy analysis. While Figure 1B shows CXCL9 responses averaged across replicates for all perturbation conditions, Figure 1C uses all replicate measurements (two technical replicates for each of two biological replicates), resulting in four data points per condition. To clarify this, we expanded the description in the Figure 1 caption and in the Results section (Shared and distinct CXCL9 responses in AsPC1 and BxPC3 cell lines, lines 158-159) explaining how the data shown in Figure 1C relate to the measurements summarized in Figure 1B.

In revisiting the figure following the reviewer's comment, we also identified an error in the compilation of the original Figure 1C, where labels and significance test results were not correctly aligned with the plotted data. This has been corrected in the revised figure, and the "Measured" values now correspond to the IFNγ + TNFα measurements shown in Figure 1B. This correction does not affect the statistical results or the conclusions of the analysis.

- In 171, "we trained an ensemble of 10 models" - it is not quite clear to me how these 10 models were designed or selected. Is there a specific reason to work with 10 different models per cell line? And are the models shown in Figure 2A/B examples of those 10 models?

We thank the reviewer for pointing out that this aspect of the model training was not sufficiently clear. For each cell line, we performed ten independent optimization runs starting from different initial parameter values and obtained an ensemble of fitted models. Because the optimization landscape is non-convex, individual runs can converge to slightly different parameter sets that reproduce the experimental data similarly well. To avoid downstream analyses depending on a single fitted model, we therefore used an ensemble approach and averaged model simulations across the ten optimized models.

All models within the ensemble share the same architecture defined by the prior knowledge network (PKN) and therefore only differ in their fitted parameter values. Consequently, the networks shown in Figure 2A/B represent the common model

structure used for all models in the ensemble rather than individual examples. To define which single edge knockouts had significant effects, we statistically compared the model simulations within the ensemble with and without the knockout.

To clarify this point, we added an explanation of the ensemble modeling strategy in the Results section (In silico knockouts and sensitivity analysis identify key regulators and cell line-specific control of CXCL9, lines 171-174) and in the Methods section (Logic-ODE model – Model structure, lines 635-638), where we now describe the independent optimization runs, the use of the ensemble for averaging simulations, and the objective function threshold applied to retain fitted models.

- In 173, "... confirming the models' ability to capture the observed perturbation response" - if the different models all capture the data, is that not a sign of strong overfitting, which would make sense given the large networks started from and rather sparse data? I am not sure if that is a problem or actually a feature providing a good basis for the active learning workflow. Anyhow, it would help to analyse and discuss this a bit more.

We thank the reviewer for raising this important point. Indeed, when fitting relatively large signaling network models to limited perturbation data, multiple parameterizations can reproduce the observed responses. This reflects the well-known issue of limited parameter identifiability in mechanistic signaling models rather than classical overfitting. To mitigate overparameterisation during model training, we included L1 regularisation on interaction strength parameters and analysed parameter stability using bootstrap resampling and multi-parametric sensitivity analysis (Appendix Figure S5). These analyses allow us to assess how consistently parameters are estimated across repeated model fits. While some parameters show variability—particularly in the NFKB pathway—many key interactions remain consistently constrained by the data.

Rather than selecting a single fitted model, we therefore retain an ensemble of well-fitting models obtained from independent optimisation runs, which captures uncertainty in parameter space and is subsequently used to estimate prediction uncertainty in the active learning framework. To clarify this point, we revised the corresponding sentence in the Results (lines 171-177) to explicitly state that several parameterizations can reproduce the observed perturbation responses and that an ensemble of models is retrained. We also added a sentence in the Discussion (lines 456 - 461) noting that variability between fitted models reflects limited parameter identifiability rather than reduced predictive capability.

- Figure 2C, how is the significance test designed? Does it account for all analysed model configurations?

We thank the reviewer for requesting clarification. The significance test in Figure 2C follows the same logic as the synergy test described for Figure 1C. However, instead of experimental measurements, we use the averaged simulations from the ensemble of optimized models to compare the predicted CXCL9 response for the combined IFNγ + TNFα condition with the expected additive response from the individual cytokine stimulations. This ensures that the test accounts for all analysed model configurations.

To clarify this, we added an explanation in the Results (In silico knockouts and sensitivity analysis identify key regulators and cell line-specific control of CXCL9, line 181) and expanded the description of the hypothesis testing procedure in the Materials and Methods (CXCL9 data processing and analysis, lines 577-585).

- ln 349, "surprisingly ... resulted in lower-than-expected CXCL9 activity" - how can I see that? Figure 5A only presents a single value of cxcl9 activity for each condition, so perhaps it was planned to have a supplement on this topic which I cannot find.

We thank the reviewer for pointing out that this observation was not sufficiently supported by the figures in the submitted version. In the original manuscript we referred to the underlying experimental measurements but did not include a visualization of all tested perturbation conditions.

To make this clearer, we added a new Appendix Figure S8A, which shows the CXCL9 measurements for all perturbation conditions selected during the active learning experiments (four conditions per model and acquisition strategy). This visualization makes it possible to inspect the individual experimental measurements and highlights that several conditions—particularly in the greedy acquisition strategy—resulted in lower-than-expected CXCL9 activity, including examples such as the PI3Ki+ERKi combination.

28th Apr 2026

Manuscript Number: MSB-2025-13425RR
Title: Active learning-guided mechanistic modeling of CXCL9 regulation in pancreatic cancer
Author: Bi-rong Wang
Maaruthy Yelleswarapu
Lucie Descamps
Federica Eduati

Dear Dr Eduati,

Thank you for submitting the revised version of your manuscript. We have now received feedback from Reviewer #3, who was asked to re-evaluate your revised manuscript and your response to the previous comments from Reviewer #1. While the reviewer acknowledges improvements in clarity, they note that their main concern regarding the experimental validation of the active learning pipeline remains insufficiently addressed.

In particular, the reviewer raises questions about the discrepancy between the multi-round benchmarking on synthetic data and the limited number of experimental rounds performed, as well as the extent to which the experimental results support the synthetic data analysis.

We therefore ask that you carefully revise the manuscript to address these points. In particular, it is essential to:

1. More clearly explain which aspects of the active learning framework are experimentally validated, and how the results from the synthetic data analysis relate to and can be compared with the experimental results.

2. Expand the Discussion to explicitly acknowledge the current limitations of the experimental validation, discuss alternative experimental approached proposed by the reviewers, as well as the scope of the conclusions that can be drawn.

3. Temper claims regarding novel biological insights and placing greater emphasis on the methodological contribution of the study.

4. All the other remaining issues need to be addressed as well.

On a more editorial level:

1. Remove the "Authors' contribution" section from the manuscript file.

2. Remove all figures from the manuscript file and retain only the figure legends at the end of the manuscript.

3. Please add missing callouts for Appendix Table S1 and S3, Appendix Figure S10.

4. "Table 1" is mentioned in the manuscript. It appears to refer to the Reagent and Tool Table. If so, please update the callout accordingly.

5. The references need to be formatted according to the Molecular Systems Biology reference style. Please list up to 10 co-authors of a paper before adding et al. in the reference list. Citations should be listed in alphabetical order.

6. "Competing interests" should be renamed to "Disclosure and Competing Interests Statement".

7. On the title page, authors should be listed on a single line with full names (not initials).

8. Data availability: The Code Availability section should be incorporated into the Data Availability section. Please remove the separate "Code Availability" heading.

9. Funding information: Grant 015.021.065 is listed in the submission system but not in the manuscript text. Please resolve this discrepancy.

10. Appendix:
- Please remove the author list and affiliations from the first page.
- Table of Contents: Some items appear to be missing; please double-check and correct as needed.
- Nomenclature should be standardized to "Appendix Table Sx" and "Appendix Figure Sx" throughout, including in the Table of

Contents.
- Please provide the Appendix in PDF format.

11. Source data:
- Source data for main figures: should be uploaded as one (zipped) file /figure, and named as "manuscriptID_SourceDataForFigure x"
- Source data for supplementary figures can be combined in one file.

12. "Materials and methods" need to be labeled as 'Methods'.

13. Please address the following issues in figure legends:
- Please note that the exact p values are not provided in the legend of figure 1C
- Please note that the box plots need to be defined in terms of minima, maxima, centre, bounds of box and whiskers, and percentile in the legends of figures 5A, C
- Please note that information related to n is missing in the legends of figures 5F, H; 5C"

14. The manuscript sections should be in the following order: Title page - Abstract - Introduction - Results - Discussion - Methods -Data Availability - Acknowledgments - Disclosure and Competing Interests Statement - References - Figure Legends - (Main Tables with legends if applicable) - Expanded View Figure Legends.

If you feel you can satisfactorily deal with these points and those listed by the reviewer, you may wish to submit a revised version of your manuscript. Please attach a covering letter giving details of the way in which you have handled each of the points raised by the referees. A revised manuscript may be once again subject to review and you probably understand that we can give you no guarantee at this stage that the eventual outcome will be favorable.

I look forward to receiving a revised version soon.

Kind regards,
Jingyi

Jingyi Hou, PhD
Senior Editor
Molecular Systems Biology

We realize that it is difficult to revise to a specific deadline. In the interest of protecting the conceptual advance provided by the work, we recommend a revision within 3 months (27th Jul 2026). Please discuss the revision progress ahead of this time with the editor if you require more time to complete the revisions. Use the link below to submit your revision:

https://msb.msubmit.net/cgi-bin/main.plex

IMPORTANT: Please read our guidelines for revised submissions: https://link.springer.com/journal/44320/submission-guidelines#cms-Revised-submissions

*** PLEASE NOTE *** As part of the EMBO Press transparent editorial process initiative (see our Editorial at https://dx.doi.org/10.1038/msb.2010.72), Molecular Systems Biology publishes online a Review Process File with each accepted manuscripts. This file will be published in conjunction with your paper and will include the anonymous referee reports, your point-by-point response and all pertinent correspondence relating to the manuscript. If you do NOT want this File to be published, please inform the editorial office at contact@molsystbiol.org within 14 days upon receipt of the present letter.

Reviewer #3:

In revision, the authors addressed most of the questions regarding clarity of presentation, especially outline of the model, handling of experimental replicates and statistical analysis (although a few questions remain, see below). However, my main concern about validation of the active learning pipeline remains. The main argument for not going into more rounds of experiments seems to be the following in the response letter: "Benchmarking on synthetic data showed that from round 3 onwards, hit discovery continued to decline while model performance remained largely unchanged." However, the results in

Figure 4A do not seem to support that: looking at the "4 conditions per round" line (I understand that this matches the experiments), the number of hits shows an almost linear increase up to round 5, with false positives only appearing in the "random" condition (and I am not sure how model performance is assessed and how I can check the statement on that). Also, I still do not understand that much of the in silico analysis is focussed on round 5 while experiments only proceed to round 2. Overall, I do not quite understand what is exactly validated, that is which results from the synthetic data analysis can be directly compared to experiments.

Besides, the new Figure S6 and the additional plot in the response letter also do not really make things clearer to me. One could argue here that the training in many cases rather leads to conditions with reduced CXCL9 activity, somewhat in contradiction to the claims of the authors (why is CXCL9 vs rounds of training not shown more directly in the main paper?).

As mentioned before, an alternative to more rounds of (experimental) training could be more replicates and cell lines and based on that a deeper statistical analysis of round 2 that allows comparison to synthetic data; another (even better) possibility was raised by Reviewer 1, that is independent experimental validation of the model obtained after training - for example by an in-silico knockout approach such as in Figure 2 and testing some predictions.

Minor points:
- I still do not quite understand the "apoptosis correction". Possibly there was a missunderstanding - my point was that I could not find any values in the manuscript without application of the correction, and so it was (and is) not possible to assess the effect of the correction as well as the "surprisingly" statement. That does not seem to have changed after revision.

- I do now understand the statistics workflow, but I have some concerns about handling biological and technical replicates together in the analysis. Further, while correlation analysis indicates a similar trend among replicates, individual values show substantial deviation in some cases so it would make sense to have at least triplicates. Standard deviations based on just two replicates generally should be avoided (such as in Figure S8).

Reviewer #3's evaluation of your response to Reviewer #1

Regarding the points raised by Reviewer #1, I agree with the authors that the synthetic data generation is a valid and useful approach to establish the method and therefore I would rather not critisize that as "circular". However, as I wrote also in my review, I agree with Reviewer #1 that experimental validation is not very strong in the current form, and also that biological results are not very clear. Generally, I do not see major changes in the revised version that go beyond clearer descriptions, which I find a bit disappointing, although I still think the approach taken by the authors is interesting.

Thank you for submitting the revised version of your manuscript. We have now received feedback from Reviewer #3, who was asked to re-evaluate your revised manuscript and your response to the previous comments from Reviewer #1. While the reviewer acknowledges improvements in clarity, they note that their main concern regarding the experimental validation of the active learning pipeline remains insufficiently addressed.
In particular, the reviewer raises questions about the discrepancy between the multi-round benchmarking on synthetic data and the limited number of experimental rounds performed, as well as the extent to which the experimental results support the synthetic data analysis.
We would like to thank the Editor and the Reviewer for careful consideration of our manuscript and we provide our point-by-point reply below. We have also addressed the editorial comments in our revised manuscript.

We therefore ask that you carefully revise the manuscript to address these points. In particular, it is essential to:
1. More clearly explain which aspects of the active learning framework are experimentally validated, and how the results from the synthetic data analysis relate to and can be compared with the experimental results.
We revised the Results section (**lines 249-254**) to more clearly distinguish the goals of the *in silico* benchmarking and the *in vitro* validation experiments. Specifically, we now clarify that the synthetic data analysis was used to systematically characterize acquisition strategy behavior across several iterative rounds, while the experimental validation was designed to test whether the key relative differences between acquisition strategies observed in early rounds are reproducible under realistic laboratory conditions. We additionally clarified the rationale for limiting the experimental validation to two rounds (**lines 368-383**) and more explicitly discussed how the experimental observations relate to the in silico benchmarking results (**lines 432, 447-452**). These points are further addressed in our response to Reviewer #3, comment 1.

2. Expand the Discussion to explicitly acknowledge the current limitations of the experimental validation, discuss alternative experimental approached proposed by the reviewers, as well as the scope of the conclusions that can be drawn.
Following the editor's recommendation, we expanded the Discussion section (**lines 544-551**) to more clearly contextualize the current experimental validation and the scope of the biological conclusions, while outlining future directions for broader validation of the framework. These points are discussed in more detail in our responses to Reviewer #3 comments 3 and 5.

3. Temper claims regarding novel biological insights and placing greater emphasis on the methodological contribution of the study.
We revised the Abstract (**lines 30, 31)**, Introduction (**lines 95, 102-106**) and Discussion (**lines 544-551**) to more clearly position the primary contribution of this study as the development and systematic evaluation of a novel active learning

framework for perturbation prioritization using mechanistic models. In the Abstract and Introduction, we revised the framing of the study to emphasize the methodological contribution of the active learning pipeline and clarified the scope of the experimental validation. In the Discussion, we tempered claims related to biological insight generation and more explicitly contextualized the experimental results as experimental validation of the framework under realistic wet-lab conditions.

4. All the other remaining issues need to be addressed as well.
Please see point-by-point responses to the reviewer's comments.

Reviewer #3:

In revision, the authors addressed most of the questions regarding clarity of presentation, especially outline of the model, handling of experimental replicates and statistical analysis (although a few questions remain, see below). However, my main concern about validation of the active learning pipeline remains. The main argument for not going into more rounds of experiments seems to be the following in the response letter: "Benchmarking on synthetic data showed that from round 3 onwards, hit discovery continued to decline while model performance remained largely unchanged." However, the results in Figure 4A do not seem to support that: looking at the "4 conditions per round" line (I understand that this matches the experiments), the number of hits shows an almost linear increase up to round 5, with false positives only appearing in the "random" condition (and I am not sure how model performance is assessed and how I can check the statement on that). Also, I still do not understand that much of the in silico analysis is focussed on round 5 while experiments only proceed to round 2. Overall, I do not quite understand what is exactly validated, that is which results from the synthetic data analysis can be directly compared to experiments.

We thank the reviewer for this careful reading and apologize that our previous response did not clearly explain the purpose of the experimental validation and its relationship to the *in silico* benchmarking. We agree that it is important to explicitly specify what is being validated experimentally.

The goal of the in vitro validation was to validate the main differences between acquisition strategies observed in the *in silico* benchmarking. Specifically, the benchmarking showed that greedy sampling is more effective at rapidly identifying high-activity hits, whereas uncertainty sampling is more effective at improving model generalisability. GU provided an interesting compromise between these two objectives. Since iterative wet-lab experimentation is inherently constrained in the number of rounds that can be practically performed, we focused the experimental validation on early rounds, where the *in silico* benchmarking already showed the emergence of clear differences between acquisition strategies.

- **Hit discovery.** Figure 4A already shows that differences in hit discovery between acquisition strategies are visible from the early rounds onward. This behavior becomes even clearer when considering cumulative CXCL9 activity (new **Appendix Figure S6B-C**). For the experimental setting (N(add) = 4), greedy and GU consistently identified conditions with higher cumulative CXCL9 activity than uncertainty or random sampling already from round 1 onward, and these differences remained qualitatively stable across subsequent rounds. For example, uncertainty sampling never reached the cumulative CXCL9 activity achieved by greedy or GU in any round (average differences 3.44 and 3.10 across rounds for greedy and GU, respectively).

These results indicate that the characteristic acquisition behavior emerges early in the iterative process.

- **Model generalizability.** Predictive performance was evaluated using the Pearson R^2 between predicted and observed values across both training and unseen data to allow comparison between acquisition functions and rounds (Appendix Figure S7B). Across acquisition strategies, the largest improvements occurred during the first two rounds, while later rounds yielded substantially smaller gains. Specifically, the average increase in R^2 between rounds 1 and 2 was 0.01 (greedy), 0.10 (uncertainty), 0.11 (GU), whereas the average changes across acquisition functions decreased to 0.05 (round 3), 0.02 (round 4) and 0.003 (round 5). This indicates that most improvements in model generalisability are achieved early during the iterative process.

While the experimental validation was limited to two rounds due to the practical constraints of iterative wet-lab experimentation, the *in silico* benchmarking was extended to five rounds to leverage the flexibility and scalability of computational experiments and more comprehensively characterize acquisition strategy behavior across iterative rounds. Most analyses in **Figure 4** and **Appendix Figures S6–S7** include all iterative rounds, while only **Figure 4B** and **Figure 4F–H** focus specifically on round 5 as a representative endpoint summary, but analyses across all rounds are provided in **Appendix Figures S6–S7**.

We revised the manuscript accordingly to more clearly distinguish the goals of the *in silico* benchmarking and the *in vitro* validation experiments, and to clarify the rationale for limiting the experimental validation to two rounds (**lines 368-383, 447-452)**.

Besides, the new Figure S6 and the additional plot in the response letter also do not really make things clearer to me. One could argue here that the training in many cases rather leads to conditions with reduced CXCL9 activity, somewhat in contradiction to the claims of the authors (why is CXCL9 vs rounds of training not shown more directly in the main paper?).
We thank the reviewer for this important comment and agree that the previous presentation did not clearly show how CXCL9 activity changes across active learning rounds. We therefore revised **Appendix Figure S6B-C** to show the cumulative CXCL9 activity of the identified hits across rounds of optimization.

We would like to clarify that early active learning rounds preferentially identify the strongest CXCL9-inducing conditions, which are subsequently incorporated into the training set and therefore removed from the pool of available candidate conditions. As a result, later rounds increasingly select conditions with smaller effects. This effect is particularly pronounced for greedy approaches, which prioritize exploitation of the highest-scoring conditions in early rounds. As a result, the cumulative CXCL9 activity progressively stabilizes in both the synthetic (**Appendix Figure S6B-C**) and

in vitro (plot from previous response letter) experiments, as newly identified conditions tend to have smaller effects than those selected earlier.

We revised the manuscript (**lines 262-267**) to explicitly clarify this behavior in the context of active learning.

As mentioned before, an alternative to more rounds of (experimental) training could be more replicates and cell lines and based on that a deeper statistical analysis of round 2 that allows comparison to synthetic data; another (even better) possibility was raised by Reviewer 1, that is independent experimental validation of the model obtained after training - for example by an in-silico knockout approach such as in Figure 2 and testing some predictions.
We thank the reviewer for these suggestions. Following the editor's recommendation, we expanded the Discussion section to explicitly acknowledge the current limitations of the experimental validation and discuss alternative experimental approaches proposed by the reviewers, including additional replicates and cell lines, as well as independent validation of model predictions through targeted perturbation experiments. These additions can be found in **lines 544-551**.

Minor points:
- I still do not quite understand the "apoptosis correction". Possibly there was a missunderstanding - my point was that I could not find any values in the manuscript without application of the correction, and so it was (and is) not possible to assess the effect of the correction as well as the "surprisingly" statement. That does not seem to have changed after revision.
We apologize for the incorrect figure references in the Appendix Note – Apoptosis correction, which made it difficult to locate the relevant data. **Appendix Figure S8A** shows the CXCL9 activity values without apoptosis correction, while **Appendix Figure S8C** (formerly Appendix Figure S10) illustrates the negative correlation between apoptosis and CXCL9 activity that motivated the application of the correction. We additionally revised the text (**lines 397, 399, 406**) to directly refer to these figures and removed the "surprisingly" statement to avoid ambiguity.

- I do now understand the statistics workflow, but I have some concerns about handling biological and technical replicates together in the analysis. Further, while correlation analysis indicates a similar trend among replicates, individual values show substantial deviation in some cases so it would make sense to have at least triplicates. Standard deviations based on just two replicates generally should be avoided (such as in Figure S8).
We thank the reviewer for their careful evaluation of our statistical workflow. For statistical testing, biological and technical replicates were combined only for the synergy analyses in **Figure 1C** due to the limited number of biological replicates (n = 2). We acknowledge that this limits the statistical power and robustness of these analyses. Accordingly, the primary purpose of the synergy tests was to support qualitative trends rather than to serve as the basis for strong statistical conclusions.

To improve transparency, we revised the conclusions associated with **Figure 1C** to better clarify the purpose and limitations of the statistical testing (**lines 162-165**).

We would also like to clarify that the main methodological conclusions of this work, related to the active learning framework, are not dependent on the number of biological replicates, as model performance was evaluated using an ensemble of ten models. However we now acknowledge in the Discussion (**lines 544-551**) that increasing the number of biological replicates would be important to further strengthen the biological conclusions drawn from the experimental data.

Lastly, we thank the reviewer for the suggestion regarding **Appendix Figure S8A**. We revised the figure to display the individual data points rather than standard deviations derived from two replicates.

Reviewer #3's evaluation of your response to Reviewer #1
Regarding the points raised by Reviewer #1, I agree with the authors that the synthetic data generation is a valid and useful approach to establish the method and therefore I would rather not critisize that as "circular". However, as I wrote also in my review, I agree with Reviewer #1 that experimental validation is not very strong in the current form, and also that biological results are not very clear. Generally, I do not see major changes in the revised version that go beyond clearer descriptions, which I find a bit disappointing, although I still think the approach taken by the authors is interesting.
We thank the reviewer for carefully evaluating also our responses to Reviewer #1, and for acknowledging the value of the synthetic data framework. We agree that the revisions primarily focused on improving the clarity and interpretation of the work, while also including additional computational analyses rather than substantial new experimental data. Following the editor's recommendation, and as addressed in more detail in our responses above, we revised the Discussion (**lines 544-551**) to more clearly acknowledge the current limitations of the experimental validation and biological interpretation, emphasizing that the main contribution of this study is the development and systematic evaluation of a novel active learning framework for perturbation prioritization using mechanistic models. The experimental results are therefore positioned as experimental validation of this methodological framework under realistic wet-lab conditions.

27th May 2026
Manuscript number: MSB-2025-13425RRR
Title: Active learning-guided mechanistic modeling of CXCL9 regulation in pancreatic cancer

Dear Dr Eduati,

Thank you again for sending us your revised manuscript. We are now satisfied with the modifications made and I am pleased to inform you that your paper has been accepted for publication.

Your manuscript will be processed for publication by EMBO Press. It will be copy edited and you will receive page proofs prior to publication. Please note that you will be contacted by Springer Nature Author Services to complete licensing and payment information.

You may qualify for financial assistance for your publication charges - either via a Springer Nature fully open access agreement or an EMBO initiative. Check your eligibility: https://link.springer.com/journal/44320/how-to-publish-with-us

Should you be planning a Press Release on your article, please notify the production team via this form in order to coordinate publication and release dates: https://forms.cloud.microsoft/e/eH4A8JVP6g

If you have any questions, please do not hesitate to contact the Editorial Office. Thank you for your contribution to Molecular Systems Biology.

Sincerely,
Jingyi

Jingyi Hou, PhD
Senior Editor
Molecular Systems Biology

>>> Please note that it is Molecular Systems Biology policy for the transcript of the editorial process (containing referee reports and your response letter) to be published as an online supplement to each paper. If you do NOT want this, you will need to inform the Editorial Office via email immediately. More information is available here: https://link.springer.com/partners/embo-press/editorial-policies#Peer%20review

EMBO Press Author Checklist

Corresponding Author Name: Federica Eduati	
Journal Submitted to: Molecular Systems Biology	
Manuscript Number: MSB-2025-13425	

USEFUL LINKS FOR COMPLETING THIS FORM
The EMBO Journal - Author Guidelines
EMBO Reports - Author Guidelines
Molecular Systems Biology - Author Guidelines
EMBO Molecular Medicine - Author Guidelines

Reporting Checklist for Life Science Articles (updated January
This checklist is adapted from Materials Design Analysis Reporting (MDAR) Checklist for Authors. MDAR establishes a minimum set of requirements in transparent reporting in the life sciences (see Statement of Task: 10.31222/osf.io/9sm4x). Please follow the journal's guidelines in preparing your
Please note that a copy of this checklist will be published alongside your article.

Abridged guidelines for figures
1. Data
The data shown in figures should satisfy the following conditions:
- ➞ the data were obtained and processed according to the field's best practice and are presented to reflect the results of the experiments in an accurate and unbiased manner.
- ➞ ideally, figure panels should include only measurements that are directly comparable to each other and obtained with the same assay.
- ➞ plots include clearly labeled error bars for independent experiments and sample sizes. Unless justified, error bars should not be shown for technical
- ➞ if n<5, the individual data points from each experiment should be plotted. Any statistical test employed should be justified.
- ➞ Source Data should be included to report the data underlying figures according to the guidelines set out in the authorship guidelines on Data

2. Captions
Each figure caption should contain the following information, for each panel where they are relevant:
- ➞ a specification of the experimental system investigated (eg cell line, species name).
- ➞ the assay(s) and method(s) used to carry out the reported observations and measurements.
- ➞ an explicit mention of the biological and chemical entity(ies) that are being measured.
- ➞ an explicit mention of the biological and chemical entity(ies) that are altered/varied/perturbed in a controlled manner.
- ➞ the exact sample size (n) for each experimental group/condition, given as a number, not a range;
- ➞ a description of the sample collection allowing the reader to understand whether the samples represent technical or biological replicates (including how many animals, litters, cultures, etc.).
- ➞ a statement of how many times the experiment shown was independently replicated in the laboratory.
- ➞ definitions of statistical methods and measures:
  - common tests, such as t-test (please specify whether paired vs. unpaired), simple χ2 tests, Wilcoxon and Mann-Whitney tests, can be unambiguously identified by name only, but more complex techniques should be described in the methods section;
  - are tests one-sided or two-sided?
  - are there adjustments for multiple comparisons?
  - exact statistical test results, e.g., P values = x but not P values < x;
  - definition of 'center values' as median or average;
  - definition of error bars as s.d. or s.e.m.

Please complete ALL of the questions below.
Select "Not Applicable" only when the requested information is not relevant for your study.

Materials

Newly Created Materials	Information included in the manuscript?	In which section is the information available? (Reagents and Tools Table, Materials and Methods, Figures, Data Availability Section)
New materials and reagents need to be available; do any restrictions apply?	Yes	Reagents and Tools Table

Antibodies	Information included in the manuscript?	In which section is the information available? (Reagents and Tools Table, Materials and Methods, Figures, Data Availability Section)
For **antibodies** provide the following information:<br>- Commercial antibodies: RRID (if possible) or supplier name, catalogue number and or/clone number<br>- Non-commercial: RRID or citation	Not Applicable	

DNA and RNA sequences	Information included in the manuscript?	In which section is the information available? (Reagents and Tools Table, Materials and Methods, Figures, Data Availability Section)
**Short novel DNA or RNA including primers, probes**: provide the sequences.	Not Applicable	

Cell materials	Information included in the manuscript?	In which section is the information available? (Reagents and Tools Table, Materials and Methods, Figures, Data Availability Section)
**Cell lines:** Provide species information, strain. Provide accession number in repository **OR** supplier name, catalog number, clone number, and/**OR** RRID.	Yes	Reagents and Tools Table
**Primary cultures:** Provide species, strain, sex of origin, genetic modification status.	Not Applicable	
Report if the cell lines were recently **authenticated** (e.g., by STR profiling) and tested for mycoplasma contamination.	Not Applicable	

Experimental animals	Information included in the manuscript?	In which section is the information available? (Reagents and Tools Table, Materials and Methods, Figures, Data Availability Section)
**Laboratory animals or Model organisms:** Provide species, strain, sex, age, genetic modification status. Provide accession number in repository **OR** supplier name, catalog number, clone number, **OR** RRID.	Not Applicable	
**Animal observed in or captured from the field:** Provide species, sex, and age where possible.	Not Applicable	
Please detail **housing and husbandry conditions**.	Not Applicable	

Plants and microbes	Information included in the manuscript?	In which section is the information available? (Reagents and Tools Table, Materials and Methods, Figures, Data Availability Section)
**Plants:** provide species and strain, ecotype and cultivar where relevant, unique accession number if available, and source (including location for collected wild specimens).	Not Applicable	
**Microbes:** provide species and strain, unique accession number if available, and source.	Not Applicable	

Human research participants	Information included in the manuscript?	In which section is the information available? (Reagents and Tools Table, Materials and Methods, Figures, Data Availability Section)
If collected and within the bounds of privacy constraints report on age, sex and gender or ethnicity for all study participants.	Not Applicable	

Core facilities	Information included in the manuscript?	In which section is the information available? (Reagents and Tools Table, Materials and Methods, Figures, Data Availability Section)
If your work benefited from core facilities, was their service mentioned in the acknowledgments section?	Not Applicable	

Design

Study protocol	Information included in the manuscript?	In which section is the information available? (Reagents and Tools Table, Materials and Methods, Figures, Data Availability Section)
If study protocol has been **pre-registered, provide DOI in the manuscript**. For clinical trials, provide the trial registration number **OR** cite DOI.	Not Applicable	
Report the **clinical trial registration number** (at ClinicalTrials.gov or equivalent), where applicable.	Not Applicable	

Laboratory protocol	Information included in the manuscript?	In which section is the information available? (Reagents and Tools Table, Materials and Methods, Figures, Data Availability Section)
Provide DOI OR other citation details if **external detailed step-by-step protocols** are available.	Yes	Materials and Methods

Experimental study design and statistics	Information included in the manuscript?	In which section is the information available? (Reagents and Tools Table, Materials and Methods, Figures, Data Availability Section)
Include a statement about **sample size** estimate even if no statistical methods were used.	Yes	Materials and Methods, Figures
Were any steps taken to minimize the effects of subjective bias when allocating animals/samples to treatment (e.g. **randomization procedure**)? If yes, have they been described?	Not Applicable	
Include a statement about **blinding** even if no blinding was done.	Not Applicable	
Describe **inclusion/exclusion criteria** if samples or animals were excluded from the analysis. Were the criteria pre-established?<br><br>If sample or data points were omitted from analysis, report if this was due to attrition or intentional exclusion and provide justification.	Not Applicable	
For every figure, are **statistical tests** justified as appropriate? Do the data meet the assumptions of the tests (e.g., normal distribution)? Describe any methods used to assess it. Is there an estimate of variation within each group of data? Is the variance similar between the groups that are being statistically compared?	Yes	Figures, Materials and Methods

Sample definition and in-laboratory replication	Information included in the manuscript?	In which section is the information available? (Reagents and Tools Table, Materials and Methods, Figures, Data Availability Section)
In the figure legends: state number of times the experiment was **replicated** in laboratory.	Yes	Figures
In the figure legends: define whether data describe **technical or biological replicates**.	Yes	Figures

Ethics

Ethics	Information included in the manuscript?	In which section is the information available? (Reagents and Tools Table, Materials and Methods, Figures, Data Availability Section)
Studies involving **human participants**: State details of **authority granting ethics approval** (IRB or equivalent committee(s), provide reference number for approval.	Not Applicable	
Studies involving **human participants**: Include a statement confirming that **informed consent** was obtained from all subjects and that the experiments conformed to the principles set out in the WMA Declaration of Helsinki and the Department of Health and Human Services Belmont Report.	Not Applicable	
Studies involving **human participants:** For publication of **patient photos**, include a statement confirming that consent to publish was obtained.	Not Applicable	
Studies involving experimental **animals**: State details of **authority granting ethics approval** (IRB or equivalent committee(s), provide reference number for approval. Include a statement of compliance with ethical regulations.	Not Applicable	
Studies involving **specimen and field samples:** State if relevant **permits** obtained, provide details of authority approving study; if none were required, explain why.	Not Applicable	

Dual Use Research of Concern (DURC)	Information included in the manuscript?	In which section is the information available? (Reagents and Tools Table, Materials and Methods, Figures, Data Availability Section)
Could your study fall under dual use research restrictions? Please check biosecurity documents and list of **select agents and toxins** (CDC): https://www.selectagents.gov/sat/list.htm	Not Applicable	
If you used a select agent, is the security level of the lab appropriate and reported in the manuscript?	Not Applicable	
If a study is subject to dual use research of concern regulations, is the name of the **authority granting approval and reference number** for the regulatory approval provided in the manuscript?	Not Applicable	

Reporting

The MDAR framework recommends adoption of discipline-specific guidelines, established and endorsed through community initiatives. Journals have their own policy about requiring specific guidelines and recommendations to complement MDAR.

Adherence to community standards	Information included in the manuscript?	In which section is the information available? (Reagents and Tools Table, Materials and Methods, Figures, Data Availability Section)
State if relevant guidelines or checklists (e.g., **ICMJE, MIBBI, ARRIVE, PRISMA**) have been followed or provided.	Not Applicable	
For **tumor marker prognostic studies,** we recommend that you follow the **REMARK** reporting guidelines (see link list at top right). See author guidelines, under 'Reporting Guidelines'. Please confirm you have followed these guidelines.	Not Applicable	
For **phase II and III randomized controlled trials**, please refer to the **CONSORT** flow diagram (see link list at top right) and submit the CONSORT checklist (see link list at top right) with your submission. See author guidelines, under 'Reporting Guidelines'. Please confirm you have submitted this list.	Not Applicable	

Data Availability

Data availability	Information included in the manuscript?	In which section is the information available? (Reagents and Tools Table, Materials and Methods, Figures, Data Availability Section)
Have **primary datasets** been deposited according to the journal's guidelines (see 'Data Deposition' section) and the respective accession numbers provided in the Data Availability Section?	Not Applicable	
Were **human clinical and genomic datasets** deposited in a public access-controlled repository in accordance to ethical obligations to the patients and to the applicable consent agreement?	Not Applicable	
Are **computational models** that are central and integral to a study available without restrictions in a machine-readable form? Were the relevant accession numbers or links provided?	Yes	Data Availability Section
If publicly available data were reused, provide the respective **data citations in the reference list**.	Yes	Results
